# Efficacy and Safety of Wuling Powder in the Treatment of Patients with Diabetic Nephropathy: A Systematic Review and Meta-Analysis

**DOI:** 10.1155/2022/1720749

**Published:** 2022-09-30

**Authors:** Yunyi Yang, Wenjun Sha, Keke Hou, Yuanying Xu, Shufa Tan, Hongping Yin, Lin Chen, Tao Lei

**Affiliations:** ^1^Putuo Hospital, Shanghai University of Traditional Chinese Medicine, Shanghai, China; ^2^Shaanxi University of Chinese Medicine, Xianyang, Shaanxi, China; ^3^Shanghai Putuo District Health Affairs Management Center, Shanghai, China

## Abstract

**Background:**

Wuling powder is a classical formula of traditional Chinese medicine (TCM), which is extensively applied to treat diabetic nephropathy (DN). However, there are no related reports on systematically evaluating the efficacy of Wuling powder in the treatment of DN. Targeted at this, this study was developed.

**Materials and Methods:**

This study systematically searched related articles from nine databases, including PubMed, Cochrane Library, Embase, Web of Science, China Knowledge Infrastructure (CNKI), China Biomedical CD-ROM (Sino Med), Wanfang database, Vipers database (VIP), and the China Clinical Trials Registry website. The randomized controlled trials (RCTs) involving Wuling Power to treat DN were included, which were published from the established data of the above databases to March 2022. In addition, the language of the studies was not restricted. Studies were meta-analyzed using the RevMan 5.4 software given in the Cochrane Collaboration Network. The treatment efficacy was measured using the weighted mean differences (WMD) and 95% confidence intervals (CI).

**Results:**

24 studies were included for the final analysis. 24 h urine volume (WMD = 357.95; 95% CI [322.83, 393.06], *p* < 0.00001), 24 h urine protein quantification(24 h UPQ) (WMD = −1.30; 95% CI [−1.82, −0.78], *p* < 0.00001), serum creatinine (Scr) (WMD = −10.17; 95% CI [−11.13, −9.21], *p* < 0.00001), blood urea nitrogen (BUN) (WMD = −1.62; 95% CI [−2.30, −0.93], *p* < 0.00001), urinary albumin excretion rate (UAER) (WMD = −24.73; 95% CI [−35.46, −13.99], *p* < 0.00001), fasting blood glucose (FBG) (WMD = −0.63.95% CI [−0.97, −0.30], *p* = 0.002), glycated hemoglobin (WMD = −0.11; 95% CI [−0.30, 0.08], *p*=0.26), total cholesterol (TC) (WMD = −0.63; 95% CI [−1.23, −0.04], *p*=0.04), triglycerides (TG) (WMD = −0.46. 95% CI [−0.70, −0.23], *p*=0.0001), high-density lipoprotein cholesterol (HDL-C) (WMD = −0.32; 95% CI [0.03, 0.62], *p*=0.03), low-density lipoprotein cholesterol (LDL-C) (WMD = −0.57; 95% CI [−0.77, −0.37], *p* < 0.00001), and total effective rate (TER) (response ratio (RR) = 1.40; 95% CI [1.32, 1.48]; *p* < 0.00001) were concluded. The Wuling powder in the treatment of DN was statistically significant in all the above outcome indicators, and the efficacy of the treatment group was better than that of the control group.

**Conclusion:**

The results of this study provided evidence for the clinical application of Wuling powder to treat the DN, but it had to be further validated in higher-quality clinical studies.

## 1. Introduction

Diabetic nephropathy (DN) is the most common and serious microvascular complication of diabetes mellitus (DM). It is clinically characterized by persistent albuminuria and/or progressive decline in glomerular filtration rate and microangiopathy, and it may result in end-stage renal disease in severe cases. Therefore, it becomes one of the leading causes of death in patients with DM [1]. The number of patients with DM in China ranks first all over the world, and its incidence is increasing year by year [2, 3]. In recent years, studies have shown that the incidence of nephropathy caused by DM has increased rapidly, surpassing glomerulonephritis and hypertension [4]. Once the DN has progressed to renal failure, it will be difficult to reverse, which seriously affects the life, health, and quality of life of patients, and brings serious mental and economic burdens to their families. Currently, angiotensin-converting enzyme inhibitors and angiotensin II receptor blockers can reduce the urinary protein, which can slow down the progression of DN, but its effect is not ideal [5]. Therefore, it is urgent to develop new treatment methods for DN.

**Figure 1:**
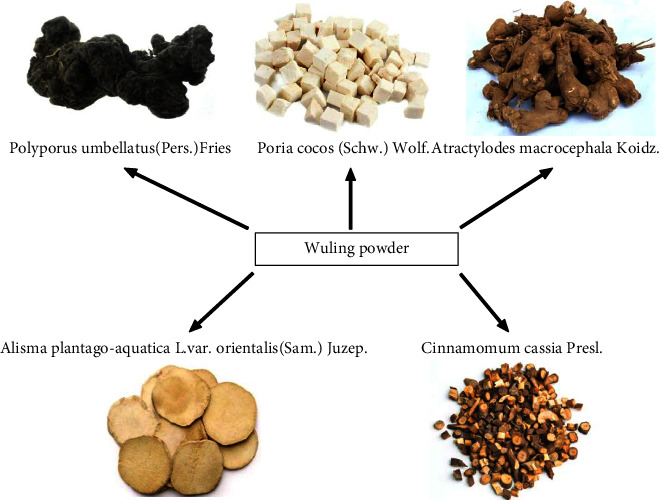
Compositions of Wuling powder.

The history of treating DN using Traditional Chinese medicine (TCM) is long, so the experience is highly enriched. In addition, the people-oriented and evidence-based TCM has achieved good clinical efficacy in the treatment of DN, and so it has become popular [6]. In TCM, DM is included in “oedema” and “thirst,” and its pathogenesis is determined as water metabolism disorders, Qi deficiency, blood stasis, paralysis, and obstruction of veins and ligaments, resulting in dysfunction of internal organs. As a classical TCM formula, Wuling powder was first recorded in the *Treatise on Miscellaneous Diseases of Typhoid Fever*, written by Zhang Zhong Jing, a famous doctor in the Eastern Han Dynasty. It can play the effect of dipping water and dampness, warming Yang, and transforming Qi. Wuling powder has been approved by the State Food and Drug Administration in China (approval number: Z11020702). The drug compositions of Wuling powder were as follows: Polyporus umbellatus (Pers.) Fries (zhū líng), Poria cocos (Schw.) Wolf (fú líng), Atractylodes macrocephala Koidz (bái zhú), Alisma plantago-aquatica L. var. orientalis (Sam.) Juzep (zé xiè), and Cinnamomum cassia Presl (guì zhī). The compositions and detailed summary of Wuling powder are given in Figure 1 and Table 1, respectively.

In this formula, Poria cocos Wolf, Polyporus umbellatus, and Alisma plantago-aquatica exerted the effect of diuresis and dampness percolation; Atractylodes macrocephala Koidz strengthened the spleen and transported damp, and it strengthened the spleen and dispelled damp together with Poria cocos Wolf; Cinnamomum cassia warmed Yang to help the bladder to transform Qi, so that water could flow on its own, which could not only lightly percolate the water and damp but also strengthen the spleen for water and damp transportation. Modern pharmacological studies have shown that Wuling powder has a protective effect on patients with DN, greatly improving the renal indicators such as creatinine clearance, urinary protein, and urea nitrogen level [7, 8]. Clinical studies have shown that Wuling powder combined with some specific western medicines can lower the urinary protein, promote the conversion of proteinuria and haematuria to negative, disperse the swelling, decrease the 24 h urinary protein volume, increase the average daily urine volume, regulate the immune inflammatory response, and better the lipid metabolism disorders and hypercoagulability in DN patients, with significant overall efficacy [9–11]. In this meta-analysis, an evidence-based approach was adopted to systematically evaluate the efficacy and safety of Wuling powder to treat DN, aiming to provide a more effective and reliable scientific basis for the clinical treatment of DN.

## 2. Methods

### 2.1. Design

The protocol for this systematic study was registered on the Open Science Framework (INPLASY) platform (https://inplasy.com/) (registration number: INPLASY202240071). It was implemented and executed according to the preferred reporting guidelines for systematic review and meta-analysis protocols [12]. The final report would be in line with the PRISMA recommendations for systematic view reporting of the medical interventions in meta-analysis [13].

### 2.2. Database and Literature Search

This study systematically searched nine databases from their establishment data to March 2022, including PubMed, Cochrane Library, Embase, Web of Science, China Knowledge Infrastructure (CNKI), China Biomedical CD-ROM (Sino Med), Wanfang database, Vipers database (VIP), and China Clinical Trials Registry website. In addition, the patent databases were searched to exclude the clinical trials that were not published due to patent applications for Wuling powder (approval number: Z11020702). The clinical randomized controlled trials (RCTs) focusing on Wuling powder to treat DN were included in this study. The main objective of the first search was to collect the literature comprehensively by taking “Wuling Powder,” “Wuling San,” “Wuling,” “Diabetic Nephropathies,” “Nephropathies, Diabetic,” “Nephropathy, Diabetic,” “Diabetic Nephropathy,” “Diabetic Kidney Disease,” “Diabetic Kidney Diseases,” “Kidney Disease, Diabetic,” “Kidney Diseases, Diabetic” “Diabetic Glomerulosclerosis,” “Glomerulosclerosis, Diabetic,” “Intracapillary Glomerulosclerosis, Diabetic.” “Intracapillary Glomerulosclerosis,” “Nodular Glomerulosclerosis,” “Glomerulosclerosis, Nodular,” “Kimmelstiel-Wilson Syndrome,” “Kimmelstiel Wilson Syndrome,” “Syndrome, Kimmelstiel-Wilson,” “Kimmelstiel-Wilson Disease,” “Kimmelstiel Wilson Disease,” “Kimmelstiel Wilson Syndrome,” “Kimmelstiel Wilson Disease,” “Kimmelstiel Wilson Disease,” “Kimmelstiel Wilson Disease,” and “Kimmelstiel Wilson Disease” as the search terms. The search strategy was shown in Supplementary Material Table 1.

### 2.3. Inclusion and Exclusion Criteria

#### 2.3.1. Types of Research

The selected RCTs were on the Wuling powder plus or minus formula for the treatment of DN.

#### 2.3.2. Subjects of the Studies

In the included studies, the subjects were patients who met the internationally recognized diagnostic criteria for DN at the time of the study, who had been definitively diagnosed with DN by a clinician, and who had excluded primary nephropathy and other causes of renal disease.

#### 2.3.3. Interventions

All patients in the treatment group were treated with Wuling powder plus or minus formula, while those in the control group received other hypoglycaemic drugs; or patients in both treatment and control groups received the same conventional diabetes medication and based on which patients in the treatment group took Wuling powder plus or minus formula. Studies with multiple interventions or where Wuling powder was not the primary intervention were excluded. There were no requirements for the course of the disease, course of treatment, and dose of medication.

#### 2.3.4. Outcome Indicators

The main outcome indicators in this study included 24 h urine volume; 24 h urinary protein quantification (24 h UPQ); serum creatinine (Scr); blood urea nitrogen (BUN); urinary albumin excretion rate (UAER); and total effective rate (TER). The secondary outcome indicators were fasting blood glucose (FBG); hemoglobin A1c; total cholesterol (TC); triglyceride (TG); high-density lipoprotein cholesterol (HDL-C); and low-density lipoprotein cholesterol (LDL-C). In addition, safety was measured based on adverse effects.

#### 2.3.5. Exclusion Criteria

The studies satisfying the below conditions had to be excluded: (1) duplicate publications; (2) reviews, summaries of expert experience, evaluative articles, or theoretical elaborations; (3) not RTCs, or animal studies; (4) nonclinical studies such as pharmacology; and (5) with incomplete documentation of data.

### 2.4. Data Collection and Analysis

#### 2.4.1. Literature Screening

The RCTs were screened independently by two researchers to exclude those who failed to meet the inclusion criteria. After the elimination of the duplicates, the abstracts of the searched RCTs were read for initial screening to exclude the RCTs not meeting the inclusion criteria and all the RCTs were downloaded. Next, the full texts of these RCTs were read. Finally, the RCTs on Wuling powder for DN treatment that met the inclusion criteria were selected. Any different opinions between the two researchers were referred to a third party for adjudication.

#### 2.4.2. Research Data Extraction

The authors, year of publication, mean age, number of trials and controls, course of the disease, number of cases by gender, interventions, duration of treatment, and outcome indicators of the included RCTs were selected. Then, the authors were contacted if the required data were incomplete. Two researchers cross-checked the information entered in the RCTs, and any disagreements were referred to a third party for adjudication.

#### 2.4.3. Assessment on Risk of Bias

The risk of bias of the included RCTs was assessed by using the Risk of Bias Assessment Tool recommended by the Cochrane Collaboration, in terms of seven aspects: (1) random sequence generation method; (2) allocation protocol concealment; (3) blindness to subjects and intervention providers; (4) blindness to outcome assessors; (5) completeness of outcome data; (6) selective reporting of study results; and (7) other biases. For each of the included RCTs, “high risk,” “low risk,” and “unclear risk” were assessed for each aspect [14]. In case of any disagreement on assessment results of the risk of bias, it should be discussed with a third researcher.

#### 2.4.4. Data Analysis

The RevMan 5.4 software provided by the Cochrane Collaboration Network was adopted for meta-analysis. Dichotomous variables were expressed with the response ratio (RR), and the continuous variables were expressed using mean differences (MD). The *χ*^2^ test was performed for heterogeneity. If *p* > 0.1 and *I*^2^ < 50%, the heterogeneity among all RCTs was low and the fixed effect model (FEM) was used for meta-analysis; while if *p* < 0.1 and *I*^2^ ≥ 50%, the heterogeneity was statistically significant and the random effect model (REM) was used for the meta-analysis. *p* < 0.05 indicated a statistically significant difference.

#### 2.4.5. Heterogeneity Assessment and Sensitivity Analysis

If *I*^2^ ≥ 50%, there was a statistical heterogeneity and the REM was adopted to analyse the data; while the FEM was adopted if the test for heterogeneity was not significant (*I*^2^ < 50%). As there was a variation in heterogeneity, the sensitivity analysis or subgroup analysis was essential to explore the potential causes of heterogeneity and to exclude the RCTs with a high risk of bias, so as to ensure the robustness of results.

#### 2.4.6. Subgroup Analysis

Due to differences in heterogeneity, subgroup analysis was required to analyze the possible reasons for heterogeneity. The main subgroup analysis items included different ages, control treatments, duration of treatment, region, and safety.

#### 2.4.7. Assessment of Publication Bias

Publication bias was detected using a funnel plot. A significant asymmetry in the funnel plot meant a publication bias.

## 3. Results

### 3.1. Literature Search

**Figure 2:**
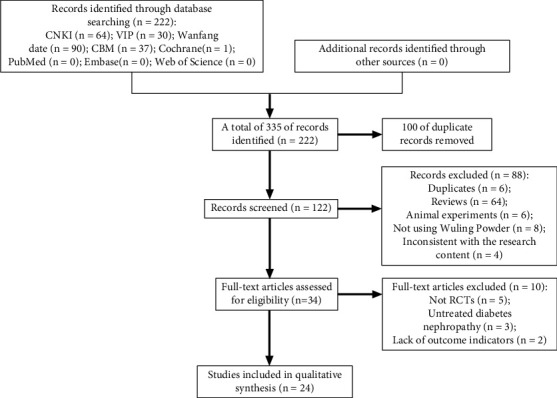
Flowchart of specific publications screening.

Figure 2 showed the flowchart to screen the RCTs. 222 relevant publications were searched from various databases, and 100 duplicates were excluded after the initial screening. Next, 88 not satisfying the inclusion criteria were excluded. Then, after the full texts were read carefully, the publications which were not RCTs, not related to the treatment of DN, and lacked details of the results were excluded. Finally, 24 RCTs [15–38] were included in this meta-analysis.

### 3.2. Characteristics of the Included RCTs

2,018 patients were included in the 24 RCTs, including 1,030 in the treatment group and 988 in the control group. The mean age of the patients ranged from 50.57 ± 15.17 years old to 69.58 ± 1.65 years old. The intervention for patients in all treatment groups was Wuling powder combined with conventional Western medicine. All 24 RCTs were conducted in China and published in 2003∼2021. The shortest and longest durations of treatment were 3 weeks and 12 weeks, respectively.  Table 2 showed in detail the basic characteristics of the 24 RCTs.

### 3.3. Risk of Bias in the Included RCTs

**Figure 3:**
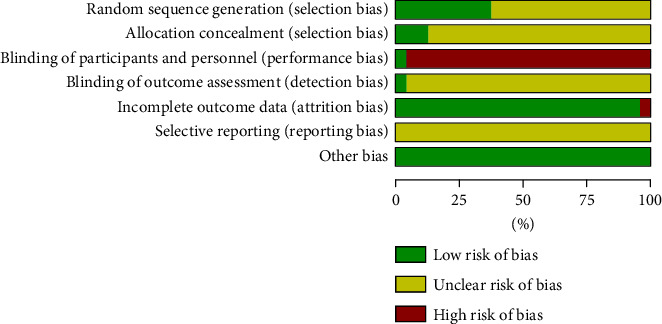
Risk of bias.

**Figure 4:**
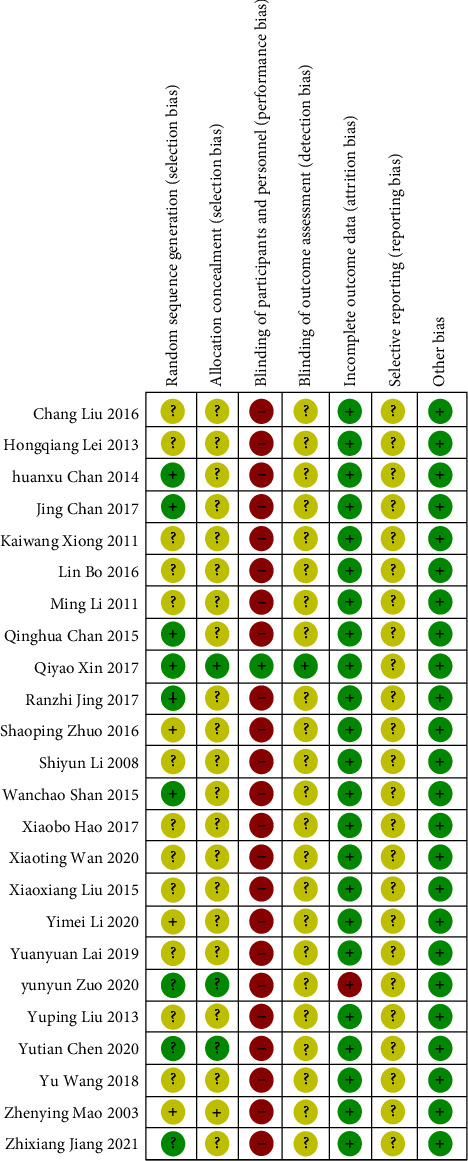
Summary chart of risk of bias.

This meta-analysis investigated the risk of bias for all RCTs included. All research projects were randomized into a five-linger group and a control group. A comparison of the 24 RCTs revealed inconsistent randomization of treatment; 9 RCTs [21–23, 28, 30, 31, 34, 36, 38] were determined as low risk of bias because they generated random sequences by random number tables and random coin flips, while the remaining RCTs described only “random allocation” and therefore were determined to be an unclear risk of bias. In addition, 3 RCTs [31, 34, 36] reported allocation concealment, so they were classified as low risk of bias. In terms of performance bias, only 1 RCT [31] reported the double-blind trials, so the remaining 23 RCTs were classified as having a high risk of bias. In terms of reporting bias, only 1 RCT [34] had shedding of participant data and was, therefore, determined to have a high risk of bias. All RCTs were balanced at baseline examination and showed no other bias. The full and detailed analysis results on the risk of bias are shown in Figures 3 and 4.

### 3.4. Results

#### 3.4.1. 24 h Urine Volume

**Figure 5:**
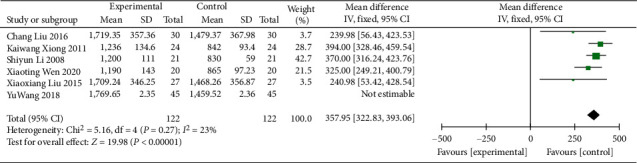
Forest plot of 24 h urine volume.

6 RCTs [16, 17, 24, 25, 32, 37] involving 334 DN patients provided data on 24 h urine volume before and after the intervention. A heterogeneity test (*P* = 0.03 and I^2^ = 59%) and a sensitivity analysis revealed that the statistical heterogeneity became lower after the study of Wang et al. [32] was removed (*χ*^2^ = 5.16, *p*=0.27, I^2^ = 23%; Figure 5), so the FEM was used. The results showed that the 24 h urine volume was significantly higher for patients in the treatment group treated with Wuling powder combined with conventional western medicine (WMD = 357.95; 95% CI [322.83, 393.06], *p* < 0.00001; Figure 5). The subgroup analysis suggested that, in the different age subgroups (*p*=0.01), the group <60 years (*χ*^2^ = 3.52, *p*=0.32, I^2^ = 15%) was homogeneous with the group ≥60 years (*χ*^2^ = 0.56, *p*=0.45, I^2^ = 0%), and the difference was statistically significant. The difference between different regional subgroups (*p*=0.14) was not significant (Table 3, Supplementary Material Figures 1 and 2).

#### 3.4.2. 24 h UPQ

**Figure 6:**
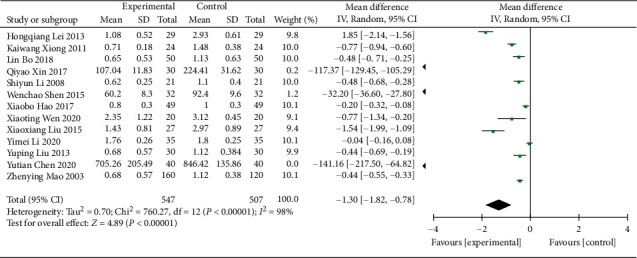
Forest plot for 24 h UPQ.

13 RCTs [15–17, 19, 20, 23, 24, 26, 29, 31, 35–37] (including 1,054 patients) reported the data of 24 UPQ. Significant heterogeneity was found in these 13 RCTs (*χ*^2^ = 760.27, *p* < 0.00001, I^2^ = 98%; Figure 6), so the REM was used. The 24 h UPQ for patients treated with Wuling powder combined with conventional western medicine was higher than that in the control group (WMD = -1.30; 95% CI [−1.82, −0.78], *p* < 0.00001; Figure 6). Because of the large heterogeneity, the subgroup analysis was conducted based on the age, control treatment, duration of treatment, and region; and significant differences were found in intervention effects of different ages (*p*=0.002), control treatments (*p* < 0.0001), and regions (*p* < 0.0001). It was reflected in the glipizide group (control treatment) (*χ*^2^ = 0.1, *p*=0.95, I^2^ = 0%) and in Guangdong province (region) (*χ*^2^ = 0.36, *p*=0.55, I^2^ = 0%), while significant heterogeneity was still observed in the rest (Table 3, Supplementary Material Figures 3–6).

#### 3.4.3. Scr

**Figure 7:**
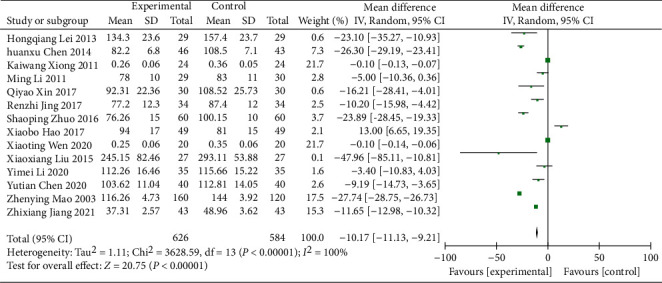
Forest plot of Scr.

**Figure 8:**
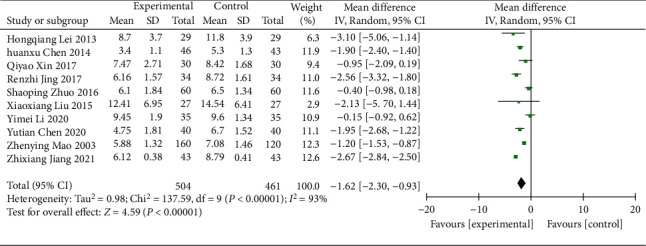
Forest plot of BUN.

14 RCTs [15, 17–19, 21, 24, 27–29, 31, 35–38] (including 1,210 participants) reported the Scr data. Significant heterogeneity was found (*χ*^2^ = 3628.59, *p* < 0.00001, I^2^ = 100%; Figure 7), so the meta-analysis was conducted by using a REM. The analysis showed a statistically significant difference in Scr between the treatment and control groups (WMD = −10.17; 95% CI [−11.13, −9.21], *p* < 0.00001; Figure 7), indicating that the Scr was significantly better in the treatment group. In addition, the subgroup analysis was conducted in different durations of treatment and regions, and different durations of treatment showed no statistically significant difference (*p*=0.11) and different regions showed statistically obvious difference (*p*=0.007), but significant heterogeneity was still observed (Table 3, Supplementary Material Figures 7 and 8).

#### 3.4.4. BUN

**Figure 9:**
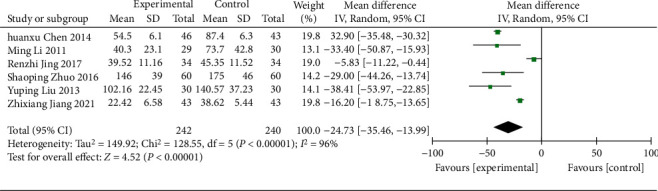
Forest plot of UAER.

**Figure 10:**
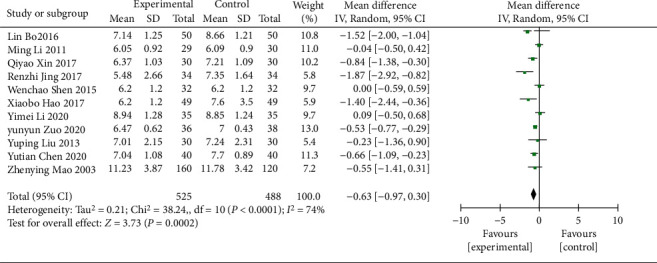
Forest plot of FBG.

10 RCTs [15, 19, 21, 24, 27, 28, 31, 35, 36, 38] (including 965 patients) reported the BUN data. The tests showed significant heterogeneity (*χ*^2^ = 137.59, *p* < 0.00001, I^2^ = 93%; Figure 8), so the meta-analysis was conducted by using a REM. The analysis showed a statistically significant difference in BUN between the treatment and control groups (WMD = −1.62; 95% CI [−2.30, −0.93], *p* < 0.00001; Figure 8), indicating that the BUN was significantly better in the treatment group. Subgroup analysis in different durations of treatment and regions showed no significant difference in intervention effects between the two groups (*p*=0.26 and 0.41 respectively), but significant heterogeneity was still observed (Table 3, Supplementary Material Figures 9 and 10).

#### 3.4.5. UAER

**Figure 11:**
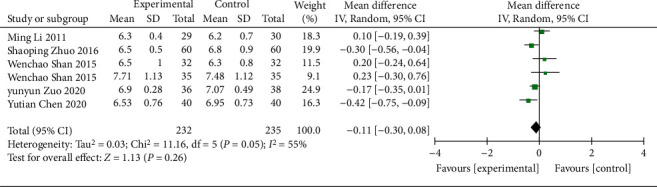
Forest plot of glycated hemoglobin.

**Figure 12:**
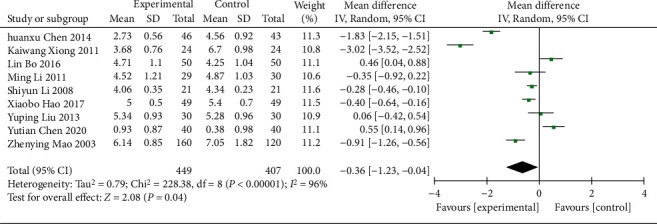
Forest plot of TC.

**Figure 13:**
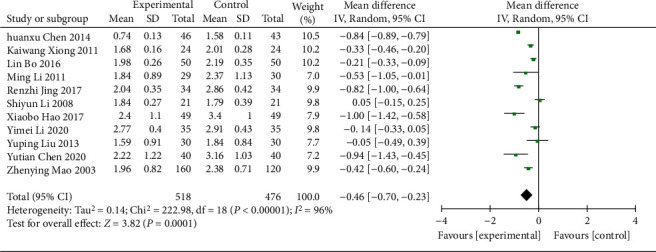
Forest plot of TG.

6 RCTs [18, 20, 21, 27, 28, 38] (including 482 patients) reported on UAER. Tests showed significant heterogeneity (*χ*^2^ = 128.55, *p* < 0.00001, I^2^ = 96%; Figure 9), so a REM was used. A statistically significant difference was found in UAER between the treatment and control groups (WMD = −24.73; 95% CI [−35.46, −13.99], *p* < 0.00001; Figure 9), indicating that the UAER was significantly better in the treatment group. A subgroup analysis suggested the intervention effect was not significantly different in age (*p*=0.11) and duration of treatment (*p*=0.93) but showed an obvious difference in regions (*p*=0.02), with no heterogeneity in the Guangdong region (*χ*^2^ = 0.24, *p*=0.62, I^2^ = 0%) and the rest still observed significant heterogeneity (Table 3, Supplementary Material Figures 11–13).

#### 3.4.6. FBG

**Figure 14:**
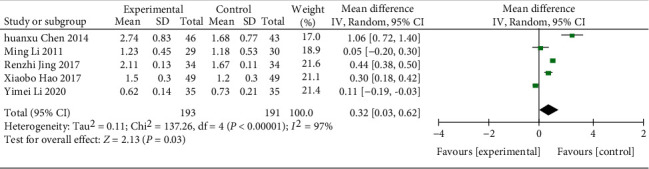
Forest plot of HDL.

**Figure 15:**
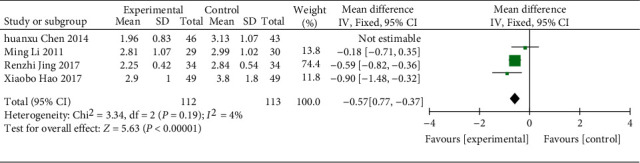
Forest plot of LDL.

**Figure 16:**
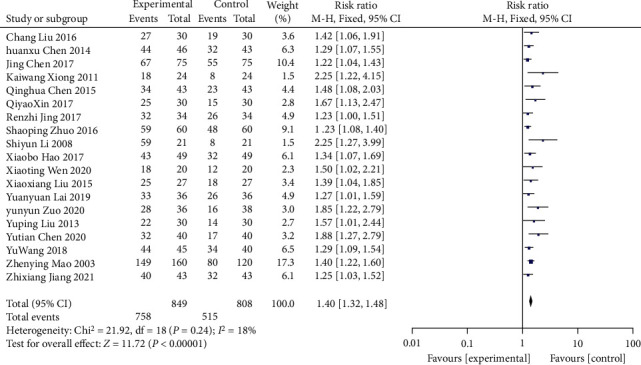
Forest plot of TER.

**Figure 17:**
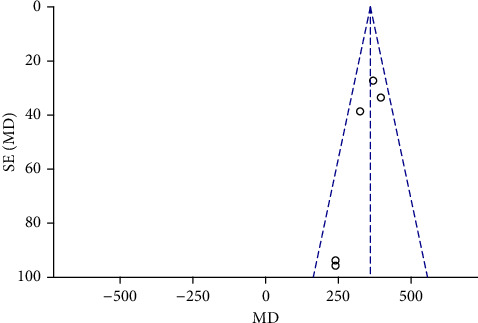
The publication bias funnel chart of 24 h urine volume.

**Figure 18:**
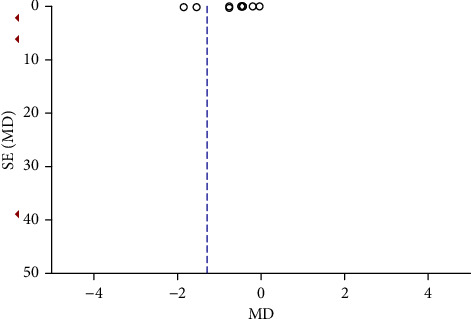
The publication bias funnel chart of 24 h UPQ.

11 RCTs [15, 18, 20, 23, 26, 28, 29, 31, 34–36] (including 1,013 patients) reported the FBG data. Tests showed significant heterogeneity (*χ*^2^ = 38.24, *p* < 0.0001, I^2^ = 74%; Figure 10), so a REM was adopted for meta-analysis. The analysis results revealed a statistically significant difference in FBG between the treatment and control groups (WMD = −0.63; 95% CI [−0.97, −0.30], *p*=0.002; Figure 10), suggesting that Wuling powder combined with conventional western medicine may significantly reduce the FBG in DN patients. Based on different ages, control treatments, durations of treatment, regions, and safety, the subgroup analysis was conducted, and the results showed no significant differences in intervention effects (*p*=0.49, 0.45, 0.49, 0.67, and 0.78, respectively); no heterogeneity was found in the ≥60 years group (*χ*^2^ = 0.26, *p*=0.61, I^2^ = 0%) in the age subgroup and in Guangdong Province (*χ*^2^ = 0.26, *p*=0.61, I^2^ = 0%) in the region, while significant heterogeneity was still observed in the rest (Table 3, Supplementary Material Figures 14–18).

#### 3.4.7. Glycated Hemoglobin

**Figure 19:**
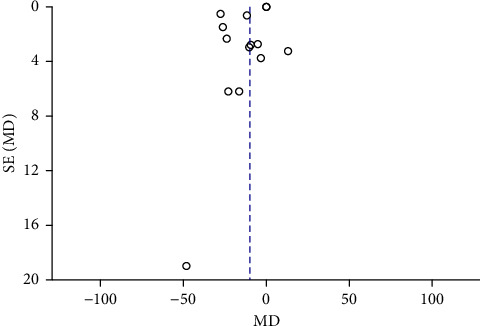
The publication bias funnel chart of Scr.

**Figure 20:**
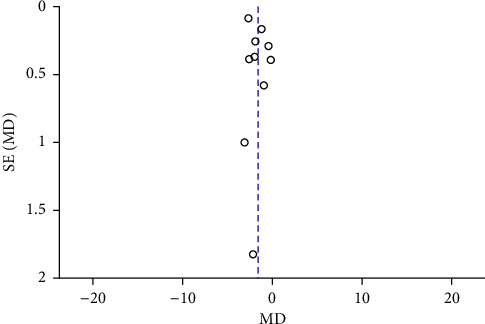
The publication bias funnel chart of BUN.

6 RCTs [18, 23, 27, 34–36] (including 467 patients) reported the glycated hemoglobin. A REM was adopted because significant heterogeneity was found among them (*χ*^2^ = 11.16, *p*=0.05, I^2^ = 55%; Figure 11). The glycated hemoglobin between the treatment and control groups was statistically significant (WMD = −0.11; 95% CI [−0.30, 0.08], *p*=0.26; Figure 11), indicating that it was significantly better in patients treated with the Wuling powder combined with conventional western medicine. Subgroup analysis showed no significant difference in intervention effects in age (*p*=0.56) and region (*p*=0.01), with no heterogeneity in Guangdong Province (*χ*^2^ = 0.32, *p*=0.57, I^2^ = 0%), while significant heterogeneity was still observed in the rest (Table 3, Supplementary Material Figures 19 and 20).

#### 3.4.8. Blood Lipids

**Figure 21:**
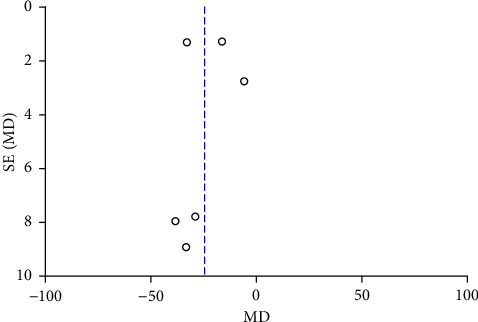
The publication bias funnel chart of UAER.

**Figure 22:**
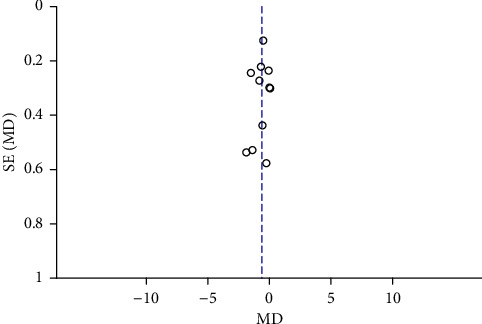
The publication bias funnel chart of FBG.

**Figure 23:**
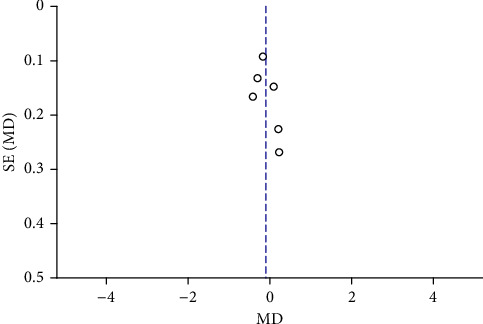
The publication bias funnel chart of glycated hemoglobin.

**Figure 24:**
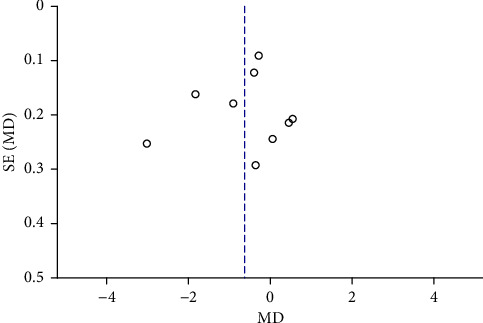
The publication bias funnel chart of TC.

**Figure 25:**
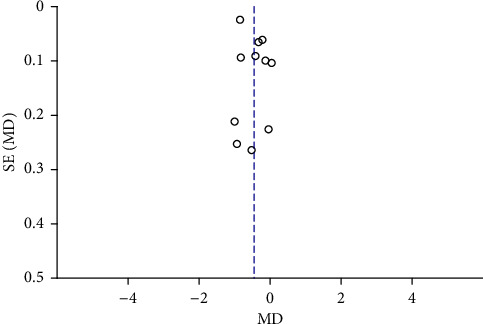
The publication bias funnel chart of TG.

**Figure 26:**
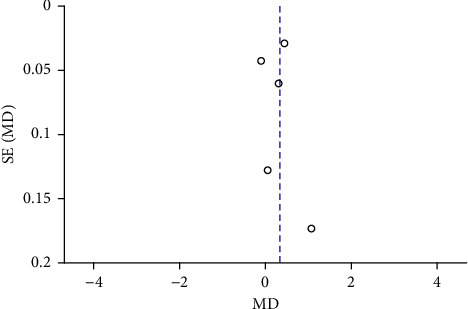
The publication bias funnel chart of HDL-C.

**Figure 27:**
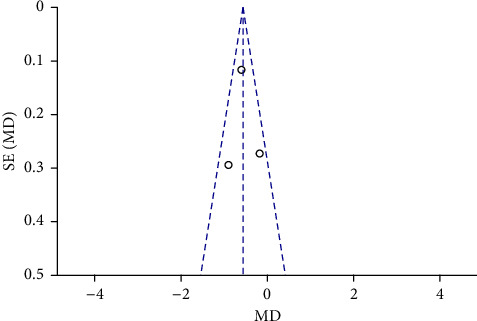
The publication bias funnel chart of LDL-C.

**Figure 28:**
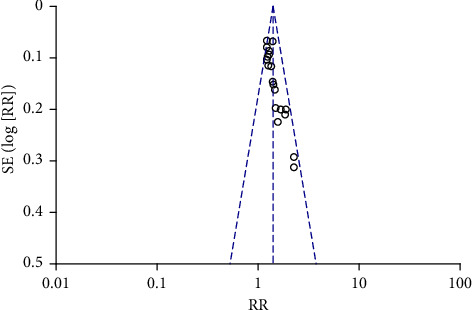
The publication bias funnel chart of TER.

(1) 9 RCTs [15–18, 20, 21, 26, 29, 36] (856 patients) reported the TC and high heterogeneity was found (*χ*^2^ = 228.38, *p* < 0.00001, I^2^ = 96%; Figure 12). The REM analysis results showed the TC was statistically significant (WMD = −0.63; 95% CI [−1.23, −0.04], *p*=0.04; Figure 12). In the subgroup analysis, the differences in age, control treatment, duration of treatment, and region (*p*=0.06, 0.22, 0.05, and 0.99, respectively) were not statistically significant (Table 3, Supplementary Material Figures 21–24).

(2) 11 RCTs [15–18, 20, 21, 26, 28, 29, 35, 36] (994 patients) reported TG values and the heterogeneity was high (*χ*^2^ = 222.98, *p* < 0.00001, I^2^ = 96%; Figure 13). The REM analysis results showed (WMD = −0.46; 95% CI [−0.70, −0.23], *p*=0.0001; Figure 13) the differences in TG between the two groups were statistically significant. In the subgroup analysis, the differences were not statistically significant in age, control treatment, and duration of treatment (*p*=0.08, 0.11, and 0.7, respectively), were statistically significant in regions (*p* < 0.00001), and not heterogeneous in Guangdong Province (*p*=0.69, I2 = 0%) (Table 3, Supplementary Material Figures 25–28).

(3) RCTs [18, 21, 28, 29, 35] (384 patients) recorded HDL values with a test for heterogeneity (*χ*^2^ = 137.26, *p* < 0.00001, I2 = 97%; see Figure 14), indicating high heterogeneity, and using a random effects model, the results showed (WMD = −0.32; 95% CI [0.03,0.62], *p*=0.03; see Figure 14), the difference between the two groups was statistically significant. In the subgroup analysis, the differences were statistically significant across sessions and regions (all *p*=0.0002) (Table 3, Supplementary Material Figures 29 and 30).

(4) 4 RCTs [18, 21, 28, 29] (314 patients) recorded LDL values, and the heterogeneity among them was proved to be high (*χ*^2^ = 10.28, *p*=0.02, I^2^ = 71%; Figure 15), which became lower after the study by Chen et al. [21] was removed (*χ*^2^ = 3.34, *p* = 0.19, I^2^ = 40%; Figure 15), so the difference between the two groups was statistically significant using a FEM (WMD = −0.57; 95% CI [−0.77, −0.37], *p* < 0.00001). In the subgroup analysis, the differences were statistically significant in control treatment and region (all *p*=0.02) (Table 3, Supplementary Material Figures 31 and 32).

In summary, Wuling powder showed better performance compared with the conventional methods in the treatment of DN in terms of TC, TG, HDL-C, and LDL-C.

#### 3.4.9. TER

The TER was categorized as markedly effective, effective, and ineffective according to the improvement degree in clinical symptoms and related indicators (mainly 24 h urine volume, 24 h UPQ, Scr, BUN, UAER, and FBG). 19 RCTs [15–17, 20–22, 24, 25, 27–34, 36–38] involving 1,657 patients undertook the TER as an outcome indicator. As there was no significant heterogeneity (*χ*^2^ = 21.92, *p*=0.24, I^2^ = 18%; Figure 16), an FEM was used. The analysis showed that Wuling powder resulted in a significant increase in TER compared to conventional treatment (RR = 1.40; 95% CI [1.32, 1.48]; *p* < 0.00001; Figure 16). Subgroup analysis based on age, control treatment, duration of treatment, and region showed no significant differences (*p*=0.72, 0.45, 0.37, and 0.79, respectively) (Table 3, Supplementary Material Figures 33–36).

### 3.5. Adverse Effects

Adverse reactions were reported in 2 out of 24 RTCs. The adverse reactions reported in the study by Shen and Shu [23] were nausea, vomiting, abdominal distension, diarrhea, skin rash, and mild hypoglycemia; while those reported by Jing et al. [28] included nausea, vomiting, back pain, skin pruritus, swelling of lower limbs, thirst, and excessive drinking. No adverse reactions were reported in the remaining RCTs.

### 3.6. Publication Bias

Funnel plots were plotted for studies with >10 literature on combined outcome indicators, and 24 h urine volume, 24 h UPQ, Scr, BUN, UAER, FBG, glycated hemoglobin, TC, TG, HDL-C, LDL-C, and TER after treatment showed significant asymmetry in the funnel plots (Figures 17–28), indicating publication bias in the included studies.

### 3.7. Certainty of Evidence

The GRADEpro was employed to assess the certainty of the evidence in this study.  Table 4 showed that the results of 24 h urine output, LDL, and TER were moderate-quality evidence, while other outcomes were low-quality evidences. The high heterogeneity of some outcomes, the low methodological quality, and the high risk of bias were reasons for the poor quality of the evidence. Therefore, Wuling powder should be considered cautiously in the clinical use for DN treatment.

## 4. Discussion

### 4.1. Results

As one of the common vascular disease complications in DM patients, DN seriously affects the prognosis of patients and should be treated as early as possible. In recent years, TCM has become an essential adjunctive drug treatment for most Chinese patients with DN due to its stable efficacy and low side effects. Many studies confirm that the combination of Wuling powder with conventional symptomatic supportive treatment for DN is effective in alleviating the clinical symptoms, improving renal function, stabilizing FBG, and lowering TG. It is safe and reliable with good clinical application and promotion value.

This study included 24 studies and found that Wuling powder exerted a positive effect on the clinical management of DN. This meta-analysis study provided a sound theoretical basis for the application of Wuling powder in the treatment of DN. Therefore, the results of this study may provide an important reference for the adjuvant treatment of DN with TCM.

Although our results were statistically significant, some of the results were subject to greater heterogeneity. The outcome markers were divided into subgroups based on characteristics of the patients such as different age, control treatment, duration of treatment, and region for comparison to seek reasons for heterogeneity. In the subgroup analysis, 24 h urine volume reduced heterogeneity after different age subgroup analysis, 24 h urine protein quantification reduced heterogeneity after control treatment and regional subgroup analysis, urine albumin excretion rate reduced heterogeneity after regional subgroup analysis, FBG reduced heterogeneity after age and regional subgroup analysis, and glycated hemoglobin and TG both reduced heterogeneity after regional subgroup analysis. It is worth noting that most of the outcome indicators showed lower heterogeneity in the Guangdong region, which may be related to the origin of the herbs. In contrast, significant heterogeneity was observed in Scr and BUN. The subgroup analysis was performed and there were no significant differences in intervention effects between groups, and the size of such heterogeneity was not reduced following the use of a REM.

It is believed that these heterogeneities arise from the following points. Firstly, the reasons for the large heterogeneity are most likely related to the variety, origin, harvesting season, storage and processing, dosage form, and route of administration of the herbal medicines. Such contents are not described in detail in the literature, So they could not be analyzed further in this study. Secondly, only 24 relevant studies were included in this study, and most of them only mentioned the word simple randomisation without considering the specific implementation methods, which affected the scientific validity of the study results. In addition, three studies [31, 34, 36] reported allocation concealment and only one study [31] reported the use of a double-blind trial. The rest studies did not report the allocation concealment and were unblinded, which was susceptible to a variety of artifacts and may lead to heterogeneity with different study participants and various interventions. The randomization grouping resulted in selective bias, which could reduce the overall quality of the meta-analysis. Furthermore, some of the included studies did not mention the methods of testing for outcome indicators, and there were uncontrollable factors such as different experimental instruments, which affected the objectivity of the results. In addition, one study [34] had a shedding of participant data, which may affect the final analysis of the results. Meanwhile, some of the outcome indicators were combined despite high heterogeneity, which affected the reliability of the study. Taken together, these may have contributed to the high heterogeneity of some of the outcome indicators.

In addition, many other factors were evident in this study in terms of their impacts on the results. Firstly, the studies included in this study were limited to English and Chinese, and the final analysis was conducted on all Chinese literature, which would result in a potential publication bias. While evidence-based treatment is a core aspect of TCM interventions, TCM places importance on the etiology of the disease. The studies included in this study tended to apply a specific drug without considering the individuality, diversity, and complexity of the DN, making it difficult to determine whether people in different studies had achieved true evidence-based treatment. Therefore, it is one of the larger reasons why the results were affected. In addition, there were different conventional Western medical treatments in the included studies. For example, some patients may also receive conventional treatments such as hypotension and lipid-lowering depending on their conditions. However, some of the studies failed to explain in detail what kind of Western medical treatment was adopted. In addition, whether the dose was controlled strictly would also be another factor resulting in publication bias. Clinical trials are concerned with the follow-up of patients' long-term outcomes. Most of the included studies were limited to a short-term treatment after the drug intervention, which also impacts the bias in outcome efficacy. In the subgroup analysis, the cut-off points for age and duration of disease were mainly based on relevant studies, however, more biological support is needed. Finally, the funnel plot showed the publication bias in this study, which may be due to the ease of publishing positive results and the difficulty of publishing negative results, severely limiting the validity and objectivity of the efficacy of Wuling powder to treat DN. In addition, the results of the GRADE analysis showed that the reliability of the outcome indicators was mostly low to moderate. Therefore, Wuling powder could be cautiously recommended as an adjunctive treatment for DN.

### 4.2. Strengths and Limitations

The strengths of this study could be summarized as follows. Firstly, this was probably the first meta-analysis to assess the efficacy and safety of Wuling powder in the treatment of DN. Secondly, all the literature included were RCTs, ensuring the credibility of the results of this study. Thirdly, the results of this study provided a new therapeutic option for the treatment of DN. The results of this study suggested that the combination of the Wuling powder with conventional treatment for DN had positive clinical implications, which was superior to the Western medicine treatment alone. It implied that Wuling powder may enhance the effectiveness of conventional treatment and improve the overall clinical outcome, reflecting the uniqueness and superiority of TCM. Due to the holistic treatment theory, the use of TCM in the adjunctive treatment of disease is increasingly reported and researched. It is found that TCM can play a better therapeutic advantage in the treatment of both DN and its complications, and exert a positive effect on the safety, suffering reduction, and life improvement of patients. Systematically assessing the efficacy of TCM in DN and providing corresponding evidence-based medical evidence are of high significance to promote the TCM culture worldwide and search for new breakthroughs in the treatment of DN patients.

However, there are some limitations to this study. Firstly, it was limited by the quality of the literature. Most of the studies included in the study did not report allocation concealment and the use of blinding, leading to the measurement and implementation of various biases. Secondly, the included studies were RCTs with small samples and were of low quality. Thirdly, the lack of DN staging in most of the studies in this study affected the effectiveness of Wuling powder in patients with different degrees of DN. In addition, the lack of a placebo prevented us from analysing the difference in efficacy between using and not using Wuling powder. Finally, the patients in this study were all selected from the Chinese region and may not be globally representative, with some degree of clinical bias applied.

## 5. Conclusion

In conclusion, Wuling powder combined with conventional drugs showed outstanding efficacy and positive effect in the treatment of DN. However, there were still some limitations in this systematic evaluation, so applying Wuling powder in clinical treatment should be considered cautiously. Therefore, some clinical studies with larger samples, higher study quality, and more rigorous study design should be taken in the future to validate the accurate and objective assessment of DN, and then obtain more valuable meta-analysis results, providing more reliable and effective new ideas for the treatment of DN.

## Figures and Tables

**Table 1:** Details of the compositions of Wuling powder.

Chinese herbs	Latin name	Family	Part of herbs	Functions in TCM
Zhu ling (zhū líng)	*Polyporus umbellatus* (Pers.) Fries	Sargassaceae	Sclerotium	To clear dampness and promote diuresis
Fu ling (fú líng)	*Poria cocos* (Schw.) Wolf.	Polyporaceae	Sclerotium	To clear dampness, promote diuresis, strengthen the spleen, and calm the heart
Bai Zhu (bái zhú)	*Atractylodes macrocephala* Koidz.	Asteraceae	Roots and rhizomes	To invigorate Qi and spleen, dry damp, stop sweating, and relieve miscarriage
Ze Xie (zé xiè)	*Alisma plantago-aquatica* L.var. orientali s(Sam.) Juzep.	Alismataceae	Roots and rhizomes	To dry damp, release heat, resolve turbidity, and lower lipids
Gui Zhi(guì zhī)	*Cinnamomum cassi*a Presl.	Lauraceae	Twig	To sweat and relieve the surface, dispel cold and relieve pain, and clear the Yang and transform Qi

**Table 2:** Basic characteristics of the included RCTs.

Author, year	Age (T/C)	Number (T/C)	Duration of disease (T/C, year)	Gender (M/F)	Intervention (T)	Intervention (C)	Course	Outcomes	Region
Zhenying Mao 2003	58.13/56.14 ± 0	280 (160/120)	2.11/1.98	T (76/84) C (69/51)	Wuling powder + RT	Gliquidone	12 w	②③④⑥⑧⑨⑫	Puyang, Henan
Shiyun Li 2008	52.35/51.15	42 (21/21)	0.88/4.3	T (13/8) C (12/9)	Wuling powder + RT	RT	3–4 w	①②⑧⑨⑫	Zhumadian, Henan
Kaiwang Xiong 2011	54.5	48 (24/24)	3∼16	26/22	Wuling powder + RT	RT	4 w	①②③⑧⑨⑫	Fenyi, Jiangxi
Ming Li 2011	(57.6 ± 7.1)/(55.8 ± 7.2)	59 (29/30)	(7.9 ± 5.8)/(7.8 ± 6.5)	T (15/14) C (16/14)	Wuling powder + RT	RT	12 w	③⑤⑥⑦⑧⑨⑩⑪	Shanghai
Hongqiang Lei 2013	(53.4 ± 10.8)/(52.3 ± 10.5)	58 (29/29)	NR	T (17/12) C (16/13)	Wuling powder + RT	RT	12 w	②③④	Weinan, Shaanxi
Yuping Liu 2013	(45∼82)/(42∼80)	60 (30/30)	5/5.5	T (20/10) C (18/12)	Wuling powder + RT	Gliquidone	8 w	②⑤⑥⑧⑨⑫	Cangwu, Guangxi
Huanxu Chen 2014	(52.1 ± 6.3)/(51.3 ± 6.5)	89 (46/43)	(6.1 ± 1.5)/(5.7 ± 1.3)	T (27/19) C (25/18)	Wuling powder + RT	RT	2 w	③④⑤⑧⑨⑩⑪⑫	Zhongshan, Guangdong
Qinghua Chen 2015	(54.9 ± 7.1)/(53.5 ± 6.3)	86 (43/43)	(11.1 ± 1.0)/(10.4 ± 1.6)	T (27/16) C (25/18)	Wuling powder + RT	RT	4 w	⑫	Yunfu, Guangdong
Wenchao Shen 2015	(53.7 ± 4.2)/(52.8 ± 4.1)	64 (32/32)	(6.8 ± 1.2)/(6.9 ± 1.3)	T (17/15) C (15/17)	Wuling powder + RT	RT	8 w	②⑥⑦⑬	Pinghu, Zhejiang
Xiaoxiang Liu 2015	(58.2 ± 6.3)/(59.1 ± 5.4)	54 (27/27)	(9 ± 1.3)/(10 ± 1.6)	T (12/15) C (13/14)	Wuling powder + RT	RT	3 w	①②③④⑫	Beijing
Chang Liu 2016	(60.5 ± 5.5)/(59.3 ± 5.2)	60 (30/30)	(8.9 ± 1.1)/(9.2 ± 1.1)	T (14/16) C (16/14)	Wuling powder + RT	RT	3 w	①⑫	Beijing
Lin Bo 2016	(54.2 ± 1.9)/(53.6 ± 1.8)	100 (50/50)	(3.7 ± 1.3)/(3.6 ± 1.2)	T (24/26) C (25/25)	Wuling powder + RT	Gliquidone	8 w	②⑥⑧⑨	Dalian, Liaoning
Shaoping Zhuo 2016	(56.9 ± 3.5)/(58.2 ± 3.2)	120 (60/60)	(5.8 ± 2.3)/(5.4 ± 2.6)	64/56	Wuling powder + RT	RT	8 w	③④⑤⑦⑫	Lianping, Guangdong
Renzhi Jing 2017	(52.64 ± 8.81)/(51.29 ± 8.26)	68 (34/34)	(10.79 ± 4.99)/(11.02 ± 4.92)	T (20/14) C (18/16)	Wuling powder + RT	RT	11 w	③④⑤⑥⑨⑩⑪⑫⑬	Chengdu, Sichuan
Xiaobo Hao 2017	(54.5 ± 5.5)/(53.5 ± 5.0)	98 (49/49)	(15.0 ± 4.0)/(14.5 ± 3.6)	T (26/23) C (27/22)	Wuling powder + RT	RT	12 w	②③⑥⑧⑨⑩⑪⑫	Zhoukou, Henan
Jing Chen 2017	(54.92 ± 5.25)/(53.65 ± 5.63)	150 (75/75)	(5.39 ± 0.58)/(5.52 ± 0.62)	T (54/21) C (51/24)	Wuling powder + RT	Gliquidone	8 w	⑫	Hebi, Henan
Qiyao Xin 2017	(50.57 ± 15.17)/(52.57 ± 15.77)	60 (30/30)	(12.25 ± 3.77)/(13.17 ± 2.12)	T (16/14) C (18/12)	Wuling powder + RT	RT	2 w	②③④⑥⑫	Guangdong, Guangzhou
Yu Wang 2018	(69.21 ± 1.30)/(69.58 ± 1.65)	90 (45/45)	NR	T (28/17) C (29/16)	Wuling powder + RT	RT	3 w	①⑫	Zunhua, Hebei
Yuanyuan Lai 2019	(64.3 ± 10.5)/(64.5 ± 10.4)	72 (36/36)	NR	T (21/15) C (20/16)	Wuling powder + RT	RT	12 w	⑫	Beijing
Yunyun Zuo 2020	63/62.50	84 (42/42)	6.50/5.50	T (19/17) C (20/18)	Wuling powder + RT	RT	8 w	⑥⑦⑫	Urumqi, Xinjiang
Yimei Li 2020	(35–75)/(35–75)	70 (35/35)	(5–21)/(5–21)	T (26/9) C (20/15)	Wuling powder + RT	RT	8 w	②③④⑥⑦⑨⑩	Jinan, Shandong
Yutian Chen 2020	(54.16 ± 12.57)/(50.89 ± 12.12)	80 (40/40)	(6.32 ± 2.754)/(5.24 ± 2.604)	T (27/13) C (23/17)	Wuling powder + RT	RT	8 w	②③④⑥⑦⑧⑨⑫	Guangdong, Guangzhou
Xiaoting Wen 2020	55.5	40 (20/20)	3∼15	18/22	Wuling powder + RT	RT	4 w	①②③⑫	Qitaihe, Heilongjiang
Zhixiang Jiang 2021	(59.3 ± 2.8)/(58.6 ± 2.8)	86 (43/43)	(7.5 ± 1.7)/(7.2 ± 1.6)	T (26/17) C (28/15)	Wuling powder + RT	RT	4 w	③④⑤⑫	Ezhou, Hubei

T: treatment group; C: control group; F: female; M: male; NR: not reported; W: week; RT: western conventional treatment; ①24 h urine volume; ②24 h urine protein quantification; ③Serum creatinine; ④blood urea nitrogen; ⑤urinary albumin excretion rate; ⑥fasting blood glucose; ⑦glycated hemoglobin; ⑧total cholesterol; ⑨triglyceride; ⑩high-density lipoprotein cholesterol; ⑪low-density lipoprotein cholesterol; ⑫total effective rate; ⑬adverse reactions.

**Table 3:** Subgroup analysis.

	Number of comparisons	Results	*P* value for overall effect	*I* 2	*P* value for subgroup difference
24h urine volume		WMD (95% CI)			
All comparisons	6	335.13 [295.01, 375.26]	<0.00001	59%	

Age					0.01
<60 y	4	360.83 [320.76, 400.91]	<0.00001	15%	
≥60y	2	310.13 [309.15, 311.10]	<0.00001	0%	

Region					0.14
Beijing	2	240.47 [109.29, 371.65]	0.0003	0%	
Other provinces	4	344.68 [299.76, 389.59]	<0.00001	73%	

24h UPQ					
All comparisons	13	−1.30 [−1.82, −0.78]	<0.00001	98%	

Age					0.002
<60 y	12	−1.44 [−2.01, −0.87]	<0.00001	99%	
≥60 y	1	−0.44 [−0.69, −0.19]	0.0005	NA	

Different control treatment					<0.0001
Gliquidone	3	−0.45 [−0.54, −0.35]	<0.00001	0%	
Other treatments	10	−2.15 [−2.95, −1.35]	<0.00001	99%	

Course of treatment					0.05
<8 w	5	−2.63 [−4.16, −1.11]	0.0007	99%	
≥8 w	8	−1.00 [−1.53, −0.47]	0.0002	98%	

Region					<0.00001
Guangdong province	2	−117.95 [−129.88, −106.02]	<0.00001	0%	
Other provinces	11	−0.90 [−1.28, −0.52]	<0.00001	97%	

SCr					
All comparisons	14	−10.17 [−11.13, −9.21]	<0.00001	100%	

Course of treatment					0.11
<8 w	6	−2.44 [−2.99, −1.89]	<0.00001	99%	
≥8 w	8	−11.16 [−21.76, −0.57]	0.04	98%	

Region					0.007
Guangdong province	4	−19.36 [−27.52, −11.20]	<0.00001	90%	
Other provinces	10	−8.12 [−9.10, −7.13]	<0.00001	100%	

BUN					
All comparisons	10	−1.62 [−2.30, −0.93]	<0.00001	93%	

Course of treatment					0.26
<8 w	4	−2.01 [−2.79, −1.23]	<0.00001	81%	
≥8 w	6	−1.40 [−2.12, −0.67]	0.0002	86%	

Region					0.41
Guangdong province	4	−1.32 [−2.16, −0.48]	0.002	83%	
Other provinces	6	−1.86 [−2.83, −0.89]	0.0002	95%	

UAER					
All comparisons	6	−24.73 [−35.46, −13.99]	<0.00001	96%	

Age					0.11
<60 y	5	−22.49 [−34.09, −10.89]	0.0001	97%	
≥60 y	1	−38.41 [−53.97, −22.85]	<0.00001	NA	

Course of treatment					0.93
<8 w	2	−24.55 [−40.91, −8.18]	0.003	99%	
≥8 w	4	−25.73 [−44.46, −7.00]	0.007	89%	

Region					0.02
Guangdong province	2	−32.79 [−35.33, −30.25]	<0.00001	0%	
Other provinces	4	−20.21 [−30.58, −9.84]	0.0001	88%	

FBG					
All comparisons	11	−0.63 [−0.97, −0.30]	0.0002	74%	

Age					0.49
<60 y	9	−0.69 [−1.13, −0.25]	0.002	79%	
≥60 y	2	−0.52 [−0.76, −0.28]	<0.0001	0%	

Different control treatment					0.45
Gliquidone	3	−0.87 [−1.72, −0.03]	0.04	70%	
Other treatments	8	−0.52 [−0.85, −0.20]	0.002	68%	

Course of treatment					0.49
<8 w	1	−0.84 [−1.38, −0.30]	0.002	NA	
≥8 w	10	−0.61 [−0.98, −0.25]	0.001	76%	

Region					0.67
Guangdong province	2	−0.73 [−1.07, −0.39]	<0.0001	0%	
Other provinces	9	−0.61 [−1.04, −0.19]	0.005	78%	

Safety					0.78
No adverse effects	9	−0.62 [−0.96, −0.28]	0.0004	72%	
Adverse effects	2	−0.88 [−2.71, 0.95]	0.34	89%	

HbA1C					
All comparisons	6	−0.11 [−0.30, 0.08]	0.26	55%	

Age					0.56
<60 y	5	−0.08 [−0.34, 0.18]	0.57	64%	
≥60 y	1	−0.17 [−0.35, 0.01]	0.07	NA	

Region					0.01
Guangdong province	2	−0.35 [−0.55, −0.14]	0.0008	0%	
Other provinces	4	0.02 [−0.19, 0.23]	0.87	40%	

TC					
All comparisons	9	−0.63 [−1.23, −0.04]	<0.0001	96%	

Age					0.06
<60 y	8	−0.72 [−1.36, −0.07]	0.03	97%	
≥60 y	1	0.06 [−0.42, 0.54]	0.81	NA	

Different control treatment					0.22
Gliquidone	3	−0.14 [−1.00, 0.73]	0.75	92%	
Other treatments	6	−0.88 [−1.68, −0.08]	0.03	97%	

Course of treatment					0.05
<8 w	3	−1.70 [−3.24, −0.16]	0.03	99%	
≥8 w	6	−0.10 [−0.56, 0.35]	0.65	88%	

Region					0.99
Guangdong province	2	−0.64 [−2.98, 1.69]	0.59	99%	
Other provinces	7	−0.63 [−1.23, −0.02]	0.04	96%	

TG					
All comparisons	11	−0.46 [−0.70, −0.23]	0.0001	96%	

Age					0.08
<60 y	10	−0.50 [−0.74, −0.25]	<0.0001	96%	
≥60 y	1	−0.05 [−0.49, 0.39]	0.82	NA	

Different control treatment					0.11
Gliquidone	3	−0.27 [−0.45, −0.09]	0.003	56%	
Other treatments	8	−0.55 [−0.83, −0.26]	0.0002	95%	

Course of treatment					0.7
<8 w	3	−0.38 [−0.89, 0.13]	0.14	98%	
≥8 w	8	−0.49 [−0.72, −0.25]	<0.0001	86%	

Region					<0.00001
Guangdong province	2	−0.84 [−0.89, −0.79]	<0.00001	0%	
Other provinces	9	−0.36 [−0.55, −0.17]	0.0002	87%	

HDL- C					
All comparisons	5	0.32 [0.03, 0.62]	0.03	97%	

Course of treatment					0.0002
<8 w	1	1.06 [0.72, 1.40]	<0.00001	NA	
≥8 w	4	0.17 [−0.14, 0.48]	0.27	97%	

Region					0.0002
Guangdong province	1	1.06 [0.72, 1.40]	<0.00001	NA	
Other provinces	4	0.17 [−0.14, 0.48]	0.27	97%	

LDL-C					
All comparisons	4	−0.72 [−1.10, −0.34]	0.0002	71%	

Course of treatment					0.02
<8 w	1	−1.17 [−1.57, −0.77]	<0.00001	NA	
≥8 w	3	−0.56 [−0.87, −0.24]	0.0005	40%	

Region					0.02
Guangdong province	1	−1.17 [−1.57, −0.77]	<0.00001	NA	
Other provinces	3	−0.56 [−0.87, −0.24]	0.0005	40%	

Total effective rate					
All comparisons	19	1.40 [1.32, 1.48]	<0.00001	18%	

Age					0.72
<60 y	14	1.39 [1.31, 1.48]	<0.00001	27%	
≥60 y	5	1.43 [1.26, 1.62]	<0.00001	0%	

Different control treatment					0.45
Gliquidone	3	1.35 [1.22, 1.50]	<0.00001	15%	
Other treatments	16	1.42 [1.32, 1.51]	<0.00001	26%	

Course of treatment					0.37
<8 w	10	1.44 [1.32, 1.58]	<0.00001	11%	
≥8 w	9	1.37 [1.27, 1.47]	<0.00001	28%	

Region					0.79
Guangdong province	5	1.41 [1.27, 1.58]	<0.00001	54%	
Other provinces	14	1.39 [1.30, 1.48]	<0.00001	7%	

**Table 4:** Certainty of evidence: Wuling powder compared to control treatment for DN. CI, confidence interval; MD, mean difference; RR, risk ratio.

Certainty assessment							№ of patients		Effect		Certainty	Importance
№ of studies	Study design	Risk of bias	Inconsistency	Indirectness	Imprecision	Other considerations	Wuling powder	Placebo	Relative (95% CI)	Absolute (95% CI)		
*24 h urine volume*												
5	Randomized trials	Serious	Not serious	Not serious	Not serious	None	122	122	—	MD 357.95 higher (322.83 higher to 393.06 higher)	⨁⨁⨁◯ Moderate	CRITICAL

*24 h UPQ*												
13	Randomized trials	Serious	Serious	Not serious	Not serious	None	547	507	—	MD 1.3 lower (1.82 lower to 0.78 lower)	⨁⨁◯◯ Low	CRITICAL

*SCr*												
14	Randomized trials	Serious	Serious	Not serious	Not serious	None	626	584	—	MD 10.17 lower (11.13 lower to 9.21 lower)	⨁⨁◯◯ Low	CRITICAL

*BUN*												
10	Randomized trials	Serious	Serious	Not serious	Not serious	None	504	461	—	MD 1.62 lower (2.3 lower to 0.93 lower)	⨁⨁◯◯ Low	CRITICAL

*UAER*												
6	Randomized trials	Serious	Serious	Not serious	Not serious	None	242	240	—	MD 24.73 lower (35.46 lower to 13.99 lower)	⨁⨁◯◯ Low	CRITICAL

*FBG*												
11	Randomized trials	Serious	Serious	Not serious	Not serious	None	525	488	—	MD 0.63 lower (0.97 lower to 0.3 lower)	⨁⨁◯◯ Low	IMPORTANT

*HbA1C*												
6	Randomized trials	Serious	Serious	Not serious	Not serious	None	232	235	—	MD 0.11 lower (0.3 lower to 0.08 higher)	⨁⨁◯◯ Low	IMPORTANT

*TC*												
9	Randomized trials	Serious	Serious	Not serious	Not serious	None	449	407	—	MD 0.63 lower (1.23 lower to 0.04 lower)	⨁⨁◯◯ Low	IMPORTANT

*TG*												
11	Randomized trials	Serious	Serious	Not serious	Not serious	None	518	476	—	MD 0.46 lower (0.7 lower to 0.23 lower)	⨁⨁◯◯ Low	IMPORTANT

*HDL*												
5	Randomized trials	Serious	Serious	Not serious	Not serious	None	193	191	—	MD 0.32 higher (0.03 higher to 0.62 higher)	⨁⨁◯◯ Low	IMPORTANT

*LDL*												
3	Randomized trials	Serious	Not serious	Not serious	Not serious	None	112	113	—	MD 0.57 lower (0.77 lower to 0.37 lower)	⨁⨁⨁◯ Moderate	IMPORTANT

*Overall effective rate*												
19	Randomized trials	Serious	Not serious	Not serious	Not serious	None	758/849 (89.3%)	515/808 (63.7%)	RR 1.40 (1.32 to 1.48)	255 more per 1,000 (from 204 more to 306 more)	⨁⨁⨁◯Moderate	CRITICAL

## Data Availability

All data relevant to the study are included in the article.

## Abbreviations

TCM: Traditional Chinese medicine
DN: Diabetic nephropathy
CNKI: China knowledge infrastructure
Sino Med: China biomedical CD-ROM
VIP: Vipers database
RCTs: Randomized controlled trials
WMD: Weighted mean differences
CI: Confidence intervals
24 h UPQ: 24 h urine protein quantification
Scr: Serum creatinine
BUN: Blood urea nitrogen
UAER: Urinary albumin excretion rate
FBG: Fasting blood glucose
TC: Total cholesterol
TG: Triglycerides
HDL-C: High-density lipoprotein cholesterol
LDL-C: Low-density lipoprotein cholesterol
TER: Total effective rate
RR: Response ratio
DM: Diabetes mellitus
MD: Mean differences
FEM: Fixed effect model
REM: Random effect model.

## Supplementary Materials

Supplementary Materials Supplementary Table 1: PubMed search strategy. Supplementary Figure 1: subgroup analysis of 24h urine volume (age). Supplementary Figure 2: subgroup analysis of 24h urine volume (region). Supplementary Figure 3: subgroup analysis of 24h urine protein quantification (age). Supplementary Figure 4: subgroup analysis of 24h urine protein quantification (control treatment). Supplementary Figure 5: subgroup analysis of 24h urine protein quantification (course of treatment). Supplementary Figure 6: subgroup analysis of 24h urine protein quantification (region). Supplementary Figure 7: subgroup analysis of serum creatinine (course of treatment). Supplementary Figure 8: subgroup analysis of blood creatinine (region). Supplementary Figure 9: subgroup analysis of blood urea nitrogen (course of treatment). Supplementary Figure 10: subgroup analysis of blood urea nitrogen (region). Supplementary Figure 11: subgroup analysis of urinary albumin excretion rates (age). Supplementary Figure 12: subgroup analysis of urinary albumin excretion rates (course of treatment). Supplementary Figure 13: subgroup analysis of urinary albumin excretion rates (region). Supplementary Figure 14: subgroup analysis of fasting blood glucose (age). Supplementary Figure 15: subgroup analysis of fasting blood glucose (control treatment). Supplementary Figure 16: subgroup analysis of fasting blood glucose (course of treatment). Supplementary Figure 17: subgroup analysis of fasting blood glucose (region). Supplementary Figure 18: subgroup analysis of fasting blood glucose (adverse effects). Supplementary Figure 19: subgroup analysis of glycated hemoglobin (age). Supplementary Figure 20: subgroup analysis of glycated hemoglobin (region). Supplementary Figure 21: subgroup analysis of TC (age). Supplementary Figure 22: subgroup analysis of TC (control treatment). Supplementary Figure 23: subgroup analysis of TC (course of treatment). Supplementary Figure 24: subgroup analysis of TC (region). Supplementary Figure 25: subgroup analysis of TG (age). Supplementary Figure 26: subgroup analysis of TG (control treatment). Supplementary Figure 27: subgroup analysis of TG (course of treatment). Supplementary Figure 28: subgroup analysis of TG (region). Supplementary Figure 29: subgroup analysis of HDL (course of treatment). Supplementary Figure 30: subgroup analysis of HDL (region). Supplementary Figure 31: subgroup analysis of LDL (course of treatment). Supplementary Figure 32: subgroup analysis of LDL (region). Supplementary Figure 33: subgroup analysis of total effective rate (age). Supplementary Figure 34: subgroup analysis of total effective rate (control treatment). Supplementary Figure 35: subgroup analysis of overall effectiveness (course of treatment). Supplementary Figure 36: subgroup analysis of total efficiency (region).
