# Supplementary material for: Efficacy and Safety of Wuling Powder in the Treatment of Patients with Diabetic Nephropathy: A Systematic Review and Meta-Analysis
Source: Evid Based Complement Alternat Med. 2022 Sep 30;2022:1720749. doi: 10.1155/2022/1720749 (PMC9546715; doi:10.1155/2022/1720749)
Supplement: Supplementary Materials — Supplementary Table 1: PubMed search strategy. Supplementary Figure 1: subgroup analysis of 24h urine volume (age). Supplementary Figure 2: subgroup analysis of 24h urine volume (region). Supplementary Figure 3: subgroup analysis of 24h urine protein quantification (age). Supplementary Figure 4: subgroup analysis of 24h urine protein quantification (control treatment). Supplementary Figure 5: subgroup analysis of 24h urine protein quantification (course of treatment). Supplementary Figure 6: subgroup analysis of 24h urine protein quantification (region). Supplementary Figure 7: subgroup analysis of serum creatinine (course of treatment). Supplementary Figure 8: subgroup analysis of blood creatinine (region). Supplementary Figure 9: subgroup analysis of blood urea nitrogen (course of treatment). Supplementary Figure 10: subgroup analysis of blood urea nitrogen (region). Supplementary Figure 11: subgroup analysis of urinary albumin excretion rates (age). Supplementary Figure 12: subgroup analysis of urinary albumin excretion rates (course of treatment). Supplementary Figure 13: subgroup analysis of urinary albumin excretion rates (region). Supplementary Figure 14: subgroup analysis of fasting blood glucose (age). Supplementary Figure 15: subgroup analysis of fasting blood glucose (control treatment). Supplementary Figure 16: subgroup analysis of fasting blood glucose (course of treatment). Supplementary Figure 17: subgroup analysis of fasting blood glucose (region). Supplementary Figure 18: subgroup analysis of fasting blood glucose (adverse effects). Supplementary Figure 19: subgroup analysis of glycated hemoglobin (age). Supplementary Figure 20: subgroup analysis of glycated hemoglobin (region). Supplementary Figure 21: subgroup analysis of TC (age). Supplementary Figure 22: subgroup analysis of TC (control treatment). Supplementary Figure 23: subgroup analysis of TC (course of treatment). Supplementary Figure 24: subgroup analysis of TC (region). Supplementary Figu [file 1720749.f1.zip › Table 1 PubMed search strategy (1).docx]

Table 1: PubMed search strategy.

| Search strategies in PubMed. |
| --- |
| Query |
| " Diabetic Nephropathies "[Mesh] |
| (Nephropathies, Diabetic [Title/Abstract]) OR (Nephropathy, Diabetic [Title/Abstract])) OR (Diabetic Nephropathy [Title/Abstract])) OR (Diabetic Kidney Disease [Title/Abstract])) OR (Kidney Disease, Diabetic[Title/Abstract])) OR (Kidney Diseases, Diabetic[Title/Abstract])) OR (Diabetic Glomerulosclerosis[Title/Abstract])) OR (Glomerulosclerosis, Diabetic[Title/Abstract])) OR (Intracapillary Glomerulosclerosis[Title/Abstract])) OR (Nodular Glomerulosclerosis[Title/Abstract])) OR (Glomerulosclerosis, Nodular[Title/Abstract])) OR (Kimmelstiel-Wilson Syndrome[Title/Abstract])) OR (Kimmelstiel Wilson Syndrome[Title/Abstract])) OR (Syndrome, Kimmelstiel-Wilson[Title/Abstract])) OR (Kimmelstiel-Wilson Disease[Title/Abstract])) OR (Kimmelstiel Wilson Disease[Title/Abstract]) |
| #1 OR #2 |
| (((wuling [Title/Abstract]) OR (Wuling San [Title/Abstract])) OR (Wuling Powder [Title/Abstract]) |
| randomized controlled trial[Publication Type] OR randomized[Title/Abstract] OR placebo[Title/Abstract] |
| #3 AND #4 AND #5 |
