# Supplementary figures and images for: Efficacy and Safety of Wuling Powder in the Treatment of Patients with Diabetic Nephropathy: A Systematic Review and Meta-Analysis
Source: Evid Based Complement Alternat Med. 2022 Sep 30;2022:1720749. doi: 10.1155/2022/1720749 (PMC9546715; doi:10.1155/2022/1720749)

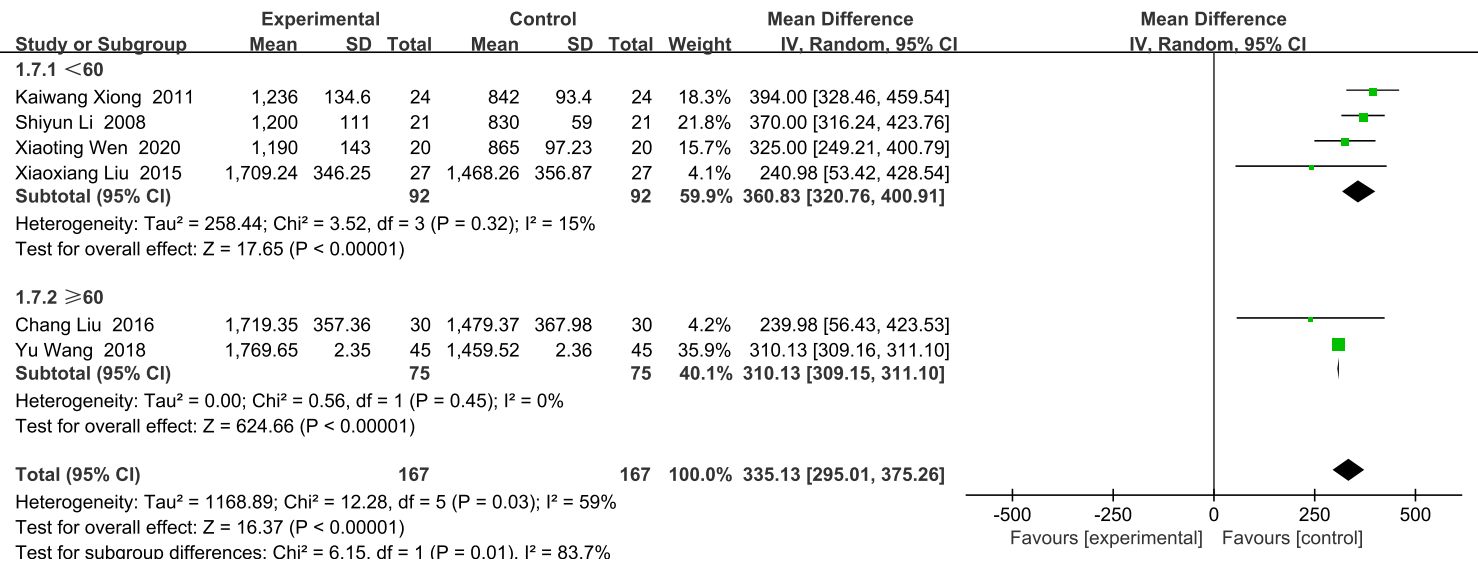

Supplement: Supplementary Materials — Supplementary Table 1: PubMed search strategy. Supplementary Figure 1: subgroup analysis of 24h urine volume (age). Supplementary Figure 2: subgroup analysis of 24h urine volume (region). Supplementary Figure 3: subgroup analysis of 24h urine protein quantification (age). Supplementary Figure 4: subgroup analysis of 24h urine protein quantification (control treatment). Supplementary Figure 5: subgroup analysis of 24h urine protein quantification (course of treatment). Supplementary Figure 6: subgroup analysis of 24h urine protein quantification (region). Supplementary Figure 7: subgroup analysis of serum creatinine (course of treatment). Supplementary Figure 8: subgroup analysis of blood creatinine (region). Supplementary Figure 9: subgroup analysis of blood urea nitrogen (course of treatment). Supplementary Figure 10: subgroup analysis of blood urea nitrogen (region). Supplementary Figure 11: subgroup analysis of urinary albumin excretion rates (age). Supplementary Figure 12: subgroup analysis of urinary albumin excretion rates (course of treatment). Supplementary Figure 13: subgroup analysis of urinary albumin excretion rates (region). Supplementary Figure 14: subgroup analysis of fasting blood glucose (age). Supplementary Figure 15: subgroup analysis of fasting blood glucose (control treatment). Supplementary Figure 16: subgroup analysis of fasting blood glucose (course of treatment). Supplementary Figure 17: subgroup analysis of fasting blood glucose (region). Supplementary Figure 18: subgroup analysis of fasting blood glucose (adverse effects). Supplementary Figure 19: subgroup analysis of glycated hemoglobin (age). Supplementary Figure 20: subgroup analysis of glycated hemoglobin (region). Supplementary Figure 21: subgroup analysis of TC (age). Supplementary Figure 22: subgroup analysis of TC (control treatment). Supplementary Figure 23: subgroup analysis of TC (course of treatment). Supplementary Figure 24: subgroup analysis of TC (region). Supplementary Figu [file 1720749.f1.zip › Fig1 Subgroup analysis of 24h urine volume (age).pdf]

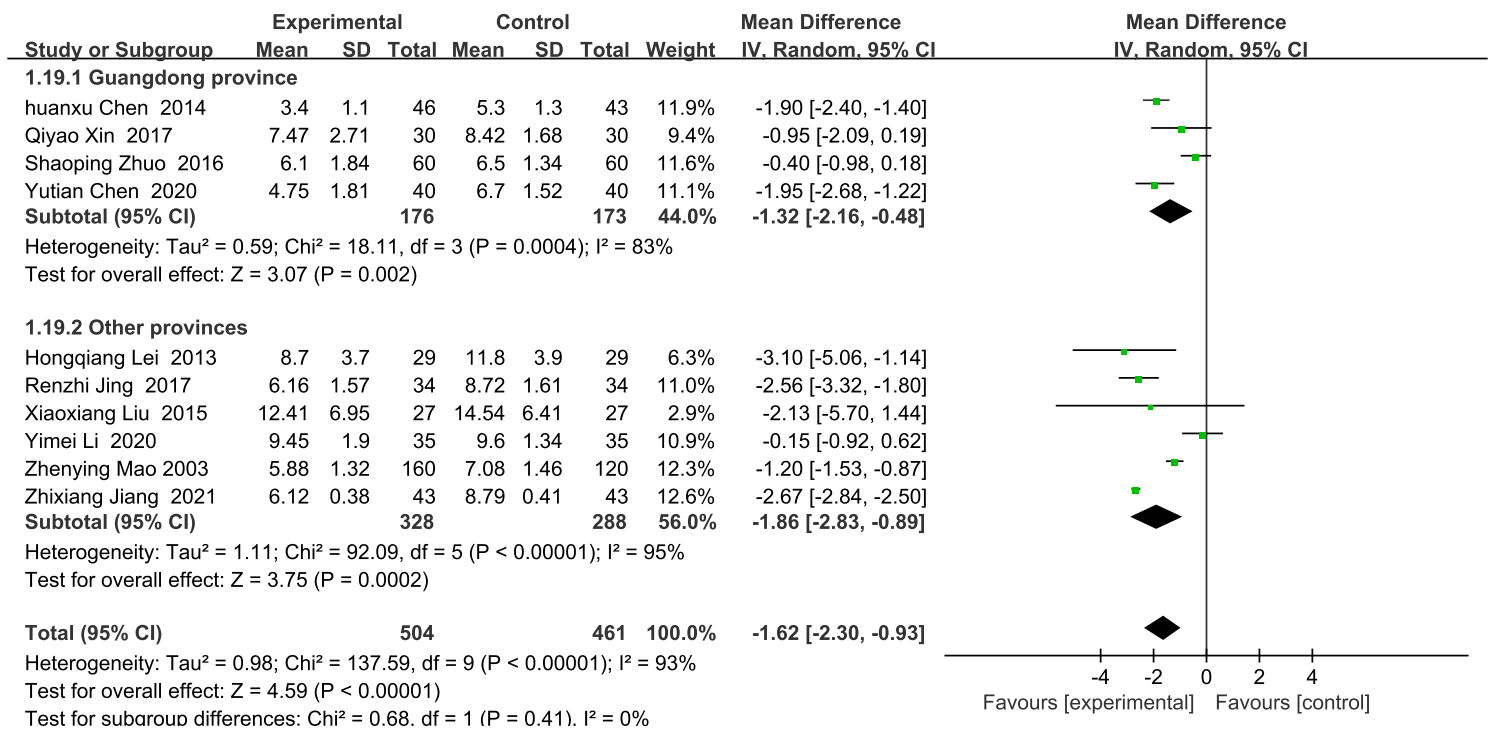

Supplement: Supplementary Materials — Supplementary Table 1: PubMed search strategy. Supplementary Figure 1: subgroup analysis of 24h urine volume (age). Supplementary Figure 2: subgroup analysis of 24h urine volume (region). Supplementary Figure 3: subgroup analysis of 24h urine protein quantification (age). Supplementary Figure 4: subgroup analysis of 24h urine protein quantification (control treatment). Supplementary Figure 5: subgroup analysis of 24h urine protein quantification (course of treatment). Supplementary Figure 6: subgroup analysis of 24h urine protein quantification (region). Supplementary Figure 7: subgroup analysis of serum creatinine (course of treatment). Supplementary Figure 8: subgroup analysis of blood creatinine (region). Supplementary Figure 9: subgroup analysis of blood urea nitrogen (course of treatment). Supplementary Figure 10: subgroup analysis of blood urea nitrogen (region). Supplementary Figure 11: subgroup analysis of urinary albumin excretion rates (age). Supplementary Figure 12: subgroup analysis of urinary albumin excretion rates (course of treatment). Supplementary Figure 13: subgroup analysis of urinary albumin excretion rates (region). Supplementary Figure 14: subgroup analysis of fasting blood glucose (age). Supplementary Figure 15: subgroup analysis of fasting blood glucose (control treatment). Supplementary Figure 16: subgroup analysis of fasting blood glucose (course of treatment). Supplementary Figure 17: subgroup analysis of fasting blood glucose (region). Supplementary Figure 18: subgroup analysis of fasting blood glucose (adverse effects). Supplementary Figure 19: subgroup analysis of glycated hemoglobin (age). Supplementary Figure 20: subgroup analysis of glycated hemoglobin (region). Supplementary Figure 21: subgroup analysis of TC (age). Supplementary Figure 22: subgroup analysis of TC (control treatment). Supplementary Figure 23: subgroup analysis of TC (course of treatment). Supplementary Figure 24: subgroup analysis of TC (region). Supplementary Figu [file 1720749.f1.zip › Fig10 Subgroup analysis of blood urea nitrogen (region).pdf]

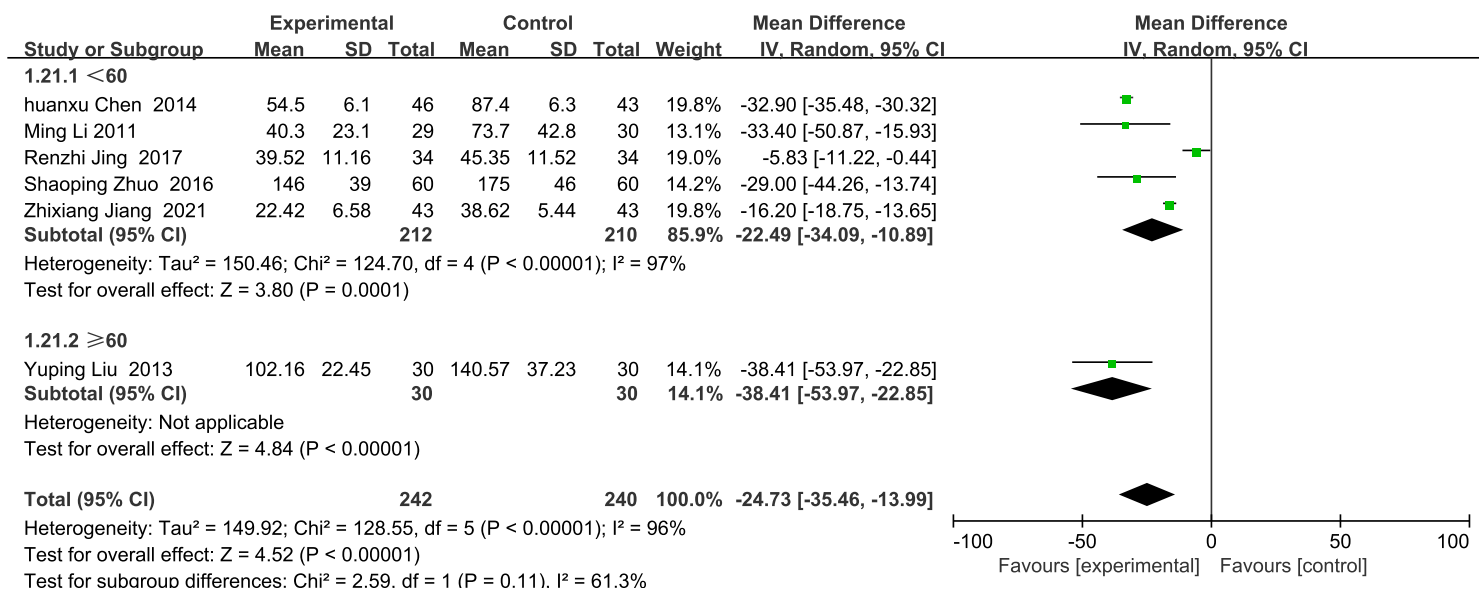

Supplement: Supplementary Materials — Supplementary Table 1: PubMed search strategy. Supplementary Figure 1: subgroup analysis of 24h urine volume (age). Supplementary Figure 2: subgroup analysis of 24h urine volume (region). Supplementary Figure 3: subgroup analysis of 24h urine protein quantification (age). Supplementary Figure 4: subgroup analysis of 24h urine protein quantification (control treatment). Supplementary Figure 5: subgroup analysis of 24h urine protein quantification (course of treatment). Supplementary Figure 6: subgroup analysis of 24h urine protein quantification (region). Supplementary Figure 7: subgroup analysis of serum creatinine (course of treatment). Supplementary Figure 8: subgroup analysis of blood creatinine (region). Supplementary Figure 9: subgroup analysis of blood urea nitrogen (course of treatment). Supplementary Figure 10: subgroup analysis of blood urea nitrogen (region). Supplementary Figure 11: subgroup analysis of urinary albumin excretion rates (age). Supplementary Figure 12: subgroup analysis of urinary albumin excretion rates (course of treatment). Supplementary Figure 13: subgroup analysis of urinary albumin excretion rates (region). Supplementary Figure 14: subgroup analysis of fasting blood glucose (age). Supplementary Figure 15: subgroup analysis of fasting blood glucose (control treatment). Supplementary Figure 16: subgroup analysis of fasting blood glucose (course of treatment). Supplementary Figure 17: subgroup analysis of fasting blood glucose (region). Supplementary Figure 18: subgroup analysis of fasting blood glucose (adverse effects). Supplementary Figure 19: subgroup analysis of glycated hemoglobin (age). Supplementary Figure 20: subgroup analysis of glycated hemoglobin (region). Supplementary Figure 21: subgroup analysis of TC (age). Supplementary Figure 22: subgroup analysis of TC (control treatment). Supplementary Figure 23: subgroup analysis of TC (course of treatment). Supplementary Figure 24: subgroup analysis of TC (region). Supplementary Figu [file 1720749.f1.zip › Fig11 Subgroup analysis of urinary albumin excretion rates (age).pdf]

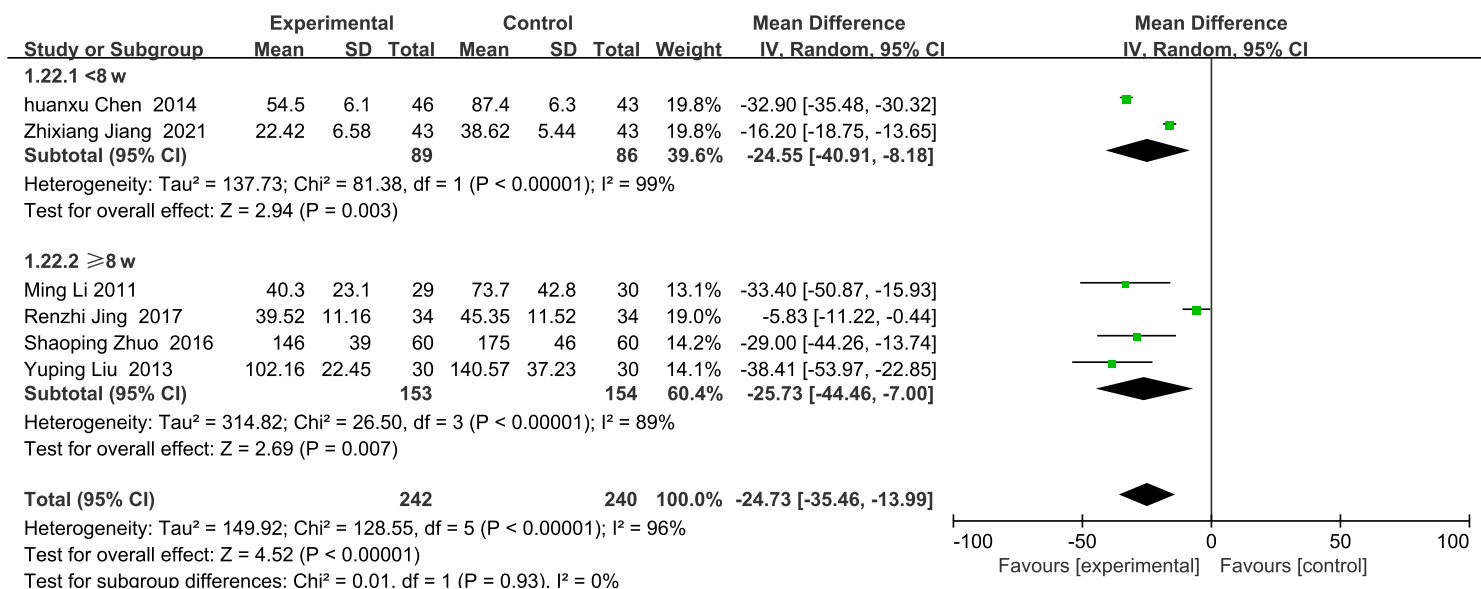

Supplement: Supplementary Materials — Supplementary Table 1: PubMed search strategy. Supplementary Figure 1: subgroup analysis of 24h urine volume (age). Supplementary Figure 2: subgroup analysis of 24h urine volume (region). Supplementary Figure 3: subgroup analysis of 24h urine protein quantification (age). Supplementary Figure 4: subgroup analysis of 24h urine protein quantification (control treatment). Supplementary Figure 5: subgroup analysis of 24h urine protein quantification (course of treatment). Supplementary Figure 6: subgroup analysis of 24h urine protein quantification (region). Supplementary Figure 7: subgroup analysis of serum creatinine (course of treatment). Supplementary Figure 8: subgroup analysis of blood creatinine (region). Supplementary Figure 9: subgroup analysis of blood urea nitrogen (course of treatment). Supplementary Figure 10: subgroup analysis of blood urea nitrogen (region). Supplementary Figure 11: subgroup analysis of urinary albumin excretion rates (age). Supplementary Figure 12: subgroup analysis of urinary albumin excretion rates (course of treatment). Supplementary Figure 13: subgroup analysis of urinary albumin excretion rates (region). Supplementary Figure 14: subgroup analysis of fasting blood glucose (age). Supplementary Figure 15: subgroup analysis of fasting blood glucose (control treatment). Supplementary Figure 16: subgroup analysis of fasting blood glucose (course of treatment). Supplementary Figure 17: subgroup analysis of fasting blood glucose (region). Supplementary Figure 18: subgroup analysis of fasting blood glucose (adverse effects). Supplementary Figure 19: subgroup analysis of glycated hemoglobin (age). Supplementary Figure 20: subgroup analysis of glycated hemoglobin (region). Supplementary Figure 21: subgroup analysis of TC (age). Supplementary Figure 22: subgroup analysis of TC (control treatment). Supplementary Figure 23: subgroup analysis of TC (course of treatment). Supplementary Figure 24: subgroup analysis of TC (region). Supplementary Figu [file 1720749.f1.zip › Fig12 Subgroup analysis of urinary albumin excretion rates (course of treatment).pdf]

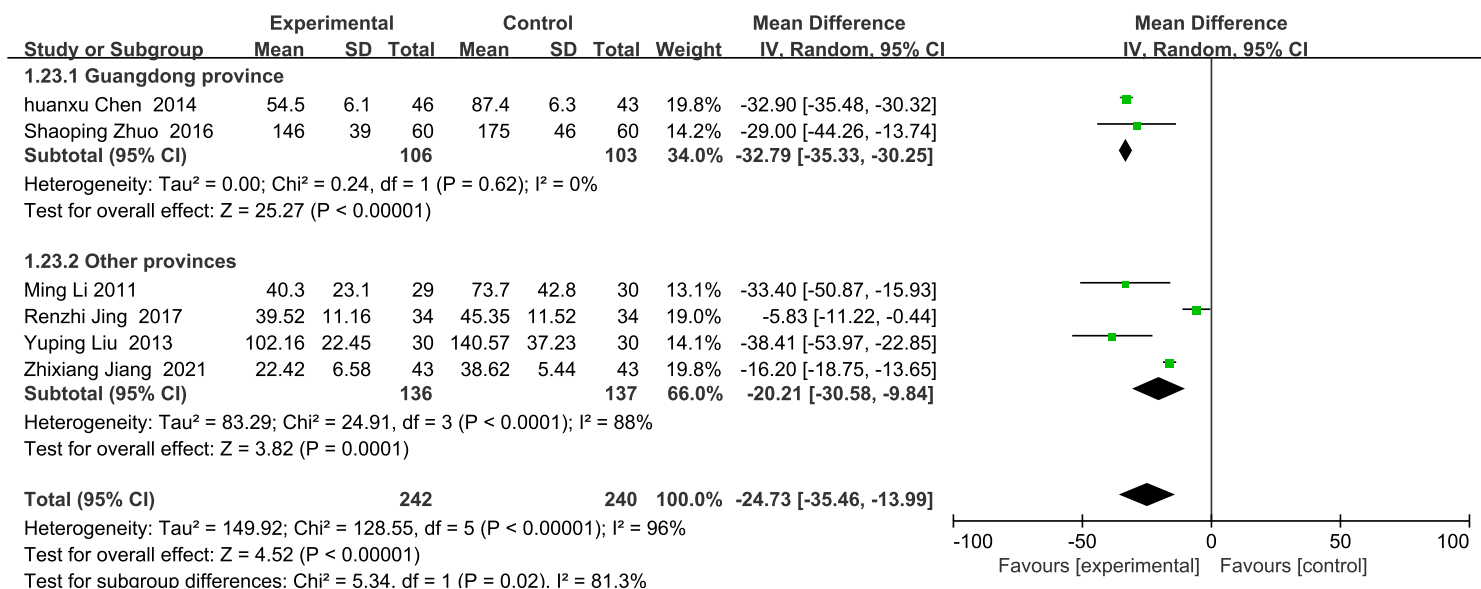

Supplement: Supplementary Materials — Supplementary Table 1: PubMed search strategy. Supplementary Figure 1: subgroup analysis of 24h urine volume (age). Supplementary Figure 2: subgroup analysis of 24h urine volume (region). Supplementary Figure 3: subgroup analysis of 24h urine protein quantification (age). Supplementary Figure 4: subgroup analysis of 24h urine protein quantification (control treatment). Supplementary Figure 5: subgroup analysis of 24h urine protein quantification (course of treatment). Supplementary Figure 6: subgroup analysis of 24h urine protein quantification (region). Supplementary Figure 7: subgroup analysis of serum creatinine (course of treatment). Supplementary Figure 8: subgroup analysis of blood creatinine (region). Supplementary Figure 9: subgroup analysis of blood urea nitrogen (course of treatment). Supplementary Figure 10: subgroup analysis of blood urea nitrogen (region). Supplementary Figure 11: subgroup analysis of urinary albumin excretion rates (age). Supplementary Figure 12: subgroup analysis of urinary albumin excretion rates (course of treatment). Supplementary Figure 13: subgroup analysis of urinary albumin excretion rates (region). Supplementary Figure 14: subgroup analysis of fasting blood glucose (age). Supplementary Figure 15: subgroup analysis of fasting blood glucose (control treatment). Supplementary Figure 16: subgroup analysis of fasting blood glucose (course of treatment). Supplementary Figure 17: subgroup analysis of fasting blood glucose (region). Supplementary Figure 18: subgroup analysis of fasting blood glucose (adverse effects). Supplementary Figure 19: subgroup analysis of glycated hemoglobin (age). Supplementary Figure 20: subgroup analysis of glycated hemoglobin (region). Supplementary Figure 21: subgroup analysis of TC (age). Supplementary Figure 22: subgroup analysis of TC (control treatment). Supplementary Figure 23: subgroup analysis of TC (course of treatment). Supplementary Figure 24: subgroup analysis of TC (region). Supplementary Figu [file 1720749.f1.zip › Fig13 Subgroup analysis of urinary albumin excretion rates (region).pdf]

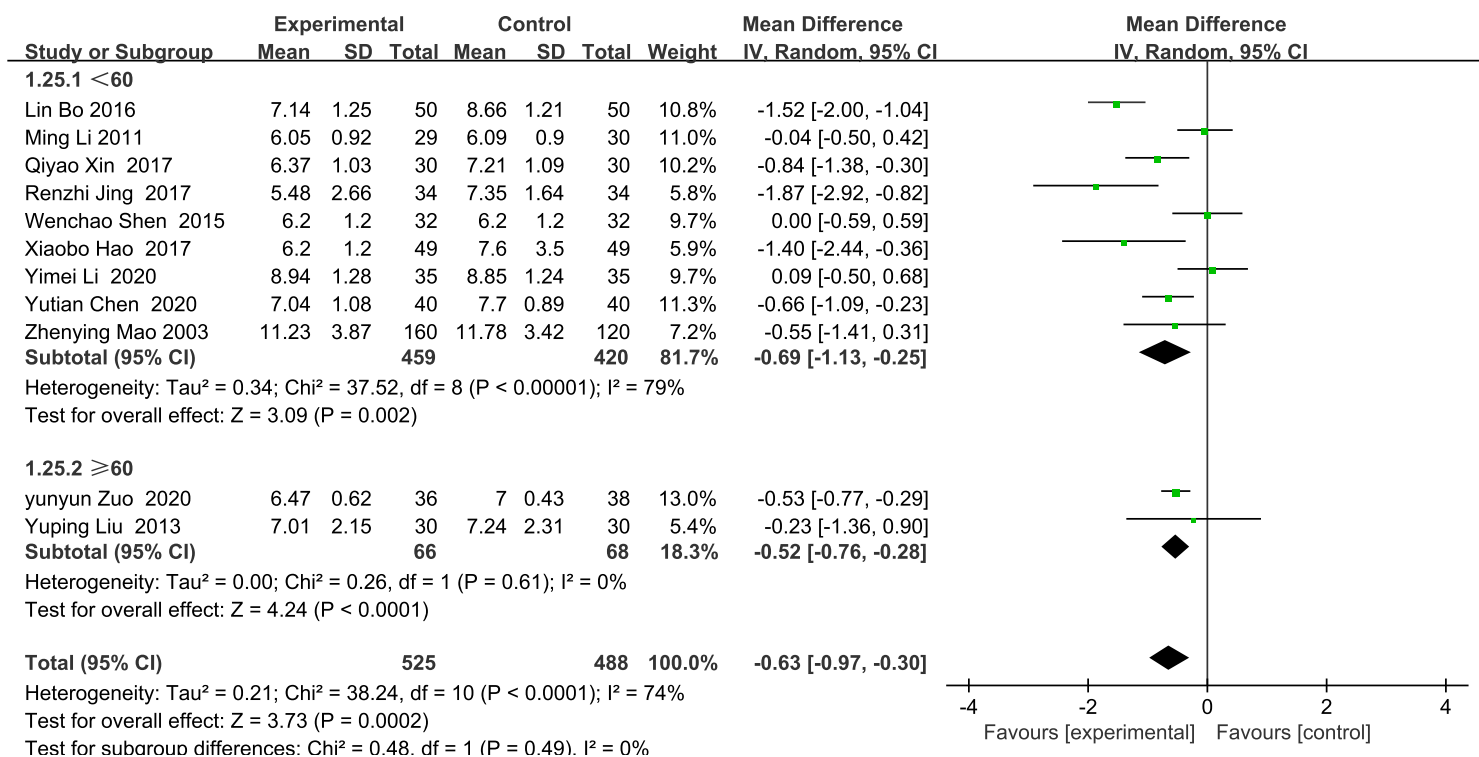

Supplement: Supplementary Materials — Supplementary Table 1: PubMed search strategy. Supplementary Figure 1: subgroup analysis of 24h urine volume (age). Supplementary Figure 2: subgroup analysis of 24h urine volume (region). Supplementary Figure 3: subgroup analysis of 24h urine protein quantification (age). Supplementary Figure 4: subgroup analysis of 24h urine protein quantification (control treatment). Supplementary Figure 5: subgroup analysis of 24h urine protein quantification (course of treatment). Supplementary Figure 6: subgroup analysis of 24h urine protein quantification (region). Supplementary Figure 7: subgroup analysis of serum creatinine (course of treatment). Supplementary Figure 8: subgroup analysis of blood creatinine (region). Supplementary Figure 9: subgroup analysis of blood urea nitrogen (course of treatment). Supplementary Figure 10: subgroup analysis of blood urea nitrogen (region). Supplementary Figure 11: subgroup analysis of urinary albumin excretion rates (age). Supplementary Figure 12: subgroup analysis of urinary albumin excretion rates (course of treatment). Supplementary Figure 13: subgroup analysis of urinary albumin excretion rates (region). Supplementary Figure 14: subgroup analysis of fasting blood glucose (age). Supplementary Figure 15: subgroup analysis of fasting blood glucose (control treatment). Supplementary Figure 16: subgroup analysis of fasting blood glucose (course of treatment). Supplementary Figure 17: subgroup analysis of fasting blood glucose (region). Supplementary Figure 18: subgroup analysis of fasting blood glucose (adverse effects). Supplementary Figure 19: subgroup analysis of glycated hemoglobin (age). Supplementary Figure 20: subgroup analysis of glycated hemoglobin (region). Supplementary Figure 21: subgroup analysis of TC (age). Supplementary Figure 22: subgroup analysis of TC (control treatment). Supplementary Figure 23: subgroup analysis of TC (course of treatment). Supplementary Figure 24: subgroup analysis of TC (region). Supplementary Figu [file 1720749.f1.zip › Fig14 Subgroup analysis of fasting blood glucose (age).pdf]

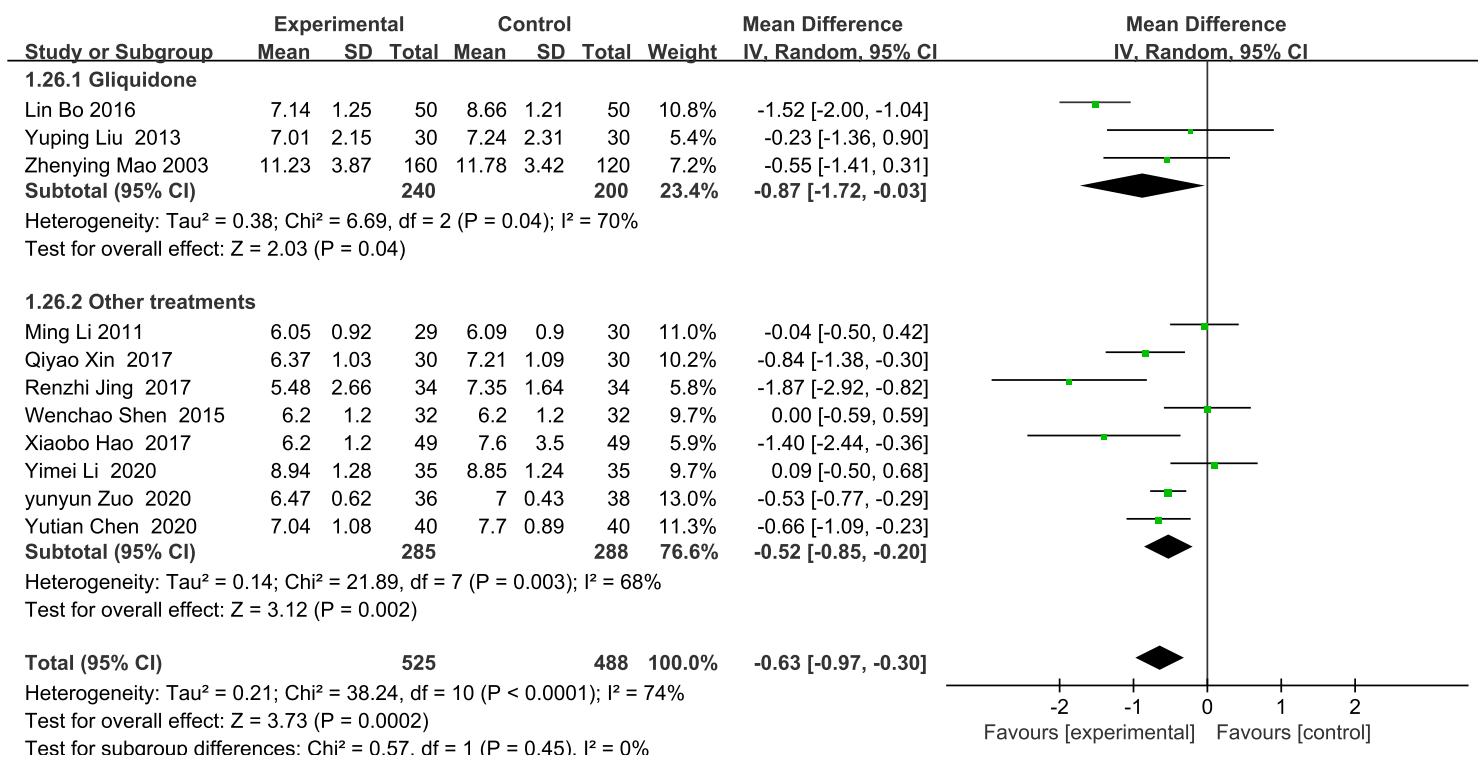

Supplement: Supplementary Materials — Supplementary Table 1: PubMed search strategy. Supplementary Figure 1: subgroup analysis of 24h urine volume (age). Supplementary Figure 2: subgroup analysis of 24h urine volume (region). Supplementary Figure 3: subgroup analysis of 24h urine protein quantification (age). Supplementary Figure 4: subgroup analysis of 24h urine protein quantification (control treatment). Supplementary Figure 5: subgroup analysis of 24h urine protein quantification (course of treatment). Supplementary Figure 6: subgroup analysis of 24h urine protein quantification (region). Supplementary Figure 7: subgroup analysis of serum creatinine (course of treatment). Supplementary Figure 8: subgroup analysis of blood creatinine (region). Supplementary Figure 9: subgroup analysis of blood urea nitrogen (course of treatment). Supplementary Figure 10: subgroup analysis of blood urea nitrogen (region). Supplementary Figure 11: subgroup analysis of urinary albumin excretion rates (age). Supplementary Figure 12: subgroup analysis of urinary albumin excretion rates (course of treatment). Supplementary Figure 13: subgroup analysis of urinary albumin excretion rates (region). Supplementary Figure 14: subgroup analysis of fasting blood glucose (age). Supplementary Figure 15: subgroup analysis of fasting blood glucose (control treatment). Supplementary Figure 16: subgroup analysis of fasting blood glucose (course of treatment). Supplementary Figure 17: subgroup analysis of fasting blood glucose (region). Supplementary Figure 18: subgroup analysis of fasting blood glucose (adverse effects). Supplementary Figure 19: subgroup analysis of glycated hemoglobin (age). Supplementary Figure 20: subgroup analysis of glycated hemoglobin (region). Supplementary Figure 21: subgroup analysis of TC (age). Supplementary Figure 22: subgroup analysis of TC (control treatment). Supplementary Figure 23: subgroup analysis of TC (course of treatment). Supplementary Figure 24: subgroup analysis of TC (region). Supplementary Figu [file 1720749.f1.zip › Fig15 Subgroup analysis of fasting blood glucose (control treatment).pdf]

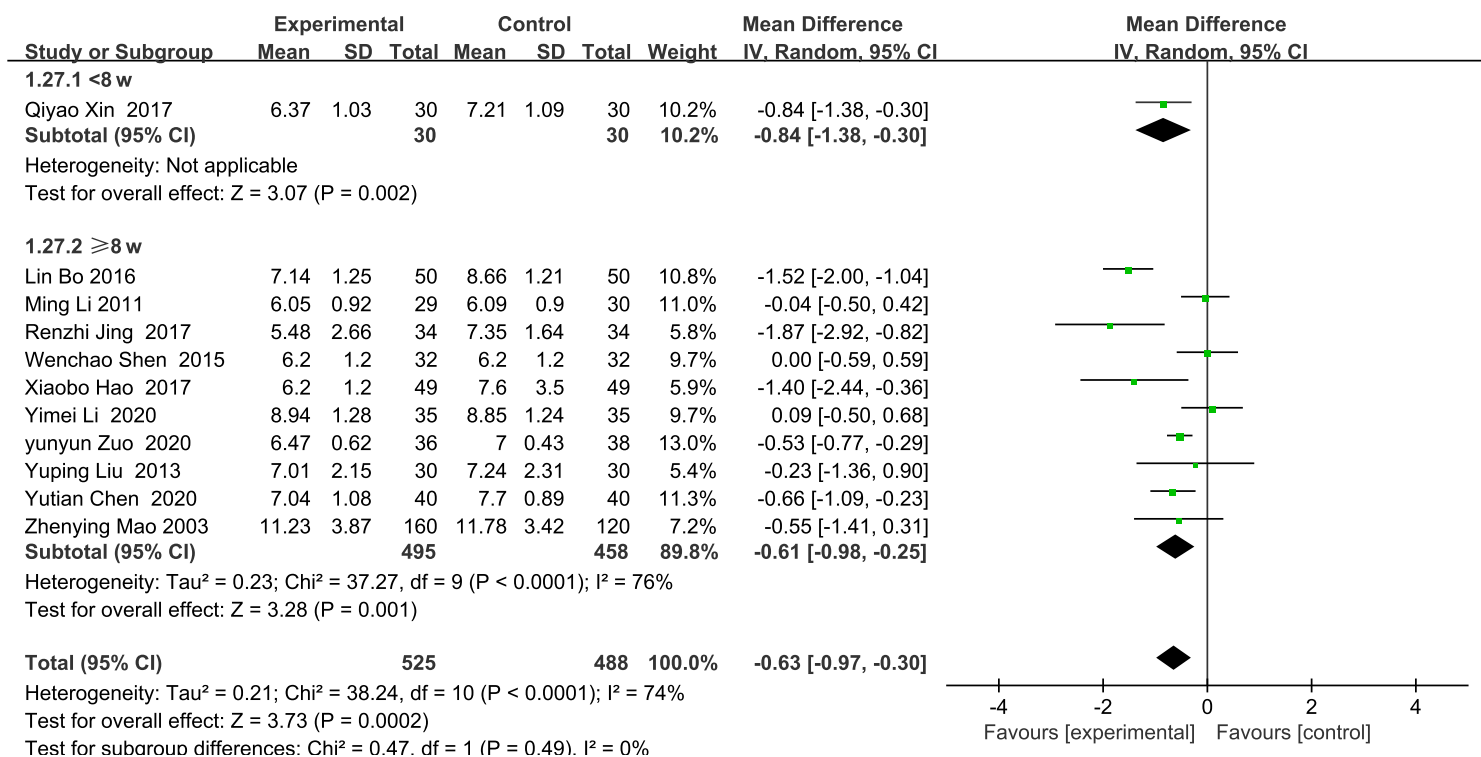

Supplement: Supplementary Materials — Supplementary Table 1: PubMed search strategy. Supplementary Figure 1: subgroup analysis of 24h urine volume (age). Supplementary Figure 2: subgroup analysis of 24h urine volume (region). Supplementary Figure 3: subgroup analysis of 24h urine protein quantification (age). Supplementary Figure 4: subgroup analysis of 24h urine protein quantification (control treatment). Supplementary Figure 5: subgroup analysis of 24h urine protein quantification (course of treatment). Supplementary Figure 6: subgroup analysis of 24h urine protein quantification (region). Supplementary Figure 7: subgroup analysis of serum creatinine (course of treatment). Supplementary Figure 8: subgroup analysis of blood creatinine (region). Supplementary Figure 9: subgroup analysis of blood urea nitrogen (course of treatment). Supplementary Figure 10: subgroup analysis of blood urea nitrogen (region). Supplementary Figure 11: subgroup analysis of urinary albumin excretion rates (age). Supplementary Figure 12: subgroup analysis of urinary albumin excretion rates (course of treatment). Supplementary Figure 13: subgroup analysis of urinary albumin excretion rates (region). Supplementary Figure 14: subgroup analysis of fasting blood glucose (age). Supplementary Figure 15: subgroup analysis of fasting blood glucose (control treatment). Supplementary Figure 16: subgroup analysis of fasting blood glucose (course of treatment). Supplementary Figure 17: subgroup analysis of fasting blood glucose (region). Supplementary Figure 18: subgroup analysis of fasting blood glucose (adverse effects). Supplementary Figure 19: subgroup analysis of glycated hemoglobin (age). Supplementary Figure 20: subgroup analysis of glycated hemoglobin (region). Supplementary Figure 21: subgroup analysis of TC (age). Supplementary Figure 22: subgroup analysis of TC (control treatment). Supplementary Figure 23: subgroup analysis of TC (course of treatment). Supplementary Figure 24: subgroup analysis of TC (region). Supplementary Figu [file 1720749.f1.zip › Fig16 Subgroup analysis of fasting blood glucose (course of treatment).pdf]

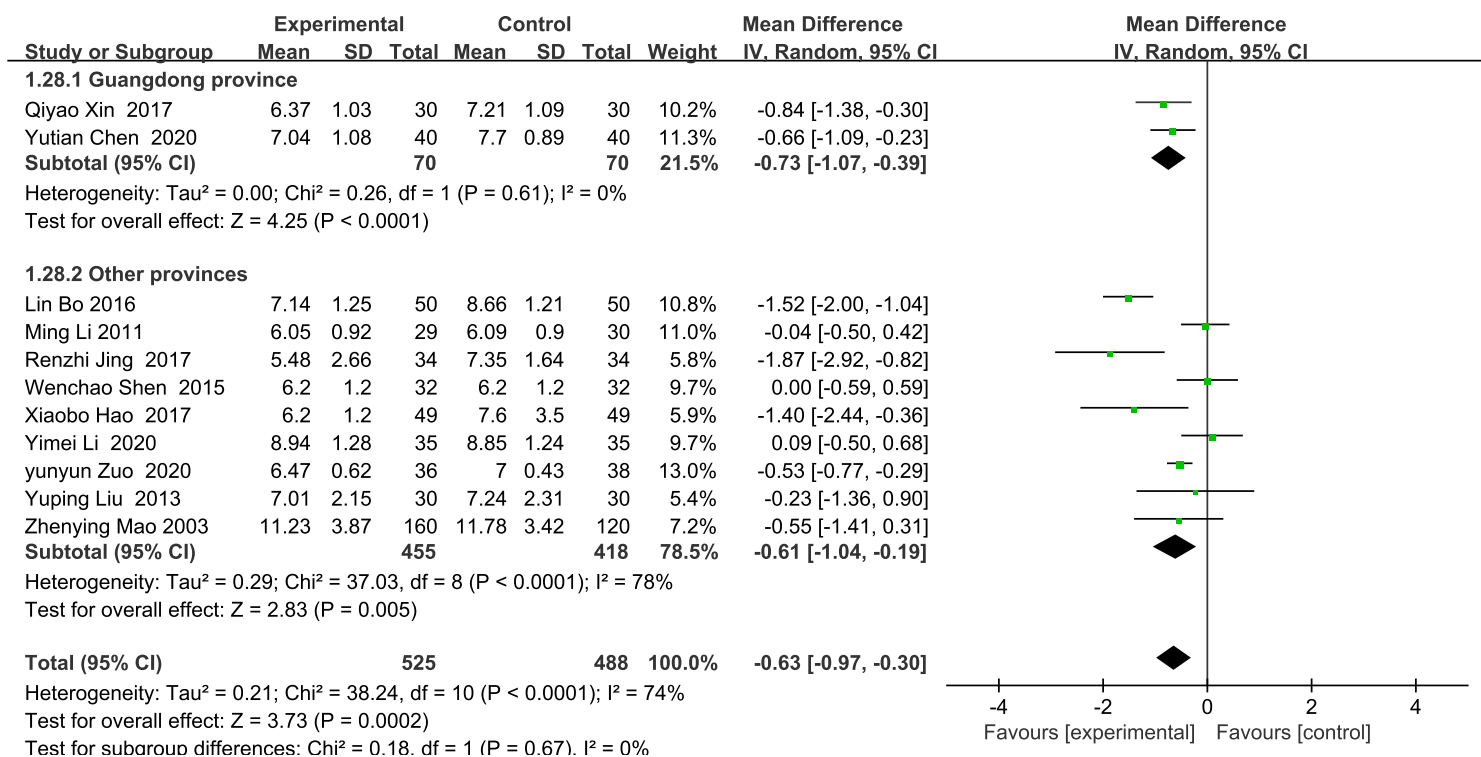

Supplement: Supplementary Materials — Supplementary Table 1: PubMed search strategy. Supplementary Figure 1: subgroup analysis of 24h urine volume (age). Supplementary Figure 2: subgroup analysis of 24h urine volume (region). Supplementary Figure 3: subgroup analysis of 24h urine protein quantification (age). Supplementary Figure 4: subgroup analysis of 24h urine protein quantification (control treatment). Supplementary Figure 5: subgroup analysis of 24h urine protein quantification (course of treatment). Supplementary Figure 6: subgroup analysis of 24h urine protein quantification (region). Supplementary Figure 7: subgroup analysis of serum creatinine (course of treatment). Supplementary Figure 8: subgroup analysis of blood creatinine (region). Supplementary Figure 9: subgroup analysis of blood urea nitrogen (course of treatment). Supplementary Figure 10: subgroup analysis of blood urea nitrogen (region). Supplementary Figure 11: subgroup analysis of urinary albumin excretion rates (age). Supplementary Figure 12: subgroup analysis of urinary albumin excretion rates (course of treatment). Supplementary Figure 13: subgroup analysis of urinary albumin excretion rates (region). Supplementary Figure 14: subgroup analysis of fasting blood glucose (age). Supplementary Figure 15: subgroup analysis of fasting blood glucose (control treatment). Supplementary Figure 16: subgroup analysis of fasting blood glucose (course of treatment). Supplementary Figure 17: subgroup analysis of fasting blood glucose (region). Supplementary Figure 18: subgroup analysis of fasting blood glucose (adverse effects). Supplementary Figure 19: subgroup analysis of glycated hemoglobin (age). Supplementary Figure 20: subgroup analysis of glycated hemoglobin (region). Supplementary Figure 21: subgroup analysis of TC (age). Supplementary Figure 22: subgroup analysis of TC (control treatment). Supplementary Figure 23: subgroup analysis of TC (course of treatment). Supplementary Figure 24: subgroup analysis of TC (region). Supplementary Figu [file 1720749.f1.zip › Fig17 Subgroup analysis of fasting blood glucose (region).pdf]

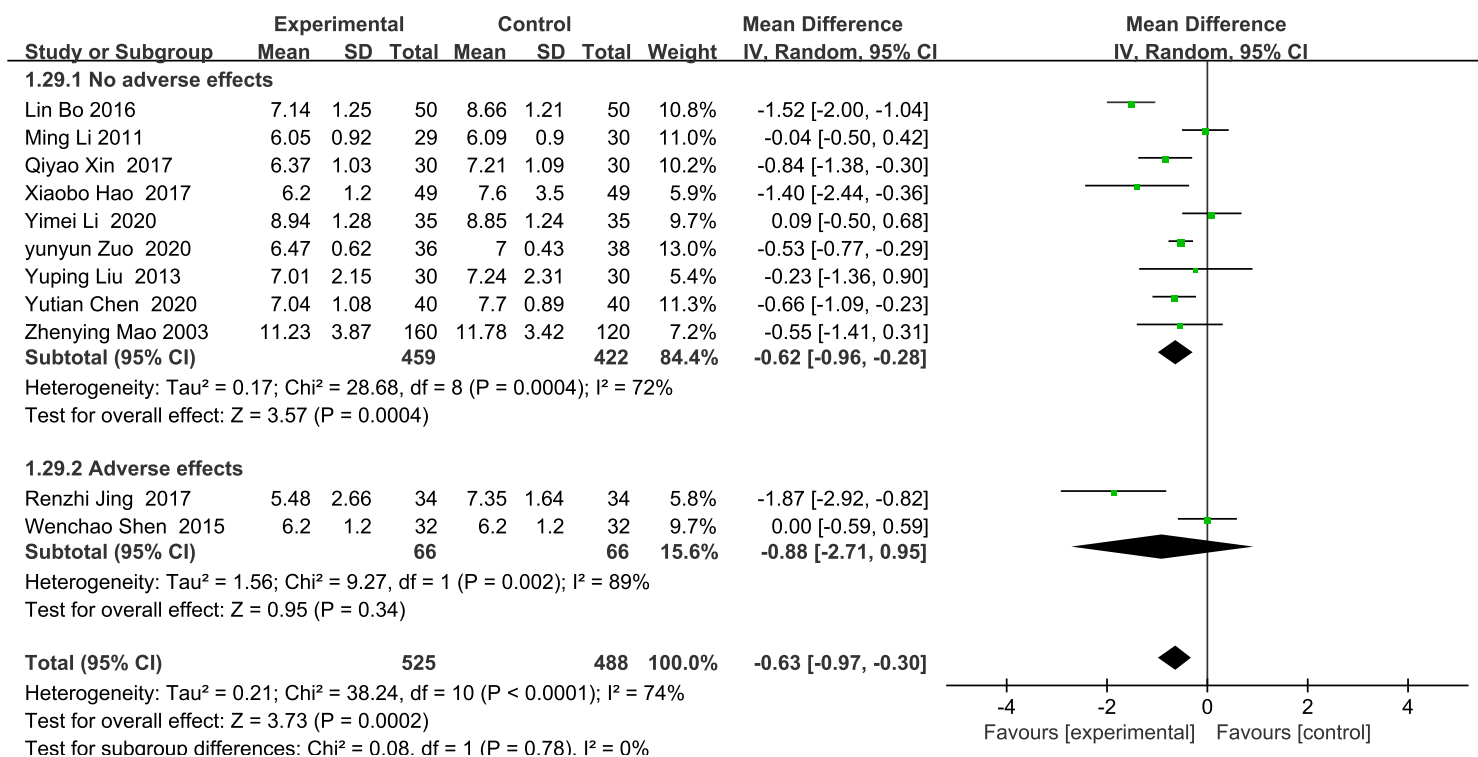

Supplement: Supplementary Materials — Supplementary Table 1: PubMed search strategy. Supplementary Figure 1: subgroup analysis of 24h urine volume (age). Supplementary Figure 2: subgroup analysis of 24h urine volume (region). Supplementary Figure 3: subgroup analysis of 24h urine protein quantification (age). Supplementary Figure 4: subgroup analysis of 24h urine protein quantification (control treatment). Supplementary Figure 5: subgroup analysis of 24h urine protein quantification (course of treatment). Supplementary Figure 6: subgroup analysis of 24h urine protein quantification (region). Supplementary Figure 7: subgroup analysis of serum creatinine (course of treatment). Supplementary Figure 8: subgroup analysis of blood creatinine (region). Supplementary Figure 9: subgroup analysis of blood urea nitrogen (course of treatment). Supplementary Figure 10: subgroup analysis of blood urea nitrogen (region). Supplementary Figure 11: subgroup analysis of urinary albumin excretion rates (age). Supplementary Figure 12: subgroup analysis of urinary albumin excretion rates (course of treatment). Supplementary Figure 13: subgroup analysis of urinary albumin excretion rates (region). Supplementary Figure 14: subgroup analysis of fasting blood glucose (age). Supplementary Figure 15: subgroup analysis of fasting blood glucose (control treatment). Supplementary Figure 16: subgroup analysis of fasting blood glucose (course of treatment). Supplementary Figure 17: subgroup analysis of fasting blood glucose (region). Supplementary Figure 18: subgroup analysis of fasting blood glucose (adverse effects). Supplementary Figure 19: subgroup analysis of glycated hemoglobin (age). Supplementary Figure 20: subgroup analysis of glycated hemoglobin (region). Supplementary Figure 21: subgroup analysis of TC (age). Supplementary Figure 22: subgroup analysis of TC (control treatment). Supplementary Figure 23: subgroup analysis of TC (course of treatment). Supplementary Figure 24: subgroup analysis of TC (region). Supplementary Figu [file 1720749.f1.zip › FIg18 Subgroup analysis of fasting blood glucose (adverse effects).pdf]

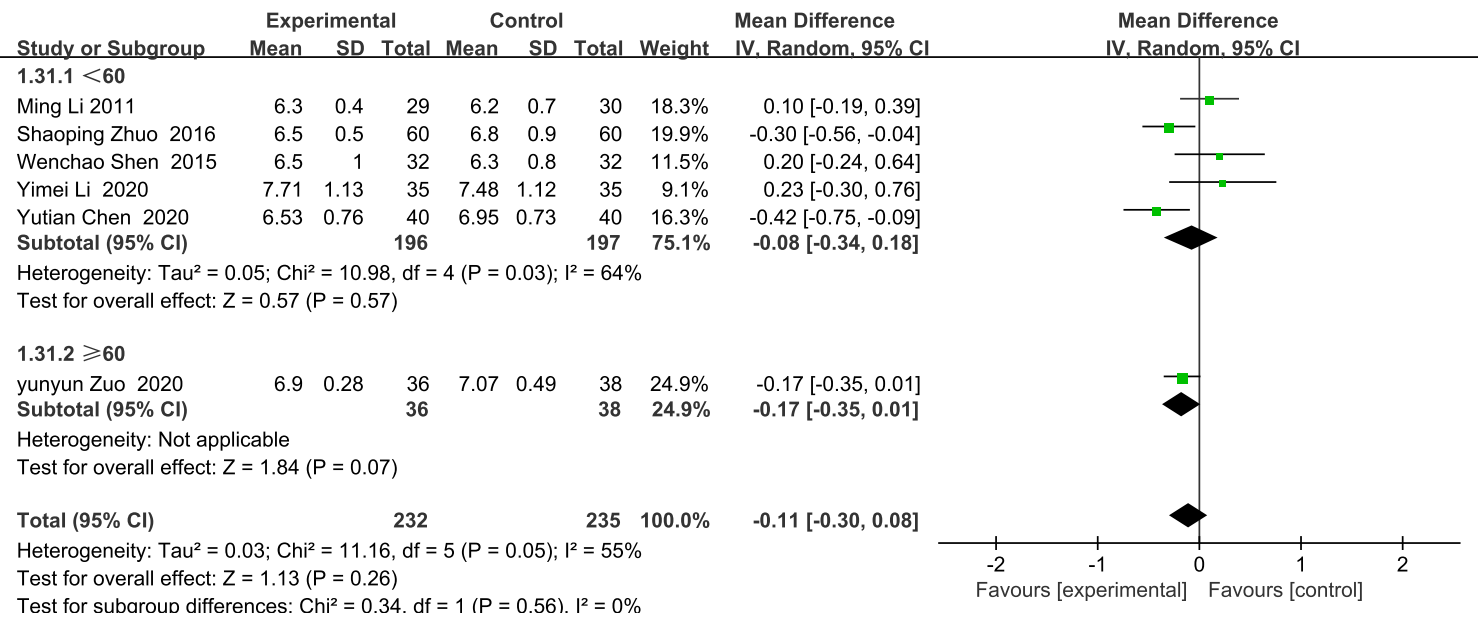

Supplement: Supplementary Materials — Supplementary Table 1: PubMed search strategy. Supplementary Figure 1: subgroup analysis of 24h urine volume (age). Supplementary Figure 2: subgroup analysis of 24h urine volume (region). Supplementary Figure 3: subgroup analysis of 24h urine protein quantification (age). Supplementary Figure 4: subgroup analysis of 24h urine protein quantification (control treatment). Supplementary Figure 5: subgroup analysis of 24h urine protein quantification (course of treatment). Supplementary Figure 6: subgroup analysis of 24h urine protein quantification (region). Supplementary Figure 7: subgroup analysis of serum creatinine (course of treatment). Supplementary Figure 8: subgroup analysis of blood creatinine (region). Supplementary Figure 9: subgroup analysis of blood urea nitrogen (course of treatment). Supplementary Figure 10: subgroup analysis of blood urea nitrogen (region). Supplementary Figure 11: subgroup analysis of urinary albumin excretion rates (age). Supplementary Figure 12: subgroup analysis of urinary albumin excretion rates (course of treatment). Supplementary Figure 13: subgroup analysis of urinary albumin excretion rates (region). Supplementary Figure 14: subgroup analysis of fasting blood glucose (age). Supplementary Figure 15: subgroup analysis of fasting blood glucose (control treatment). Supplementary Figure 16: subgroup analysis of fasting blood glucose (course of treatment). Supplementary Figure 17: subgroup analysis of fasting blood glucose (region). Supplementary Figure 18: subgroup analysis of fasting blood glucose (adverse effects). Supplementary Figure 19: subgroup analysis of glycated hemoglobin (age). Supplementary Figure 20: subgroup analysis of glycated hemoglobin (region). Supplementary Figure 21: subgroup analysis of TC (age). Supplementary Figure 22: subgroup analysis of TC (control treatment). Supplementary Figure 23: subgroup analysis of TC (course of treatment). Supplementary Figure 24: subgroup analysis of TC (region). Supplementary Figu [file 1720749.f1.zip › Fig19 Subgroup analysis of glycated haemoglobin (age).pdf]

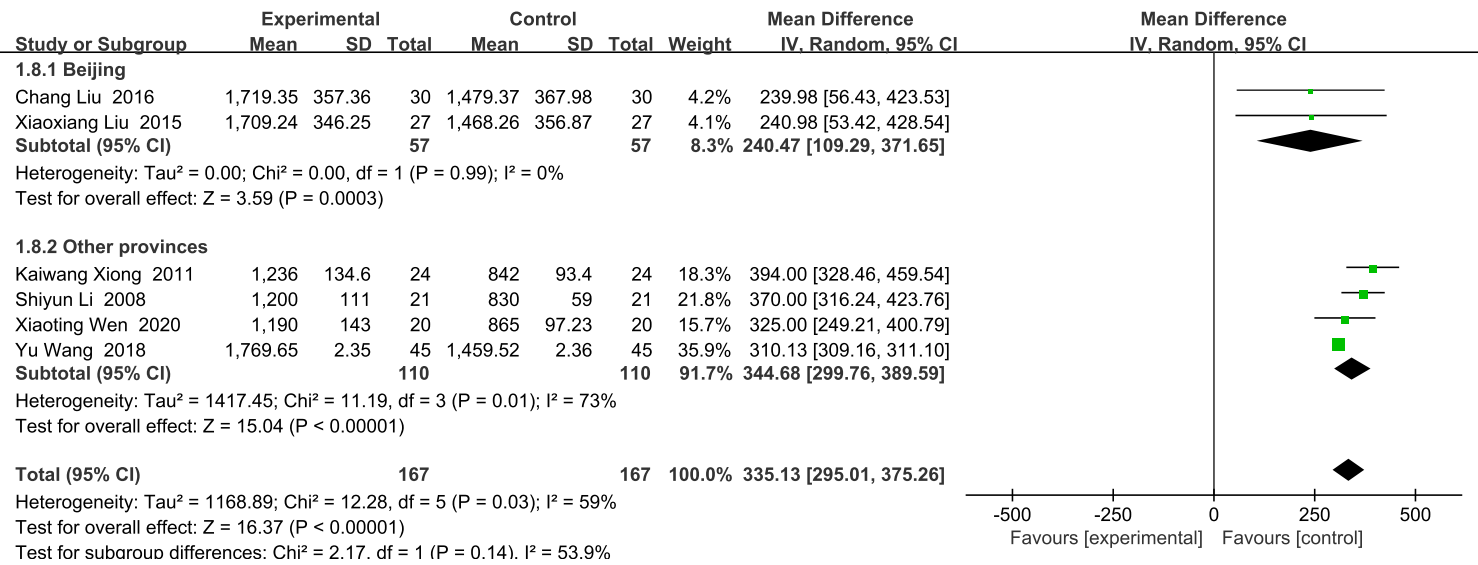

Supplement: Supplementary Materials — Supplementary Table 1: PubMed search strategy. Supplementary Figure 1: subgroup analysis of 24h urine volume (age). Supplementary Figure 2: subgroup analysis of 24h urine volume (region). Supplementary Figure 3: subgroup analysis of 24h urine protein quantification (age). Supplementary Figure 4: subgroup analysis of 24h urine protein quantification (control treatment). Supplementary Figure 5: subgroup analysis of 24h urine protein quantification (course of treatment). Supplementary Figure 6: subgroup analysis of 24h urine protein quantification (region). Supplementary Figure 7: subgroup analysis of serum creatinine (course of treatment). Supplementary Figure 8: subgroup analysis of blood creatinine (region). Supplementary Figure 9: subgroup analysis of blood urea nitrogen (course of treatment). Supplementary Figure 10: subgroup analysis of blood urea nitrogen (region). Supplementary Figure 11: subgroup analysis of urinary albumin excretion rates (age). Supplementary Figure 12: subgroup analysis of urinary albumin excretion rates (course of treatment). Supplementary Figure 13: subgroup analysis of urinary albumin excretion rates (region). Supplementary Figure 14: subgroup analysis of fasting blood glucose (age). Supplementary Figure 15: subgroup analysis of fasting blood glucose (control treatment). Supplementary Figure 16: subgroup analysis of fasting blood glucose (course of treatment). Supplementary Figure 17: subgroup analysis of fasting blood glucose (region). Supplementary Figure 18: subgroup analysis of fasting blood glucose (adverse effects). Supplementary Figure 19: subgroup analysis of glycated hemoglobin (age). Supplementary Figure 20: subgroup analysis of glycated hemoglobin (region). Supplementary Figure 21: subgroup analysis of TC (age). Supplementary Figure 22: subgroup analysis of TC (control treatment). Supplementary Figure 23: subgroup analysis of TC (course of treatment). Supplementary Figure 24: subgroup analysis of TC (region). Supplementary Figu [file 1720749.f1.zip › Fig2 Subgroup analysis of 24h urine volume (region).pdf]

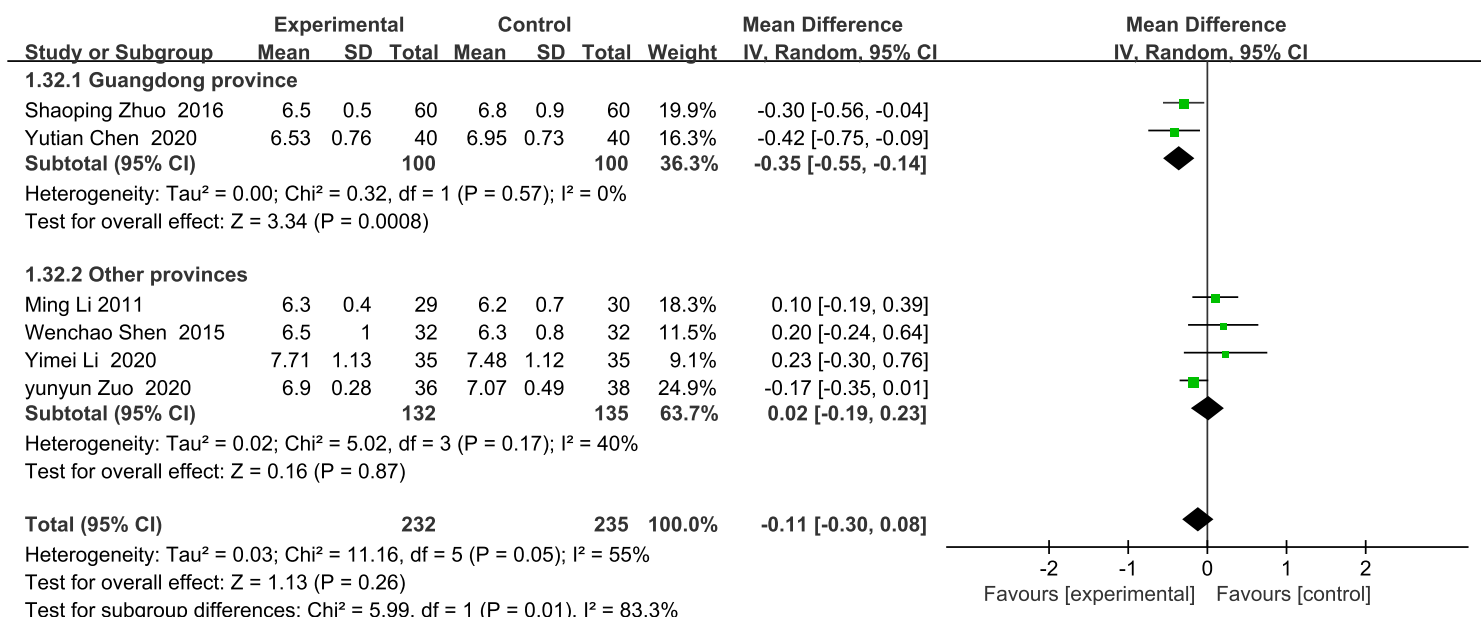

Supplement: Supplementary Materials — Supplementary Table 1: PubMed search strategy. Supplementary Figure 1: subgroup analysis of 24h urine volume (age). Supplementary Figure 2: subgroup analysis of 24h urine volume (region). Supplementary Figure 3: subgroup analysis of 24h urine protein quantification (age). Supplementary Figure 4: subgroup analysis of 24h urine protein quantification (control treatment). Supplementary Figure 5: subgroup analysis of 24h urine protein quantification (course of treatment). Supplementary Figure 6: subgroup analysis of 24h urine protein quantification (region). Supplementary Figure 7: subgroup analysis of serum creatinine (course of treatment). Supplementary Figure 8: subgroup analysis of blood creatinine (region). Supplementary Figure 9: subgroup analysis of blood urea nitrogen (course of treatment). Supplementary Figure 10: subgroup analysis of blood urea nitrogen (region). Supplementary Figure 11: subgroup analysis of urinary albumin excretion rates (age). Supplementary Figure 12: subgroup analysis of urinary albumin excretion rates (course of treatment). Supplementary Figure 13: subgroup analysis of urinary albumin excretion rates (region). Supplementary Figure 14: subgroup analysis of fasting blood glucose (age). Supplementary Figure 15: subgroup analysis of fasting blood glucose (control treatment). Supplementary Figure 16: subgroup analysis of fasting blood glucose (course of treatment). Supplementary Figure 17: subgroup analysis of fasting blood glucose (region). Supplementary Figure 18: subgroup analysis of fasting blood glucose (adverse effects). Supplementary Figure 19: subgroup analysis of glycated hemoglobin (age). Supplementary Figure 20: subgroup analysis of glycated hemoglobin (region). Supplementary Figure 21: subgroup analysis of TC (age). Supplementary Figure 22: subgroup analysis of TC (control treatment). Supplementary Figure 23: subgroup analysis of TC (course of treatment). Supplementary Figure 24: subgroup analysis of TC (region). Supplementary Figu [file 1720749.f1.zip › Fig20 Subgroup analysis of glycated haemoglobin (region).pdf]

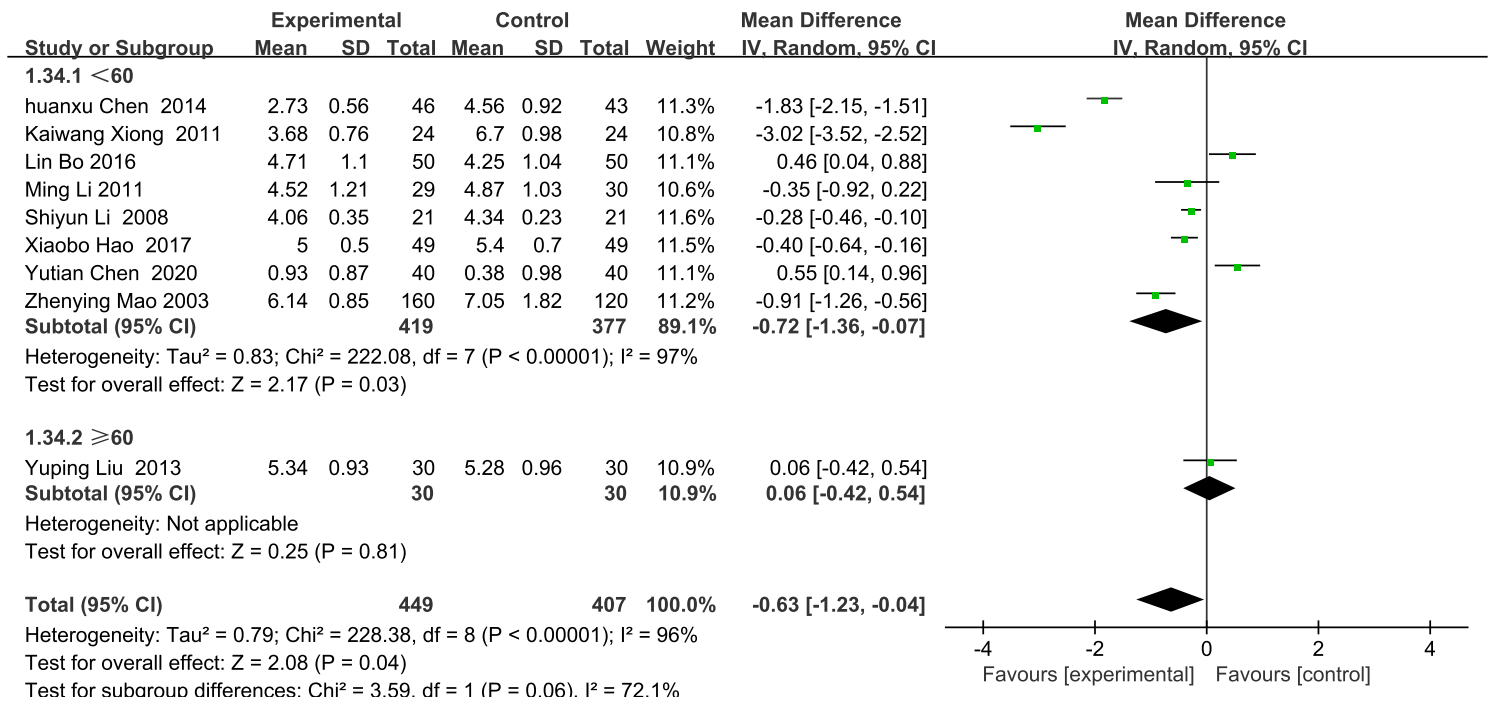

Supplement: Supplementary Materials — Supplementary Table 1: PubMed search strategy. Supplementary Figure 1: subgroup analysis of 24h urine volume (age). Supplementary Figure 2: subgroup analysis of 24h urine volume (region). Supplementary Figure 3: subgroup analysis of 24h urine protein quantification (age). Supplementary Figure 4: subgroup analysis of 24h urine protein quantification (control treatment). Supplementary Figure 5: subgroup analysis of 24h urine protein quantification (course of treatment). Supplementary Figure 6: subgroup analysis of 24h urine protein quantification (region). Supplementary Figure 7: subgroup analysis of serum creatinine (course of treatment). Supplementary Figure 8: subgroup analysis of blood creatinine (region). Supplementary Figure 9: subgroup analysis of blood urea nitrogen (course of treatment). Supplementary Figure 10: subgroup analysis of blood urea nitrogen (region). Supplementary Figure 11: subgroup analysis of urinary albumin excretion rates (age). Supplementary Figure 12: subgroup analysis of urinary albumin excretion rates (course of treatment). Supplementary Figure 13: subgroup analysis of urinary albumin excretion rates (region). Supplementary Figure 14: subgroup analysis of fasting blood glucose (age). Supplementary Figure 15: subgroup analysis of fasting blood glucose (control treatment). Supplementary Figure 16: subgroup analysis of fasting blood glucose (course of treatment). Supplementary Figure 17: subgroup analysis of fasting blood glucose (region). Supplementary Figure 18: subgroup analysis of fasting blood glucose (adverse effects). Supplementary Figure 19: subgroup analysis of glycated hemoglobin (age). Supplementary Figure 20: subgroup analysis of glycated hemoglobin (region). Supplementary Figure 21: subgroup analysis of TC (age). Supplementary Figure 22: subgroup analysis of TC (control treatment). Supplementary Figure 23: subgroup analysis of TC (course of treatment). Supplementary Figure 24: subgroup analysis of TC (region). Supplementary Figu [file 1720749.f1.zip › Fig21 Subgroup analysis of TC (age).pdf]

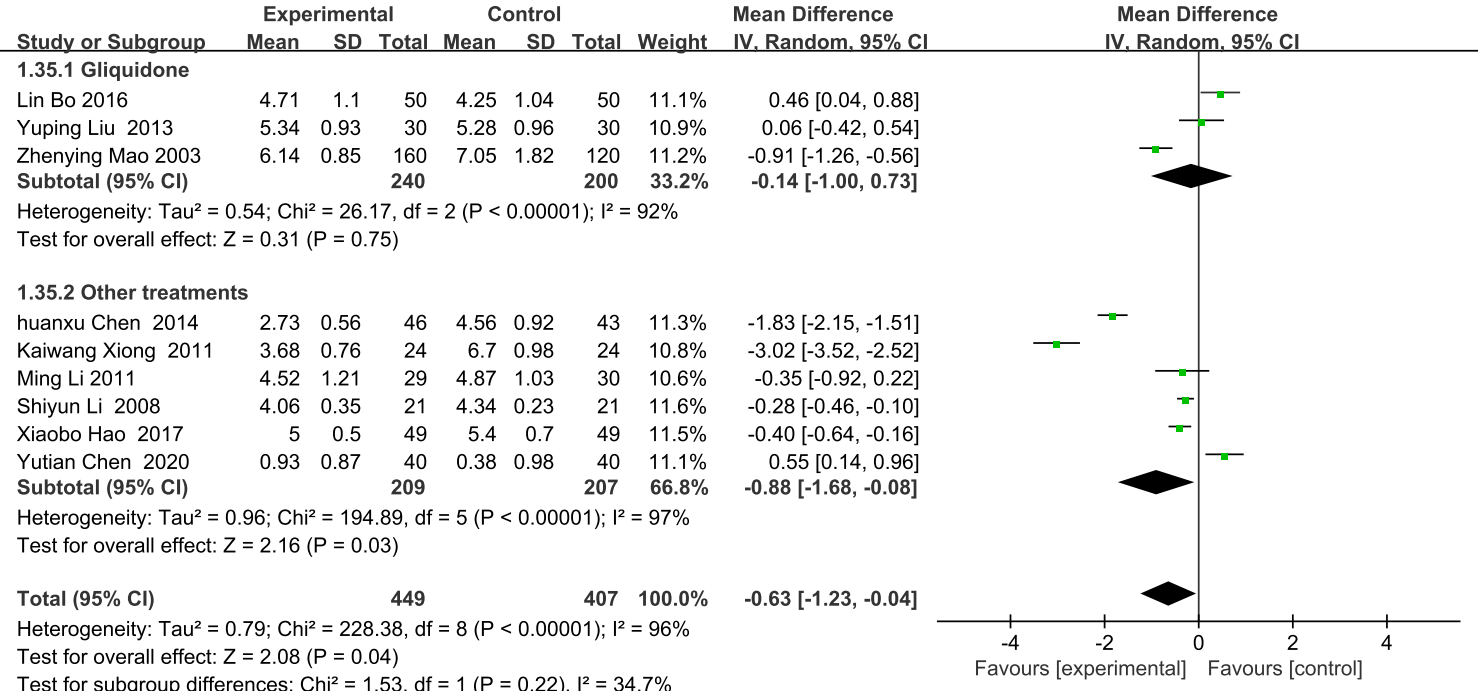

Supplement: Supplementary Materials — Supplementary Table 1: PubMed search strategy. Supplementary Figure 1: subgroup analysis of 24h urine volume (age). Supplementary Figure 2: subgroup analysis of 24h urine volume (region). Supplementary Figure 3: subgroup analysis of 24h urine protein quantification (age). Supplementary Figure 4: subgroup analysis of 24h urine protein quantification (control treatment). Supplementary Figure 5: subgroup analysis of 24h urine protein quantification (course of treatment). Supplementary Figure 6: subgroup analysis of 24h urine protein quantification (region). Supplementary Figure 7: subgroup analysis of serum creatinine (course of treatment). Supplementary Figure 8: subgroup analysis of blood creatinine (region). Supplementary Figure 9: subgroup analysis of blood urea nitrogen (course of treatment). Supplementary Figure 10: subgroup analysis of blood urea nitrogen (region). Supplementary Figure 11: subgroup analysis of urinary albumin excretion rates (age). Supplementary Figure 12: subgroup analysis of urinary albumin excretion rates (course of treatment). Supplementary Figure 13: subgroup analysis of urinary albumin excretion rates (region). Supplementary Figure 14: subgroup analysis of fasting blood glucose (age). Supplementary Figure 15: subgroup analysis of fasting blood glucose (control treatment). Supplementary Figure 16: subgroup analysis of fasting blood glucose (course of treatment). Supplementary Figure 17: subgroup analysis of fasting blood glucose (region). Supplementary Figure 18: subgroup analysis of fasting blood glucose (adverse effects). Supplementary Figure 19: subgroup analysis of glycated hemoglobin (age). Supplementary Figure 20: subgroup analysis of glycated hemoglobin (region). Supplementary Figure 21: subgroup analysis of TC (age). Supplementary Figure 22: subgroup analysis of TC (control treatment). Supplementary Figure 23: subgroup analysis of TC (course of treatment). Supplementary Figure 24: subgroup analysis of TC (region). Supplementary Figu [file 1720749.f1.zip › Fig22 Subgroup analysis of TC (control treatment).pdf]

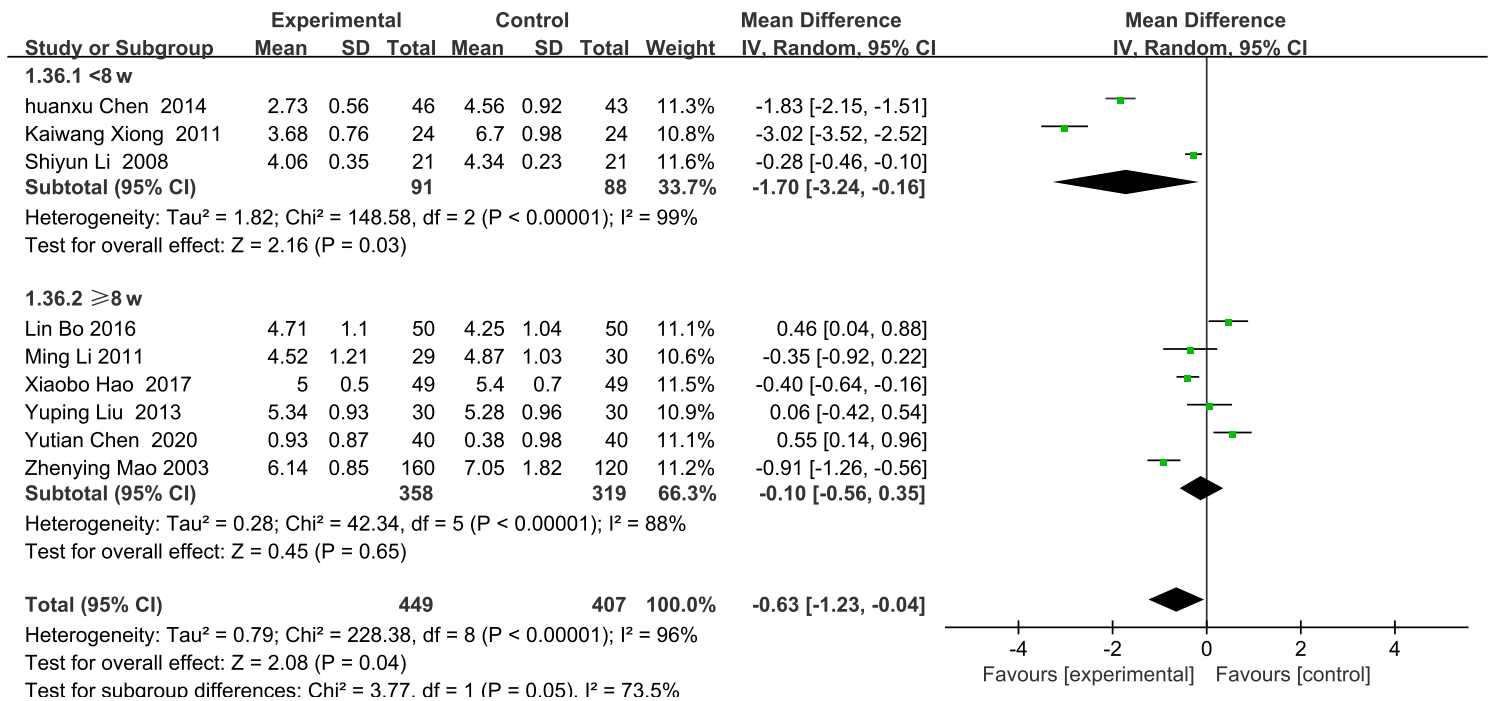

Supplement: Supplementary Materials — Supplementary Table 1: PubMed search strategy. Supplementary Figure 1: subgroup analysis of 24h urine volume (age). Supplementary Figure 2: subgroup analysis of 24h urine volume (region). Supplementary Figure 3: subgroup analysis of 24h urine protein quantification (age). Supplementary Figure 4: subgroup analysis of 24h urine protein quantification (control treatment). Supplementary Figure 5: subgroup analysis of 24h urine protein quantification (course of treatment). Supplementary Figure 6: subgroup analysis of 24h urine protein quantification (region). Supplementary Figure 7: subgroup analysis of serum creatinine (course of treatment). Supplementary Figure 8: subgroup analysis of blood creatinine (region). Supplementary Figure 9: subgroup analysis of blood urea nitrogen (course of treatment). Supplementary Figure 10: subgroup analysis of blood urea nitrogen (region). Supplementary Figure 11: subgroup analysis of urinary albumin excretion rates (age). Supplementary Figure 12: subgroup analysis of urinary albumin excretion rates (course of treatment). Supplementary Figure 13: subgroup analysis of urinary albumin excretion rates (region). Supplementary Figure 14: subgroup analysis of fasting blood glucose (age). Supplementary Figure 15: subgroup analysis of fasting blood glucose (control treatment). Supplementary Figure 16: subgroup analysis of fasting blood glucose (course of treatment). Supplementary Figure 17: subgroup analysis of fasting blood glucose (region). Supplementary Figure 18: subgroup analysis of fasting blood glucose (adverse effects). Supplementary Figure 19: subgroup analysis of glycated hemoglobin (age). Supplementary Figure 20: subgroup analysis of glycated hemoglobin (region). Supplementary Figure 21: subgroup analysis of TC (age). Supplementary Figure 22: subgroup analysis of TC (control treatment). Supplementary Figure 23: subgroup analysis of TC (course of treatment). Supplementary Figure 24: subgroup analysis of TC (region). Supplementary Figu [file 1720749.f1.zip › Fig23 Subgroup analysis of TC (course of treatment).pdf]

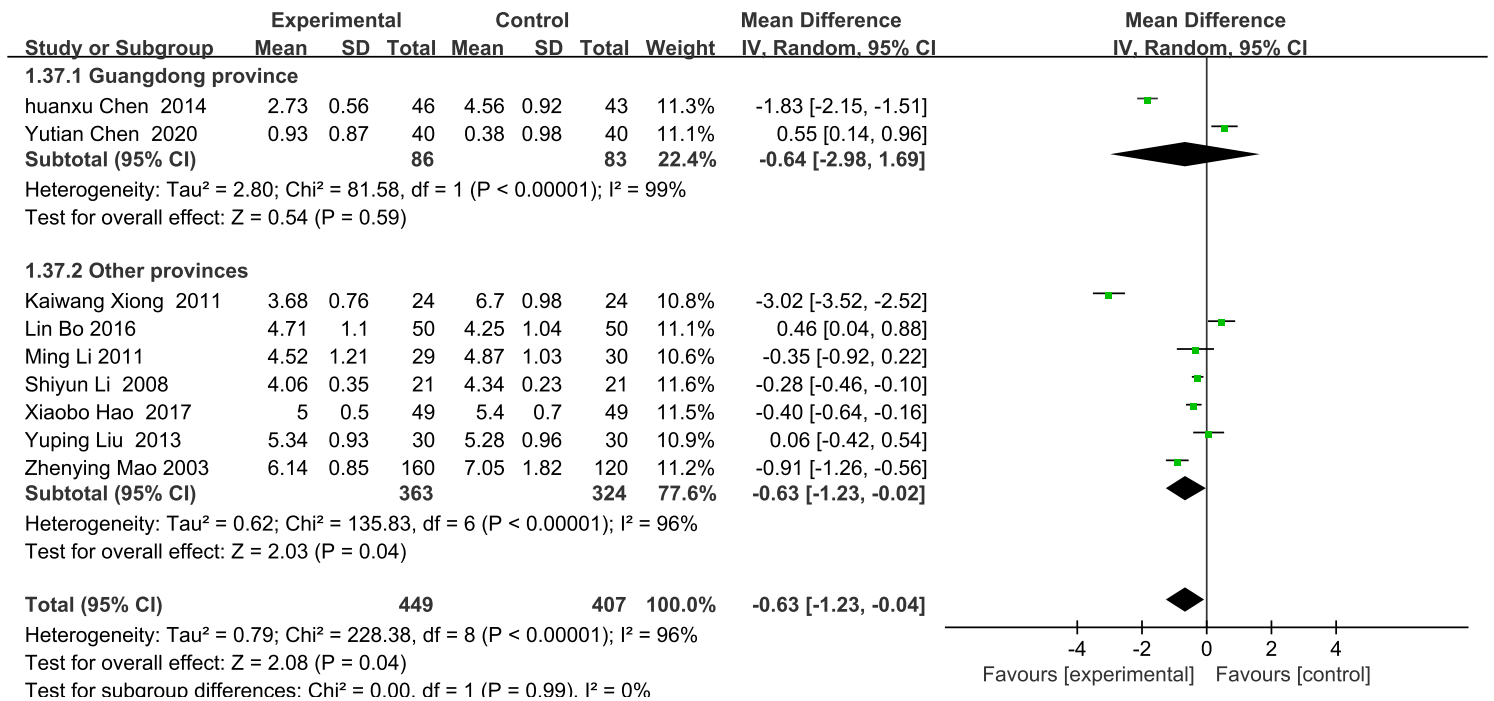

Supplement: Supplementary Materials — Supplementary Table 1: PubMed search strategy. Supplementary Figure 1: subgroup analysis of 24h urine volume (age). Supplementary Figure 2: subgroup analysis of 24h urine volume (region). Supplementary Figure 3: subgroup analysis of 24h urine protein quantification (age). Supplementary Figure 4: subgroup analysis of 24h urine protein quantification (control treatment). Supplementary Figure 5: subgroup analysis of 24h urine protein quantification (course of treatment). Supplementary Figure 6: subgroup analysis of 24h urine protein quantification (region). Supplementary Figure 7: subgroup analysis of serum creatinine (course of treatment). Supplementary Figure 8: subgroup analysis of blood creatinine (region). Supplementary Figure 9: subgroup analysis of blood urea nitrogen (course of treatment). Supplementary Figure 10: subgroup analysis of blood urea nitrogen (region). Supplementary Figure 11: subgroup analysis of urinary albumin excretion rates (age). Supplementary Figure 12: subgroup analysis of urinary albumin excretion rates (course of treatment). Supplementary Figure 13: subgroup analysis of urinary albumin excretion rates (region). Supplementary Figure 14: subgroup analysis of fasting blood glucose (age). Supplementary Figure 15: subgroup analysis of fasting blood glucose (control treatment). Supplementary Figure 16: subgroup analysis of fasting blood glucose (course of treatment). Supplementary Figure 17: subgroup analysis of fasting blood glucose (region). Supplementary Figure 18: subgroup analysis of fasting blood glucose (adverse effects). Supplementary Figure 19: subgroup analysis of glycated hemoglobin (age). Supplementary Figure 20: subgroup analysis of glycated hemoglobin (region). Supplementary Figure 21: subgroup analysis of TC (age). Supplementary Figure 22: subgroup analysis of TC (control treatment). Supplementary Figure 23: subgroup analysis of TC (course of treatment). Supplementary Figure 24: subgroup analysis of TC (region). Supplementary Figu [file 1720749.f1.zip › Fig24 Subgroup analysis of TC (region).pdf]

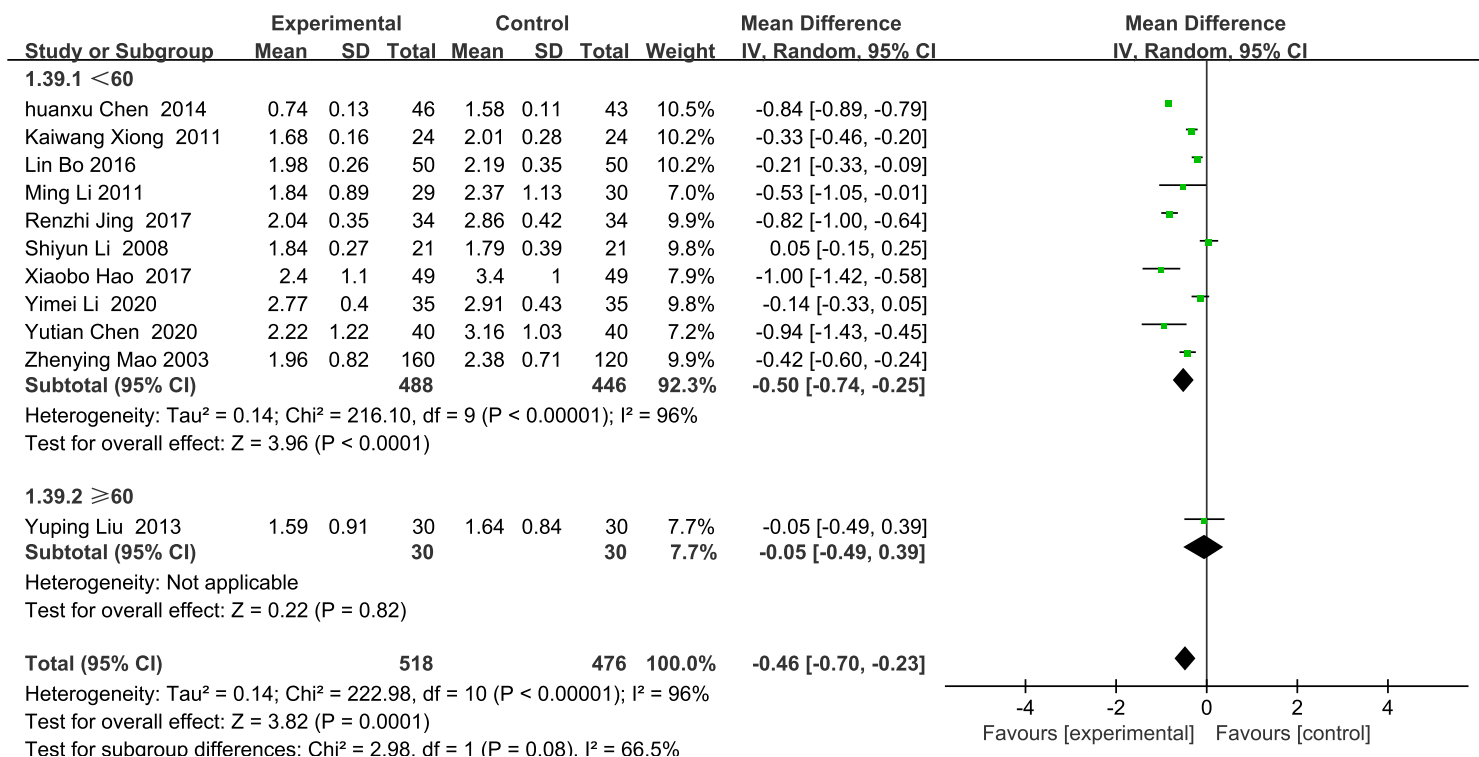

Supplement: Supplementary Materials — Supplementary Table 1: PubMed search strategy. Supplementary Figure 1: subgroup analysis of 24h urine volume (age). Supplementary Figure 2: subgroup analysis of 24h urine volume (region). Supplementary Figure 3: subgroup analysis of 24h urine protein quantification (age). Supplementary Figure 4: subgroup analysis of 24h urine protein quantification (control treatment). Supplementary Figure 5: subgroup analysis of 24h urine protein quantification (course of treatment). Supplementary Figure 6: subgroup analysis of 24h urine protein quantification (region). Supplementary Figure 7: subgroup analysis of serum creatinine (course of treatment). Supplementary Figure 8: subgroup analysis of blood creatinine (region). Supplementary Figure 9: subgroup analysis of blood urea nitrogen (course of treatment). Supplementary Figure 10: subgroup analysis of blood urea nitrogen (region). Supplementary Figure 11: subgroup analysis of urinary albumin excretion rates (age). Supplementary Figure 12: subgroup analysis of urinary albumin excretion rates (course of treatment). Supplementary Figure 13: subgroup analysis of urinary albumin excretion rates (region). Supplementary Figure 14: subgroup analysis of fasting blood glucose (age). Supplementary Figure 15: subgroup analysis of fasting blood glucose (control treatment). Supplementary Figure 16: subgroup analysis of fasting blood glucose (course of treatment). Supplementary Figure 17: subgroup analysis of fasting blood glucose (region). Supplementary Figure 18: subgroup analysis of fasting blood glucose (adverse effects). Supplementary Figure 19: subgroup analysis of glycated hemoglobin (age). Supplementary Figure 20: subgroup analysis of glycated hemoglobin (region). Supplementary Figure 21: subgroup analysis of TC (age). Supplementary Figure 22: subgroup analysis of TC (control treatment). Supplementary Figure 23: subgroup analysis of TC (course of treatment). Supplementary Figure 24: subgroup analysis of TC (region). Supplementary Figu [file 1720749.f1.zip › Fig25 Subgroup analysis of TG (age).pdf]

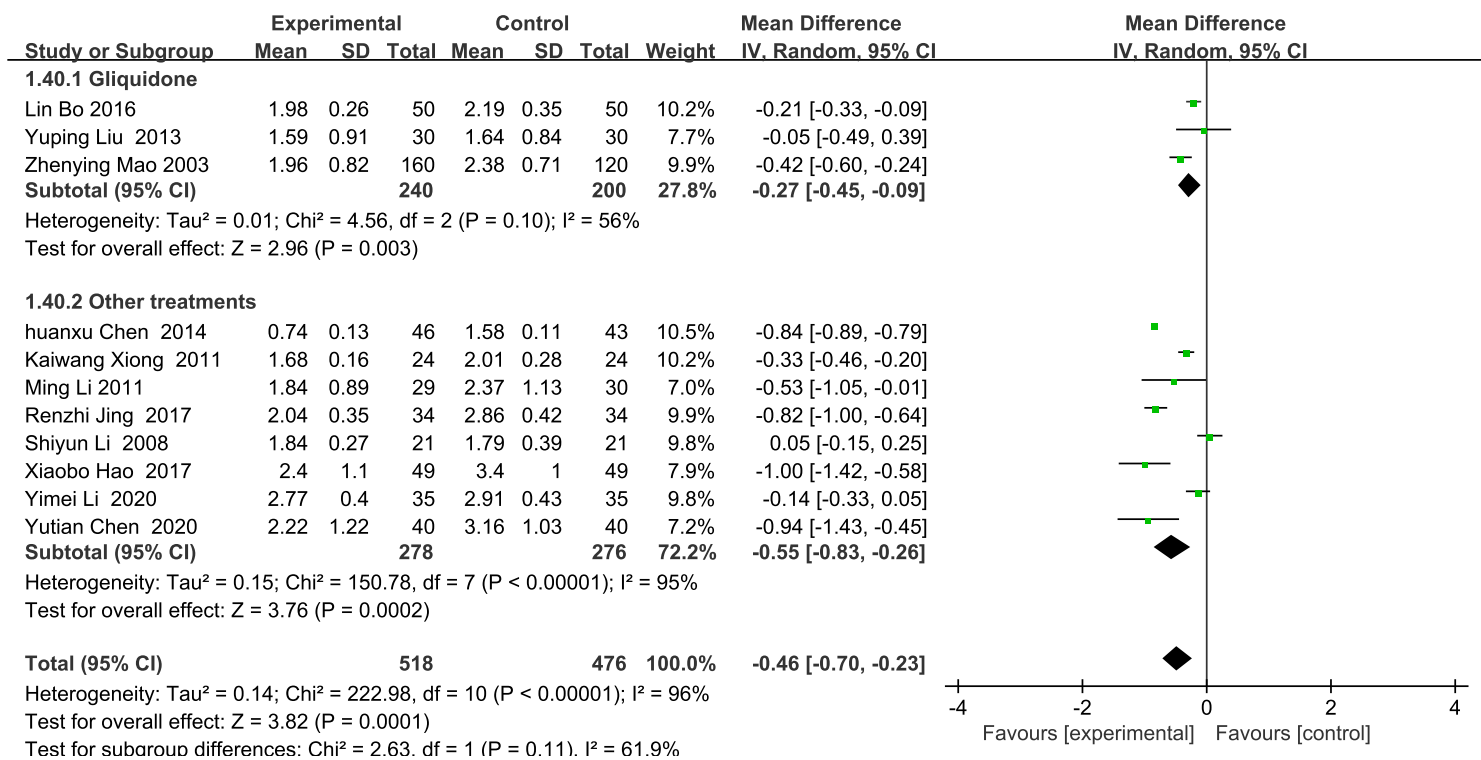

Supplement: Supplementary Materials — Supplementary Table 1: PubMed search strategy. Supplementary Figure 1: subgroup analysis of 24h urine volume (age). Supplementary Figure 2: subgroup analysis of 24h urine volume (region). Supplementary Figure 3: subgroup analysis of 24h urine protein quantification (age). Supplementary Figure 4: subgroup analysis of 24h urine protein quantification (control treatment). Supplementary Figure 5: subgroup analysis of 24h urine protein quantification (course of treatment). Supplementary Figure 6: subgroup analysis of 24h urine protein quantification (region). Supplementary Figure 7: subgroup analysis of serum creatinine (course of treatment). Supplementary Figure 8: subgroup analysis of blood creatinine (region). Supplementary Figure 9: subgroup analysis of blood urea nitrogen (course of treatment). Supplementary Figure 10: subgroup analysis of blood urea nitrogen (region). Supplementary Figure 11: subgroup analysis of urinary albumin excretion rates (age). Supplementary Figure 12: subgroup analysis of urinary albumin excretion rates (course of treatment). Supplementary Figure 13: subgroup analysis of urinary albumin excretion rates (region). Supplementary Figure 14: subgroup analysis of fasting blood glucose (age). Supplementary Figure 15: subgroup analysis of fasting blood glucose (control treatment). Supplementary Figure 16: subgroup analysis of fasting blood glucose (course of treatment). Supplementary Figure 17: subgroup analysis of fasting blood glucose (region). Supplementary Figure 18: subgroup analysis of fasting blood glucose (adverse effects). Supplementary Figure 19: subgroup analysis of glycated hemoglobin (age). Supplementary Figure 20: subgroup analysis of glycated hemoglobin (region). Supplementary Figure 21: subgroup analysis of TC (age). Supplementary Figure 22: subgroup analysis of TC (control treatment). Supplementary Figure 23: subgroup analysis of TC (course of treatment). Supplementary Figure 24: subgroup analysis of TC (region). Supplementary Figu [file 1720749.f1.zip › Fig26 Subgroup analysis of TG (control treatment).pdf]

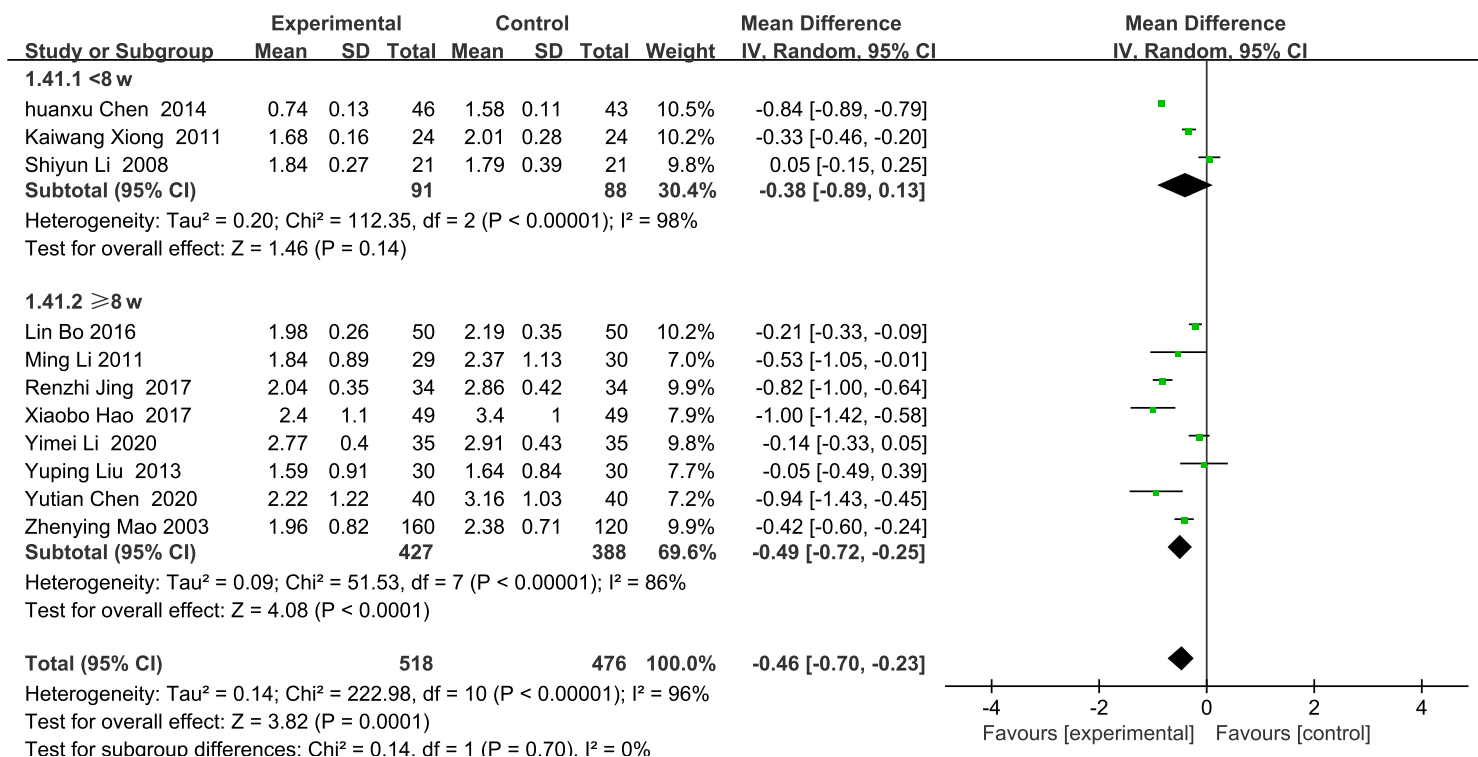

Supplement: Supplementary Materials — Supplementary Table 1: PubMed search strategy. Supplementary Figure 1: subgroup analysis of 24h urine volume (age). Supplementary Figure 2: subgroup analysis of 24h urine volume (region). Supplementary Figure 3: subgroup analysis of 24h urine protein quantification (age). Supplementary Figure 4: subgroup analysis of 24h urine protein quantification (control treatment). Supplementary Figure 5: subgroup analysis of 24h urine protein quantification (course of treatment). Supplementary Figure 6: subgroup analysis of 24h urine protein quantification (region). Supplementary Figure 7: subgroup analysis of serum creatinine (course of treatment). Supplementary Figure 8: subgroup analysis of blood creatinine (region). Supplementary Figure 9: subgroup analysis of blood urea nitrogen (course of treatment). Supplementary Figure 10: subgroup analysis of blood urea nitrogen (region). Supplementary Figure 11: subgroup analysis of urinary albumin excretion rates (age). Supplementary Figure 12: subgroup analysis of urinary albumin excretion rates (course of treatment). Supplementary Figure 13: subgroup analysis of urinary albumin excretion rates (region). Supplementary Figure 14: subgroup analysis of fasting blood glucose (age). Supplementary Figure 15: subgroup analysis of fasting blood glucose (control treatment). Supplementary Figure 16: subgroup analysis of fasting blood glucose (course of treatment). Supplementary Figure 17: subgroup analysis of fasting blood glucose (region). Supplementary Figure 18: subgroup analysis of fasting blood glucose (adverse effects). Supplementary Figure 19: subgroup analysis of glycated hemoglobin (age). Supplementary Figure 20: subgroup analysis of glycated hemoglobin (region). Supplementary Figure 21: subgroup analysis of TC (age). Supplementary Figure 22: subgroup analysis of TC (control treatment). Supplementary Figure 23: subgroup analysis of TC (course of treatment). Supplementary Figure 24: subgroup analysis of TC (region). Supplementary Figu [file 1720749.f1.zip › Fig27 Subgroup analysis of TG (course of treatment).pdf]

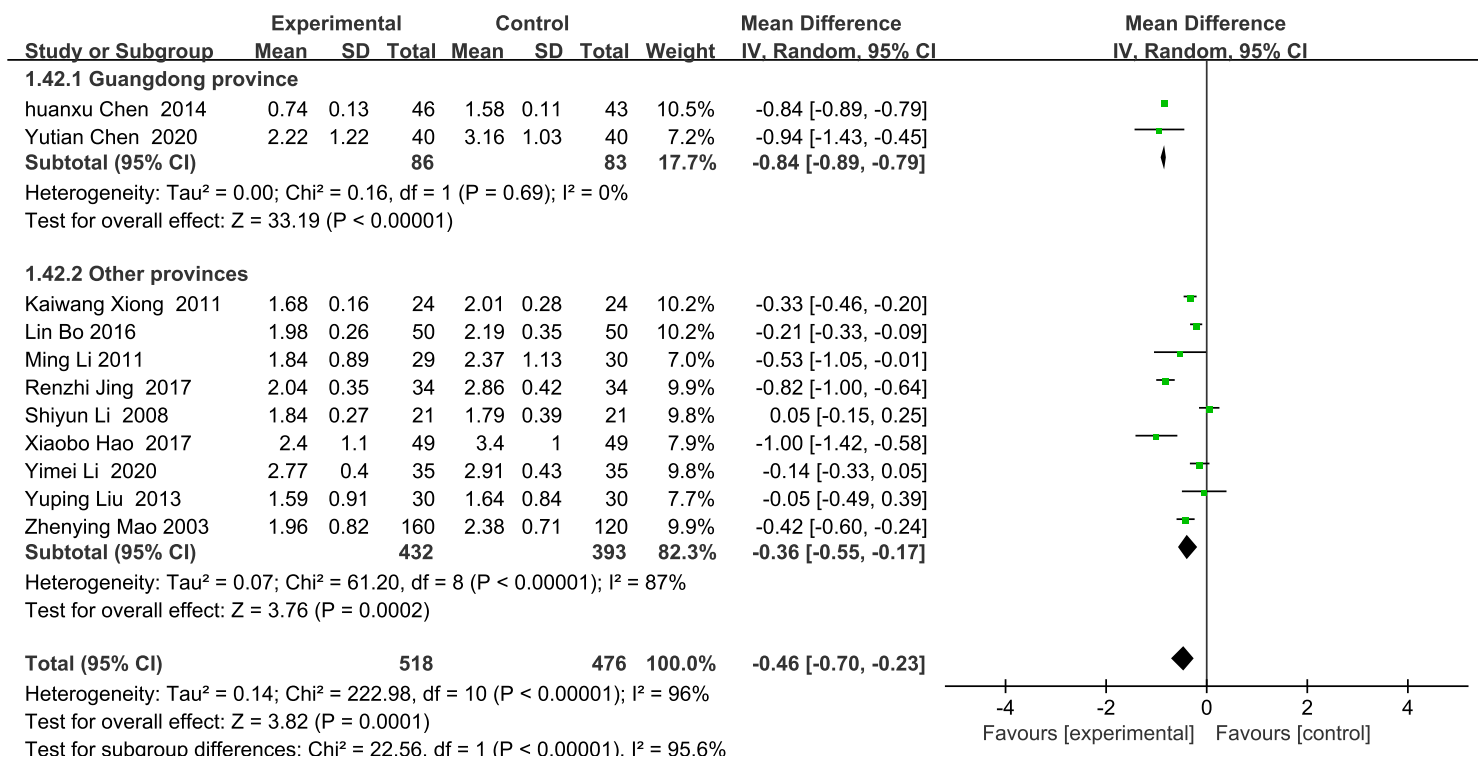

Supplement: Supplementary Materials — Supplementary Table 1: PubMed search strategy. Supplementary Figure 1: subgroup analysis of 24h urine volume (age). Supplementary Figure 2: subgroup analysis of 24h urine volume (region). Supplementary Figure 3: subgroup analysis of 24h urine protein quantification (age). Supplementary Figure 4: subgroup analysis of 24h urine protein quantification (control treatment). Supplementary Figure 5: subgroup analysis of 24h urine protein quantification (course of treatment). Supplementary Figure 6: subgroup analysis of 24h urine protein quantification (region). Supplementary Figure 7: subgroup analysis of serum creatinine (course of treatment). Supplementary Figure 8: subgroup analysis of blood creatinine (region). Supplementary Figure 9: subgroup analysis of blood urea nitrogen (course of treatment). Supplementary Figure 10: subgroup analysis of blood urea nitrogen (region). Supplementary Figure 11: subgroup analysis of urinary albumin excretion rates (age). Supplementary Figure 12: subgroup analysis of urinary albumin excretion rates (course of treatment). Supplementary Figure 13: subgroup analysis of urinary albumin excretion rates (region). Supplementary Figure 14: subgroup analysis of fasting blood glucose (age). Supplementary Figure 15: subgroup analysis of fasting blood glucose (control treatment). Supplementary Figure 16: subgroup analysis of fasting blood glucose (course of treatment). Supplementary Figure 17: subgroup analysis of fasting blood glucose (region). Supplementary Figure 18: subgroup analysis of fasting blood glucose (adverse effects). Supplementary Figure 19: subgroup analysis of glycated hemoglobin (age). Supplementary Figure 20: subgroup analysis of glycated hemoglobin (region). Supplementary Figure 21: subgroup analysis of TC (age). Supplementary Figure 22: subgroup analysis of TC (control treatment). Supplementary Figure 23: subgroup analysis of TC (course of treatment). Supplementary Figure 24: subgroup analysis of TC (region). Supplementary Figu [file 1720749.f1.zip › Fig28 Subgroup analysis of TG (region).pdf]

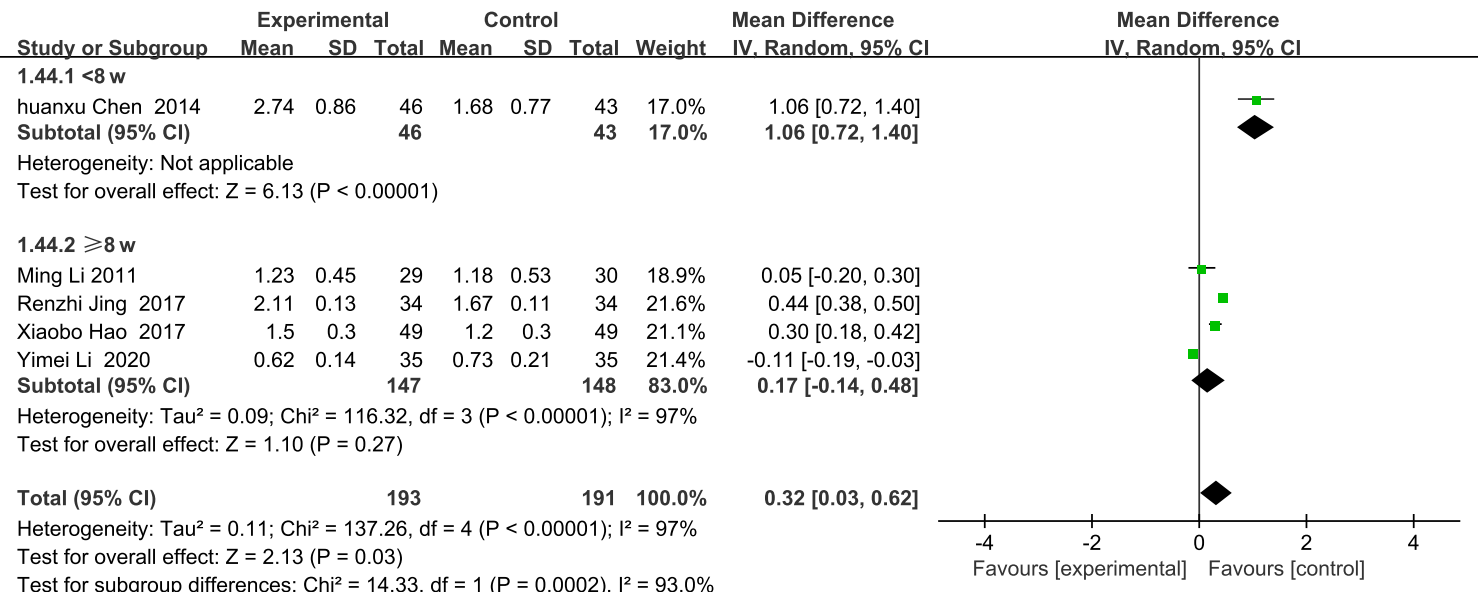

Supplement: Supplementary Materials — Supplementary Table 1: PubMed search strategy. Supplementary Figure 1: subgroup analysis of 24h urine volume (age). Supplementary Figure 2: subgroup analysis of 24h urine volume (region). Supplementary Figure 3: subgroup analysis of 24h urine protein quantification (age). Supplementary Figure 4: subgroup analysis of 24h urine protein quantification (control treatment). Supplementary Figure 5: subgroup analysis of 24h urine protein quantification (course of treatment). Supplementary Figure 6: subgroup analysis of 24h urine protein quantification (region). Supplementary Figure 7: subgroup analysis of serum creatinine (course of treatment). Supplementary Figure 8: subgroup analysis of blood creatinine (region). Supplementary Figure 9: subgroup analysis of blood urea nitrogen (course of treatment). Supplementary Figure 10: subgroup analysis of blood urea nitrogen (region). Supplementary Figure 11: subgroup analysis of urinary albumin excretion rates (age). Supplementary Figure 12: subgroup analysis of urinary albumin excretion rates (course of treatment). Supplementary Figure 13: subgroup analysis of urinary albumin excretion rates (region). Supplementary Figure 14: subgroup analysis of fasting blood glucose (age). Supplementary Figure 15: subgroup analysis of fasting blood glucose (control treatment). Supplementary Figure 16: subgroup analysis of fasting blood glucose (course of treatment). Supplementary Figure 17: subgroup analysis of fasting blood glucose (region). Supplementary Figure 18: subgroup analysis of fasting blood glucose (adverse effects). Supplementary Figure 19: subgroup analysis of glycated hemoglobin (age). Supplementary Figure 20: subgroup analysis of glycated hemoglobin (region). Supplementary Figure 21: subgroup analysis of TC (age). Supplementary Figure 22: subgroup analysis of TC (control treatment). Supplementary Figure 23: subgroup analysis of TC (course of treatment). Supplementary Figure 24: subgroup analysis of TC (region). Supplementary Figu [file 1720749.f1.zip › Fig29 Subgroup analysis of HDL (course of treatment).pdf]

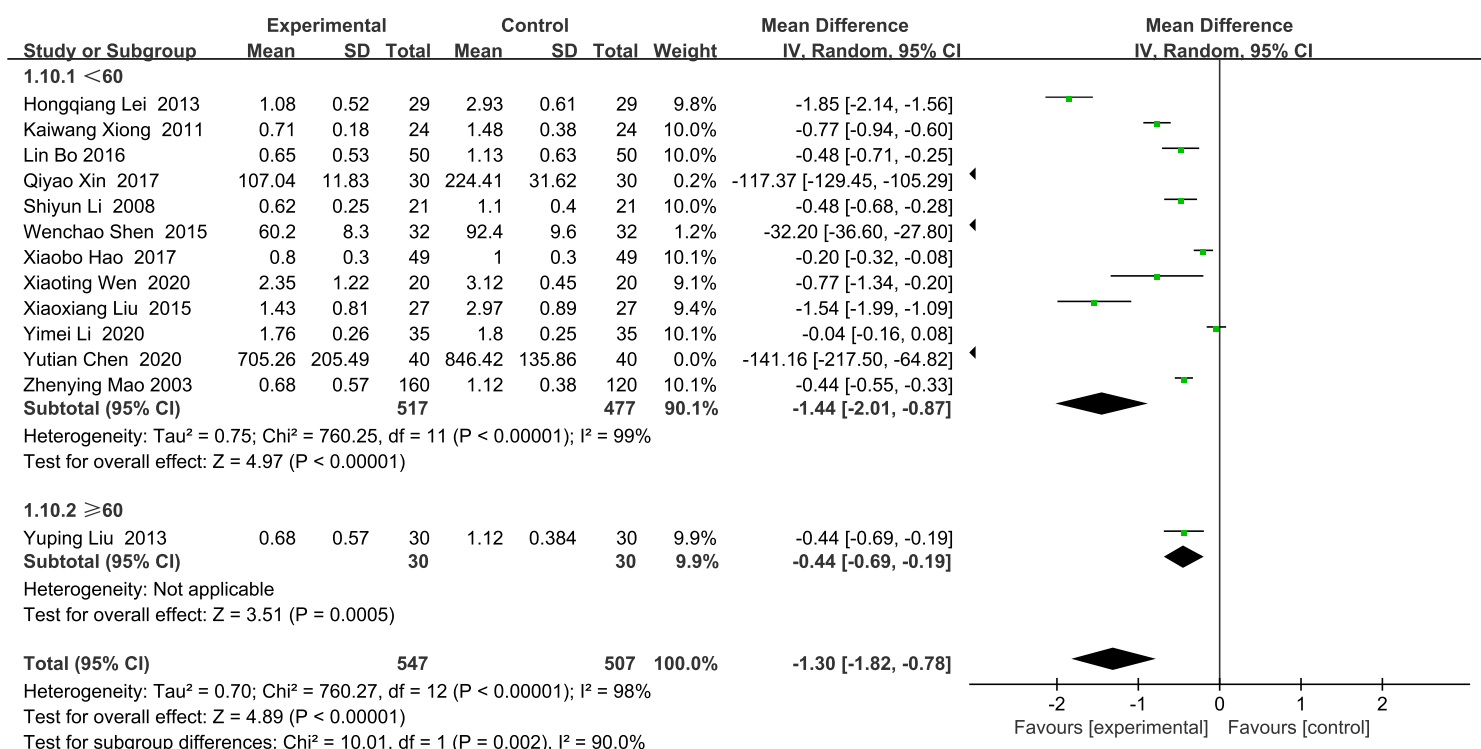

Supplement: Supplementary Materials — Supplementary Table 1: PubMed search strategy. Supplementary Figure 1: subgroup analysis of 24h urine volume (age). Supplementary Figure 2: subgroup analysis of 24h urine volume (region). Supplementary Figure 3: subgroup analysis of 24h urine protein quantification (age). Supplementary Figure 4: subgroup analysis of 24h urine protein quantification (control treatment). Supplementary Figure 5: subgroup analysis of 24h urine protein quantification (course of treatment). Supplementary Figure 6: subgroup analysis of 24h urine protein quantification (region). Supplementary Figure 7: subgroup analysis of serum creatinine (course of treatment). Supplementary Figure 8: subgroup analysis of blood creatinine (region). Supplementary Figure 9: subgroup analysis of blood urea nitrogen (course of treatment). Supplementary Figure 10: subgroup analysis of blood urea nitrogen (region). Supplementary Figure 11: subgroup analysis of urinary albumin excretion rates (age). Supplementary Figure 12: subgroup analysis of urinary albumin excretion rates (course of treatment). Supplementary Figure 13: subgroup analysis of urinary albumin excretion rates (region). Supplementary Figure 14: subgroup analysis of fasting blood glucose (age). Supplementary Figure 15: subgroup analysis of fasting blood glucose (control treatment). Supplementary Figure 16: subgroup analysis of fasting blood glucose (course of treatment). Supplementary Figure 17: subgroup analysis of fasting blood glucose (region). Supplementary Figure 18: subgroup analysis of fasting blood glucose (adverse effects). Supplementary Figure 19: subgroup analysis of glycated hemoglobin (age). Supplementary Figure 20: subgroup analysis of glycated hemoglobin (region). Supplementary Figure 21: subgroup analysis of TC (age). Supplementary Figure 22: subgroup analysis of TC (control treatment). Supplementary Figure 23: subgroup analysis of TC (course of treatment). Supplementary Figure 24: subgroup analysis of TC (region). Supplementary Figu [file 1720749.f1.zip › Fig3 Subgroup analysis of 24h urine protein quantification (age).pdf]

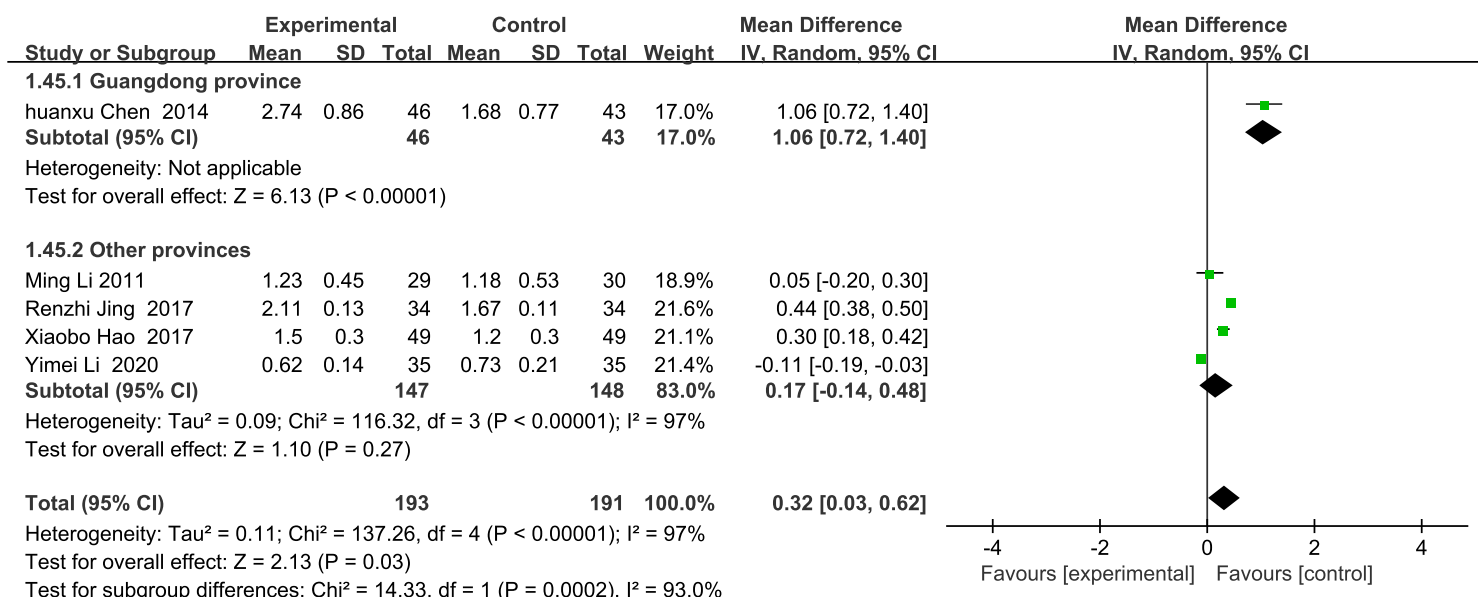

Supplement: Supplementary Materials — Supplementary Table 1: PubMed search strategy. Supplementary Figure 1: subgroup analysis of 24h urine volume (age). Supplementary Figure 2: subgroup analysis of 24h urine volume (region). Supplementary Figure 3: subgroup analysis of 24h urine protein quantification (age). Supplementary Figure 4: subgroup analysis of 24h urine protein quantification (control treatment). Supplementary Figure 5: subgroup analysis of 24h urine protein quantification (course of treatment). Supplementary Figure 6: subgroup analysis of 24h urine protein quantification (region). Supplementary Figure 7: subgroup analysis of serum creatinine (course of treatment). Supplementary Figure 8: subgroup analysis of blood creatinine (region). Supplementary Figure 9: subgroup analysis of blood urea nitrogen (course of treatment). Supplementary Figure 10: subgroup analysis of blood urea nitrogen (region). Supplementary Figure 11: subgroup analysis of urinary albumin excretion rates (age). Supplementary Figure 12: subgroup analysis of urinary albumin excretion rates (course of treatment). Supplementary Figure 13: subgroup analysis of urinary albumin excretion rates (region). Supplementary Figure 14: subgroup analysis of fasting blood glucose (age). Supplementary Figure 15: subgroup analysis of fasting blood glucose (control treatment). Supplementary Figure 16: subgroup analysis of fasting blood glucose (course of treatment). Supplementary Figure 17: subgroup analysis of fasting blood glucose (region). Supplementary Figure 18: subgroup analysis of fasting blood glucose (adverse effects). Supplementary Figure 19: subgroup analysis of glycated hemoglobin (age). Supplementary Figure 20: subgroup analysis of glycated hemoglobin (region). Supplementary Figure 21: subgroup analysis of TC (age). Supplementary Figure 22: subgroup analysis of TC (control treatment). Supplementary Figure 23: subgroup analysis of TC (course of treatment). Supplementary Figure 24: subgroup analysis of TC (region). Supplementary Figu [file 1720749.f1.zip › Fig30 Subgroup analysis of HDL (region).pdf]

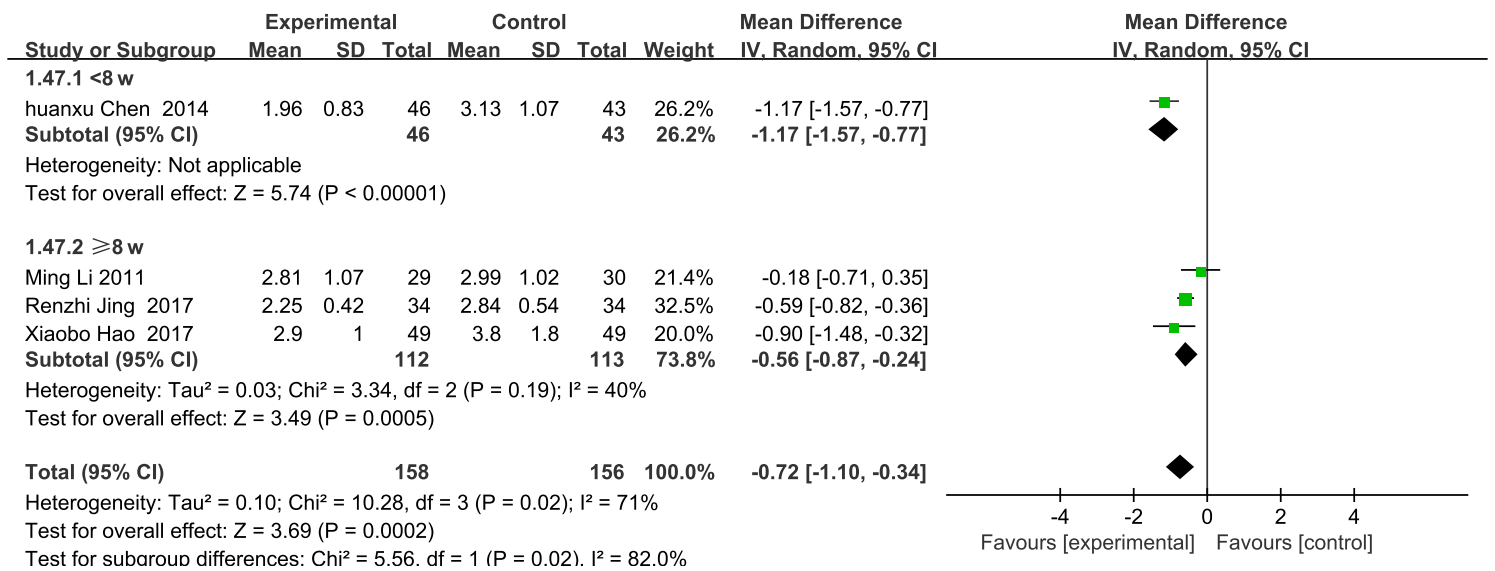

Supplement: Supplementary Materials — Supplementary Table 1: PubMed search strategy. Supplementary Figure 1: subgroup analysis of 24h urine volume (age). Supplementary Figure 2: subgroup analysis of 24h urine volume (region). Supplementary Figure 3: subgroup analysis of 24h urine protein quantification (age). Supplementary Figure 4: subgroup analysis of 24h urine protein quantification (control treatment). Supplementary Figure 5: subgroup analysis of 24h urine protein quantification (course of treatment). Supplementary Figure 6: subgroup analysis of 24h urine protein quantification (region). Supplementary Figure 7: subgroup analysis of serum creatinine (course of treatment). Supplementary Figure 8: subgroup analysis of blood creatinine (region). Supplementary Figure 9: subgroup analysis of blood urea nitrogen (course of treatment). Supplementary Figure 10: subgroup analysis of blood urea nitrogen (region). Supplementary Figure 11: subgroup analysis of urinary albumin excretion rates (age). Supplementary Figure 12: subgroup analysis of urinary albumin excretion rates (course of treatment). Supplementary Figure 13: subgroup analysis of urinary albumin excretion rates (region). Supplementary Figure 14: subgroup analysis of fasting blood glucose (age). Supplementary Figure 15: subgroup analysis of fasting blood glucose (control treatment). Supplementary Figure 16: subgroup analysis of fasting blood glucose (course of treatment). Supplementary Figure 17: subgroup analysis of fasting blood glucose (region). Supplementary Figure 18: subgroup analysis of fasting blood glucose (adverse effects). Supplementary Figure 19: subgroup analysis of glycated hemoglobin (age). Supplementary Figure 20: subgroup analysis of glycated hemoglobin (region). Supplementary Figure 21: subgroup analysis of TC (age). Supplementary Figure 22: subgroup analysis of TC (control treatment). Supplementary Figure 23: subgroup analysis of TC (course of treatment). Supplementary Figure 24: subgroup analysis of TC (region). Supplementary Figu [file 1720749.f1.zip › Fig31 Subgroup analysis of LDL (course of treatment).pdf]

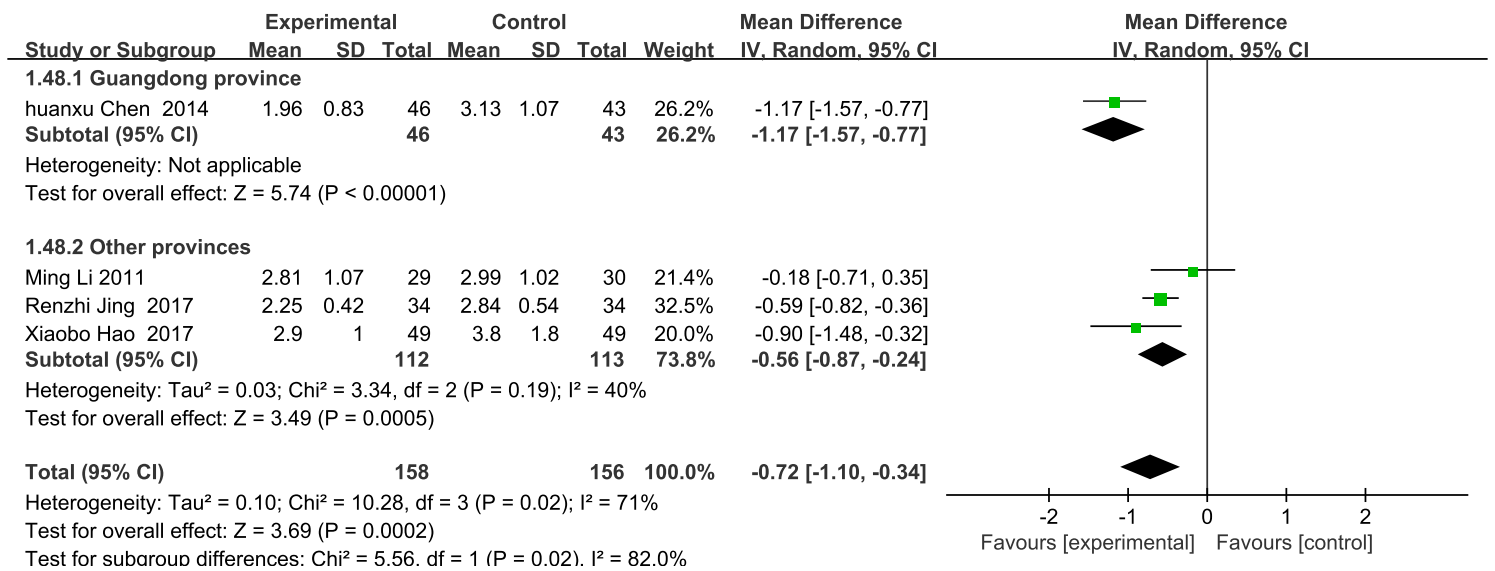

Supplement: Supplementary Materials — Supplementary Table 1: PubMed search strategy. Supplementary Figure 1: subgroup analysis of 24h urine volume (age). Supplementary Figure 2: subgroup analysis of 24h urine volume (region). Supplementary Figure 3: subgroup analysis of 24h urine protein quantification (age). Supplementary Figure 4: subgroup analysis of 24h urine protein quantification (control treatment). Supplementary Figure 5: subgroup analysis of 24h urine protein quantification (course of treatment). Supplementary Figure 6: subgroup analysis of 24h urine protein quantification (region). Supplementary Figure 7: subgroup analysis of serum creatinine (course of treatment). Supplementary Figure 8: subgroup analysis of blood creatinine (region). Supplementary Figure 9: subgroup analysis of blood urea nitrogen (course of treatment). Supplementary Figure 10: subgroup analysis of blood urea nitrogen (region). Supplementary Figure 11: subgroup analysis of urinary albumin excretion rates (age). Supplementary Figure 12: subgroup analysis of urinary albumin excretion rates (course of treatment). Supplementary Figure 13: subgroup analysis of urinary albumin excretion rates (region). Supplementary Figure 14: subgroup analysis of fasting blood glucose (age). Supplementary Figure 15: subgroup analysis of fasting blood glucose (control treatment). Supplementary Figure 16: subgroup analysis of fasting blood glucose (course of treatment). Supplementary Figure 17: subgroup analysis of fasting blood glucose (region). Supplementary Figure 18: subgroup analysis of fasting blood glucose (adverse effects). Supplementary Figure 19: subgroup analysis of glycated hemoglobin (age). Supplementary Figure 20: subgroup analysis of glycated hemoglobin (region). Supplementary Figure 21: subgroup analysis of TC (age). Supplementary Figure 22: subgroup analysis of TC (control treatment). Supplementary Figure 23: subgroup analysis of TC (course of treatment). Supplementary Figure 24: subgroup analysis of TC (region). Supplementary Figu [file 1720749.f1.zip › Fig32 Subgroup analysis of LDL (region).pdf]

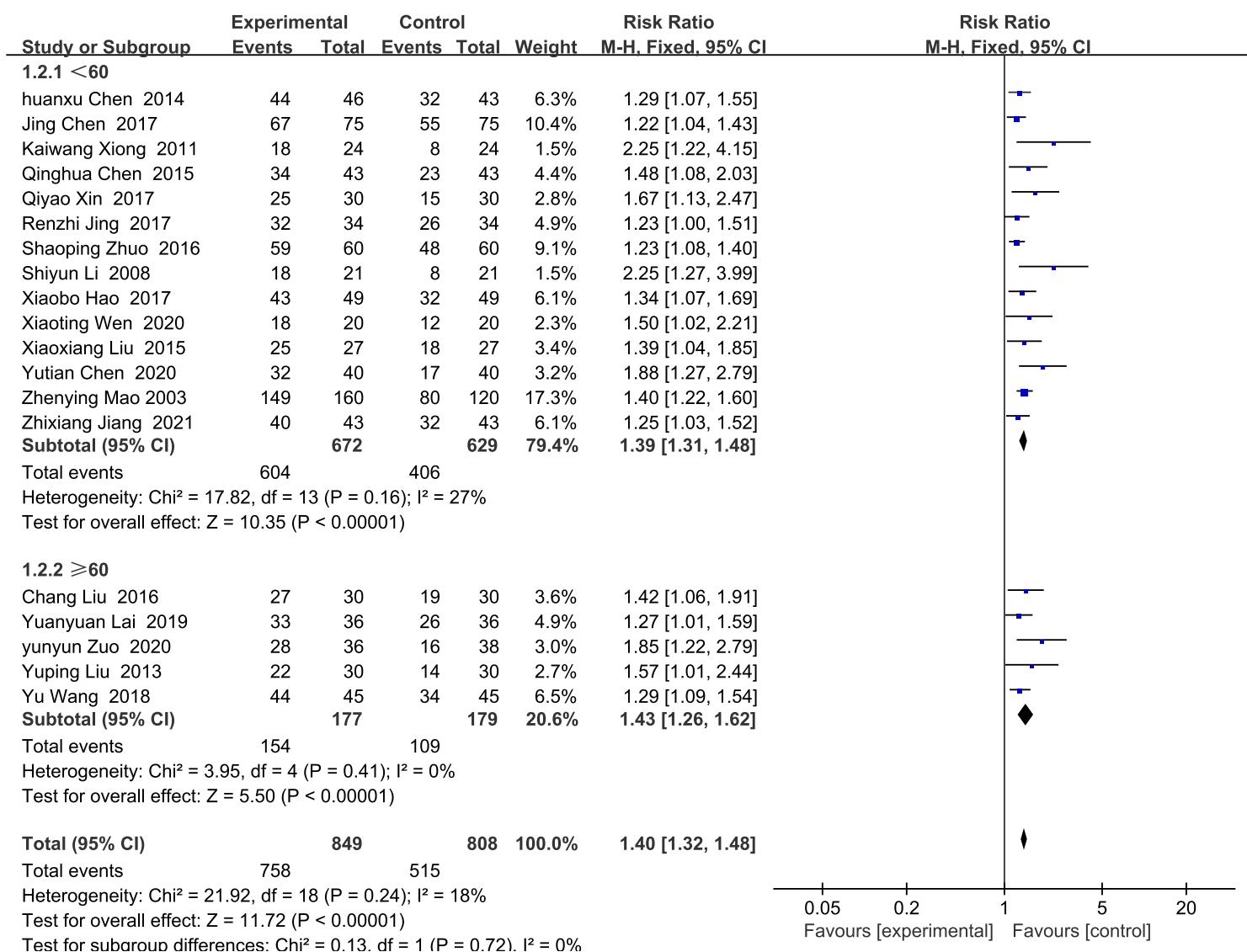

Supplement: Supplementary Materials — Supplementary Table 1: PubMed search strategy. Supplementary Figure 1: subgroup analysis of 24h urine volume (age). Supplementary Figure 2: subgroup analysis of 24h urine volume (region). Supplementary Figure 3: subgroup analysis of 24h urine protein quantification (age). Supplementary Figure 4: subgroup analysis of 24h urine protein quantification (control treatment). Supplementary Figure 5: subgroup analysis of 24h urine protein quantification (course of treatment). Supplementary Figure 6: subgroup analysis of 24h urine protein quantification (region). Supplementary Figure 7: subgroup analysis of serum creatinine (course of treatment). Supplementary Figure 8: subgroup analysis of blood creatinine (region). Supplementary Figure 9: subgroup analysis of blood urea nitrogen (course of treatment). Supplementary Figure 10: subgroup analysis of blood urea nitrogen (region). Supplementary Figure 11: subgroup analysis of urinary albumin excretion rates (age). Supplementary Figure 12: subgroup analysis of urinary albumin excretion rates (course of treatment). Supplementary Figure 13: subgroup analysis of urinary albumin excretion rates (region). Supplementary Figure 14: subgroup analysis of fasting blood glucose (age). Supplementary Figure 15: subgroup analysis of fasting blood glucose (control treatment). Supplementary Figure 16: subgroup analysis of fasting blood glucose (course of treatment). Supplementary Figure 17: subgroup analysis of fasting blood glucose (region). Supplementary Figure 18: subgroup analysis of fasting blood glucose (adverse effects). Supplementary Figure 19: subgroup analysis of glycated hemoglobin (age). Supplementary Figure 20: subgroup analysis of glycated hemoglobin (region). Supplementary Figure 21: subgroup analysis of TC (age). Supplementary Figure 22: subgroup analysis of TC (control treatment). Supplementary Figure 23: subgroup analysis of TC (course of treatment). Supplementary Figure 24: subgroup analysis of TC (region). Supplementary Figu [file 1720749.f1.zip › Fig33 Subgroup analysis of total effective rate (age).pdf]

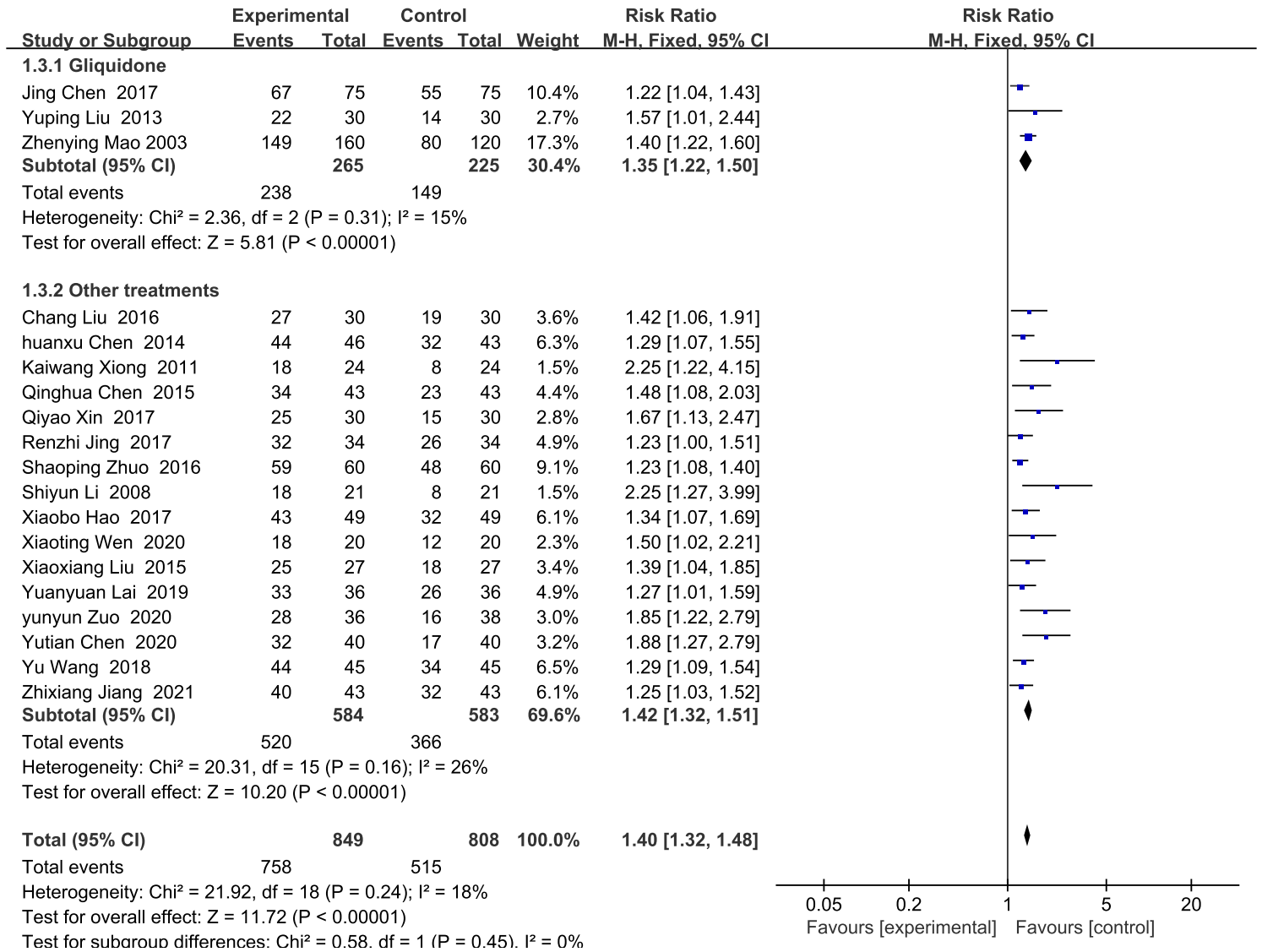

Supplement: Supplementary Materials — Supplementary Table 1: PubMed search strategy. Supplementary Figure 1: subgroup analysis of 24h urine volume (age). Supplementary Figure 2: subgroup analysis of 24h urine volume (region). Supplementary Figure 3: subgroup analysis of 24h urine protein quantification (age). Supplementary Figure 4: subgroup analysis of 24h urine protein quantification (control treatment). Supplementary Figure 5: subgroup analysis of 24h urine protein quantification (course of treatment). Supplementary Figure 6: subgroup analysis of 24h urine protein quantification (region). Supplementary Figure 7: subgroup analysis of serum creatinine (course of treatment). Supplementary Figure 8: subgroup analysis of blood creatinine (region). Supplementary Figure 9: subgroup analysis of blood urea nitrogen (course of treatment). Supplementary Figure 10: subgroup analysis of blood urea nitrogen (region). Supplementary Figure 11: subgroup analysis of urinary albumin excretion rates (age). Supplementary Figure 12: subgroup analysis of urinary albumin excretion rates (course of treatment). Supplementary Figure 13: subgroup analysis of urinary albumin excretion rates (region). Supplementary Figure 14: subgroup analysis of fasting blood glucose (age). Supplementary Figure 15: subgroup analysis of fasting blood glucose (control treatment). Supplementary Figure 16: subgroup analysis of fasting blood glucose (course of treatment). Supplementary Figure 17: subgroup analysis of fasting blood glucose (region). Supplementary Figure 18: subgroup analysis of fasting blood glucose (adverse effects). Supplementary Figure 19: subgroup analysis of glycated hemoglobin (age). Supplementary Figure 20: subgroup analysis of glycated hemoglobin (region). Supplementary Figure 21: subgroup analysis of TC (age). Supplementary Figure 22: subgroup analysis of TC (control treatment). Supplementary Figure 23: subgroup analysis of TC (course of treatment). Supplementary Figure 24: subgroup analysis of TC (region). Supplementary Figu [file 1720749.f1.zip › Fig34 Subgroup analysis of total effective rate (control treatment).pdf]

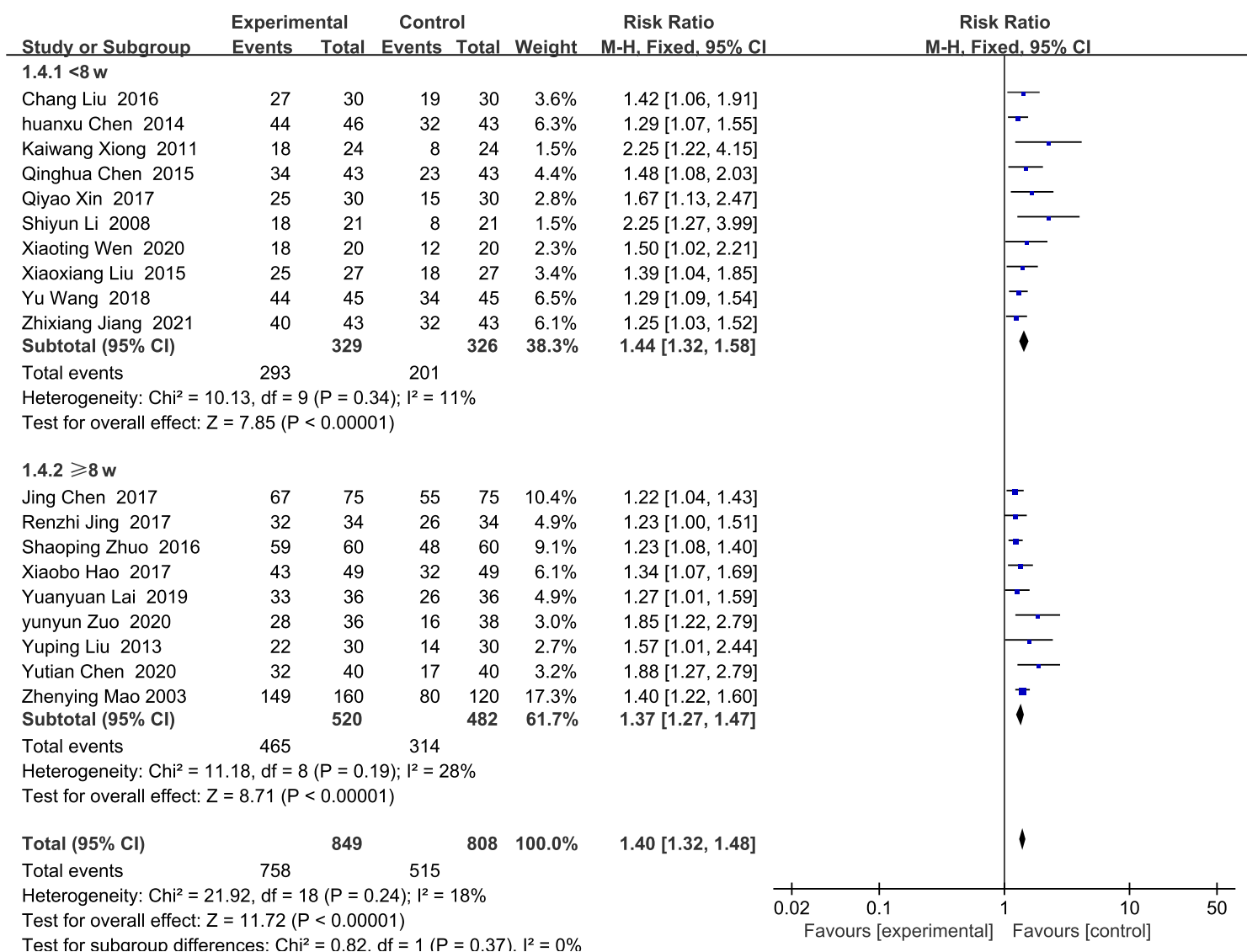

Supplement: Supplementary Materials — Supplementary Table 1: PubMed search strategy. Supplementary Figure 1: subgroup analysis of 24h urine volume (age). Supplementary Figure 2: subgroup analysis of 24h urine volume (region). Supplementary Figure 3: subgroup analysis of 24h urine protein quantification (age). Supplementary Figure 4: subgroup analysis of 24h urine protein quantification (control treatment). Supplementary Figure 5: subgroup analysis of 24h urine protein quantification (course of treatment). Supplementary Figure 6: subgroup analysis of 24h urine protein quantification (region). Supplementary Figure 7: subgroup analysis of serum creatinine (course of treatment). Supplementary Figure 8: subgroup analysis of blood creatinine (region). Supplementary Figure 9: subgroup analysis of blood urea nitrogen (course of treatment). Supplementary Figure 10: subgroup analysis of blood urea nitrogen (region). Supplementary Figure 11: subgroup analysis of urinary albumin excretion rates (age). Supplementary Figure 12: subgroup analysis of urinary albumin excretion rates (course of treatment). Supplementary Figure 13: subgroup analysis of urinary albumin excretion rates (region). Supplementary Figure 14: subgroup analysis of fasting blood glucose (age). Supplementary Figure 15: subgroup analysis of fasting blood glucose (control treatment). Supplementary Figure 16: subgroup analysis of fasting blood glucose (course of treatment). Supplementary Figure 17: subgroup analysis of fasting blood glucose (region). Supplementary Figure 18: subgroup analysis of fasting blood glucose (adverse effects). Supplementary Figure 19: subgroup analysis of glycated hemoglobin (age). Supplementary Figure 20: subgroup analysis of glycated hemoglobin (region). Supplementary Figure 21: subgroup analysis of TC (age). Supplementary Figure 22: subgroup analysis of TC (control treatment). Supplementary Figure 23: subgroup analysis of TC (course of treatment). Supplementary Figure 24: subgroup analysis of TC (region). Supplementary Figu [file 1720749.f1.zip › Fig35 Subgroup analysis of overall effectiveness (course of treatment).pdf]

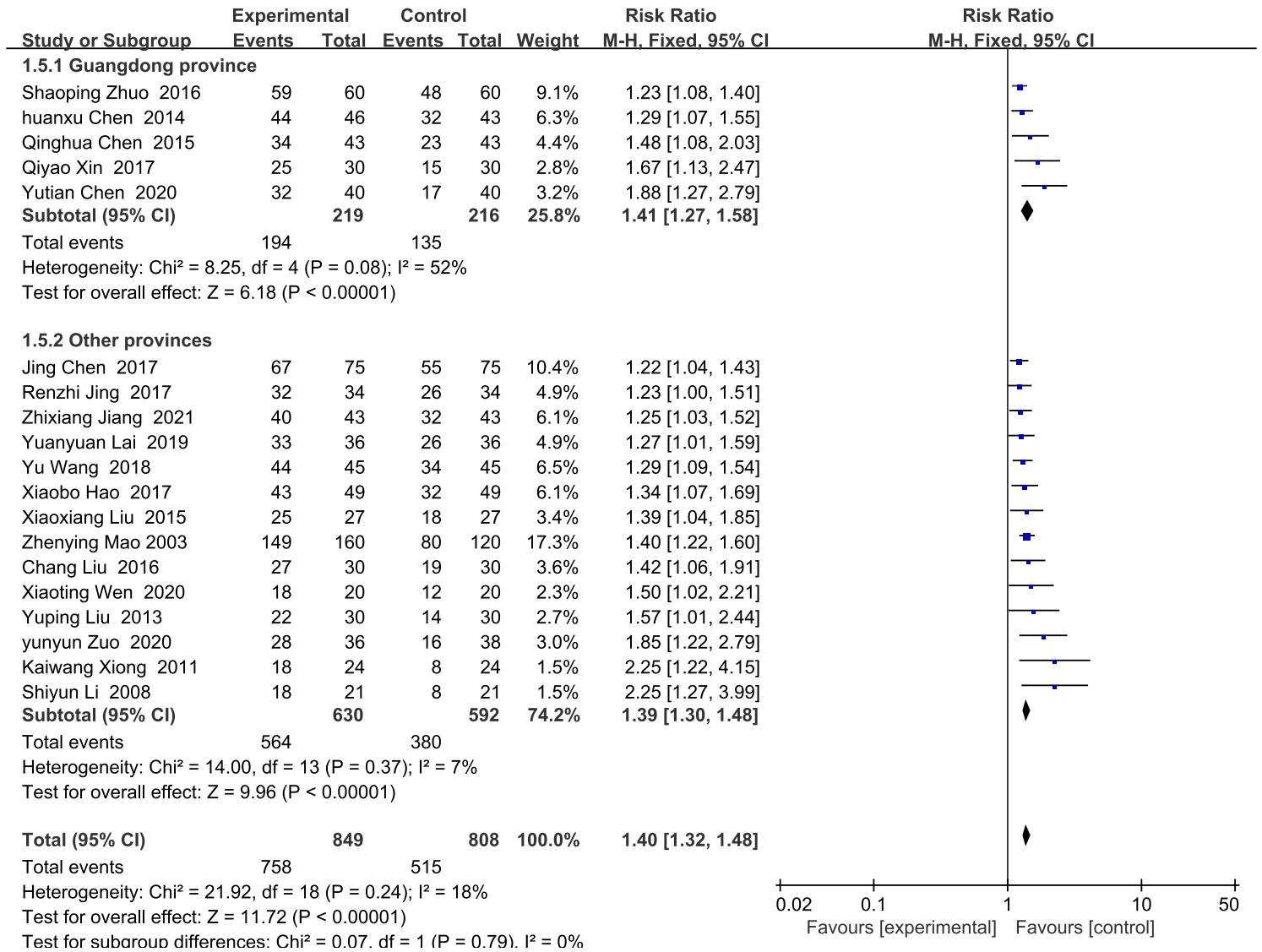

Supplement: Supplementary Materials — Supplementary Table 1: PubMed search strategy. Supplementary Figure 1: subgroup analysis of 24h urine volume (age). Supplementary Figure 2: subgroup analysis of 24h urine volume (region). Supplementary Figure 3: subgroup analysis of 24h urine protein quantification (age). Supplementary Figure 4: subgroup analysis of 24h urine protein quantification (control treatment). Supplementary Figure 5: subgroup analysis of 24h urine protein quantification (course of treatment). Supplementary Figure 6: subgroup analysis of 24h urine protein quantification (region). Supplementary Figure 7: subgroup analysis of serum creatinine (course of treatment). Supplementary Figure 8: subgroup analysis of blood creatinine (region). Supplementary Figure 9: subgroup analysis of blood urea nitrogen (course of treatment). Supplementary Figure 10: subgroup analysis of blood urea nitrogen (region). Supplementary Figure 11: subgroup analysis of urinary albumin excretion rates (age). Supplementary Figure 12: subgroup analysis of urinary albumin excretion rates (course of treatment). Supplementary Figure 13: subgroup analysis of urinary albumin excretion rates (region). Supplementary Figure 14: subgroup analysis of fasting blood glucose (age). Supplementary Figure 15: subgroup analysis of fasting blood glucose (control treatment). Supplementary Figure 16: subgroup analysis of fasting blood glucose (course of treatment). Supplementary Figure 17: subgroup analysis of fasting blood glucose (region). Supplementary Figure 18: subgroup analysis of fasting blood glucose (adverse effects). Supplementary Figure 19: subgroup analysis of glycated hemoglobin (age). Supplementary Figure 20: subgroup analysis of glycated hemoglobin (region). Supplementary Figure 21: subgroup analysis of TC (age). Supplementary Figure 22: subgroup analysis of TC (control treatment). Supplementary Figure 23: subgroup analysis of TC (course of treatment). Supplementary Figure 24: subgroup analysis of TC (region). Supplementary Figu [file 1720749.f1.zip › Fig36 Subgroup analysis of total efficiency (region).pdf]

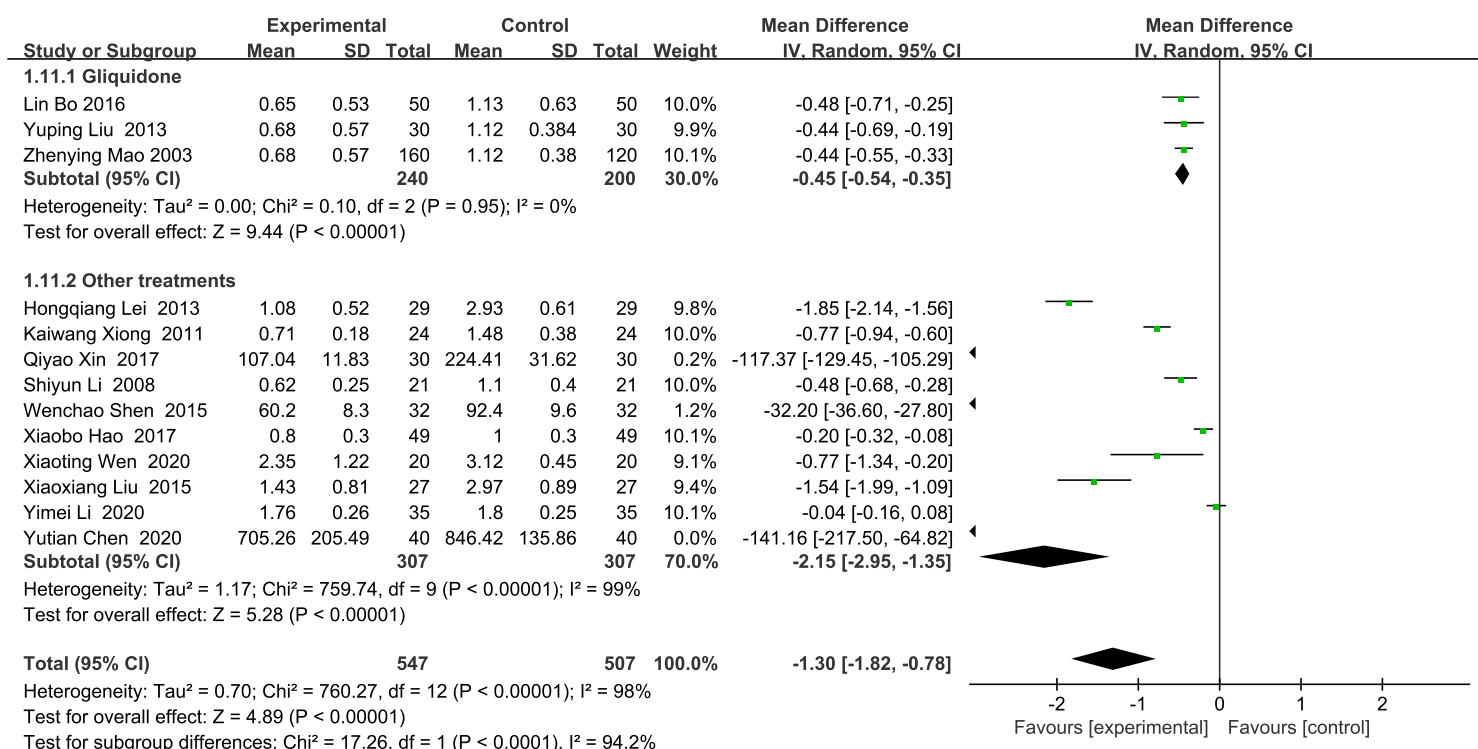

Supplement: Supplementary Materials — Supplementary Table 1: PubMed search strategy. Supplementary Figure 1: subgroup analysis of 24h urine volume (age). Supplementary Figure 2: subgroup analysis of 24h urine volume (region). Supplementary Figure 3: subgroup analysis of 24h urine protein quantification (age). Supplementary Figure 4: subgroup analysis of 24h urine protein quantification (control treatment). Supplementary Figure 5: subgroup analysis of 24h urine protein quantification (course of treatment). Supplementary Figure 6: subgroup analysis of 24h urine protein quantification (region). Supplementary Figure 7: subgroup analysis of serum creatinine (course of treatment). Supplementary Figure 8: subgroup analysis of blood creatinine (region). Supplementary Figure 9: subgroup analysis of blood urea nitrogen (course of treatment). Supplementary Figure 10: subgroup analysis of blood urea nitrogen (region). Supplementary Figure 11: subgroup analysis of urinary albumin excretion rates (age). Supplementary Figure 12: subgroup analysis of urinary albumin excretion rates (course of treatment). Supplementary Figure 13: subgroup analysis of urinary albumin excretion rates (region). Supplementary Figure 14: subgroup analysis of fasting blood glucose (age). Supplementary Figure 15: subgroup analysis of fasting blood glucose (control treatment). Supplementary Figure 16: subgroup analysis of fasting blood glucose (course of treatment). Supplementary Figure 17: subgroup analysis of fasting blood glucose (region). Supplementary Figure 18: subgroup analysis of fasting blood glucose (adverse effects). Supplementary Figure 19: subgroup analysis of glycated hemoglobin (age). Supplementary Figure 20: subgroup analysis of glycated hemoglobin (region). Supplementary Figure 21: subgroup analysis of TC (age). Supplementary Figure 22: subgroup analysis of TC (control treatment). Supplementary Figure 23: subgroup analysis of TC (course of treatment). Supplementary Figure 24: subgroup analysis of TC (region). Supplementary Figu [file 1720749.f1.zip › Fig4 Subgroup analysis of 24h urine protein quantification (control treatment).pdf]

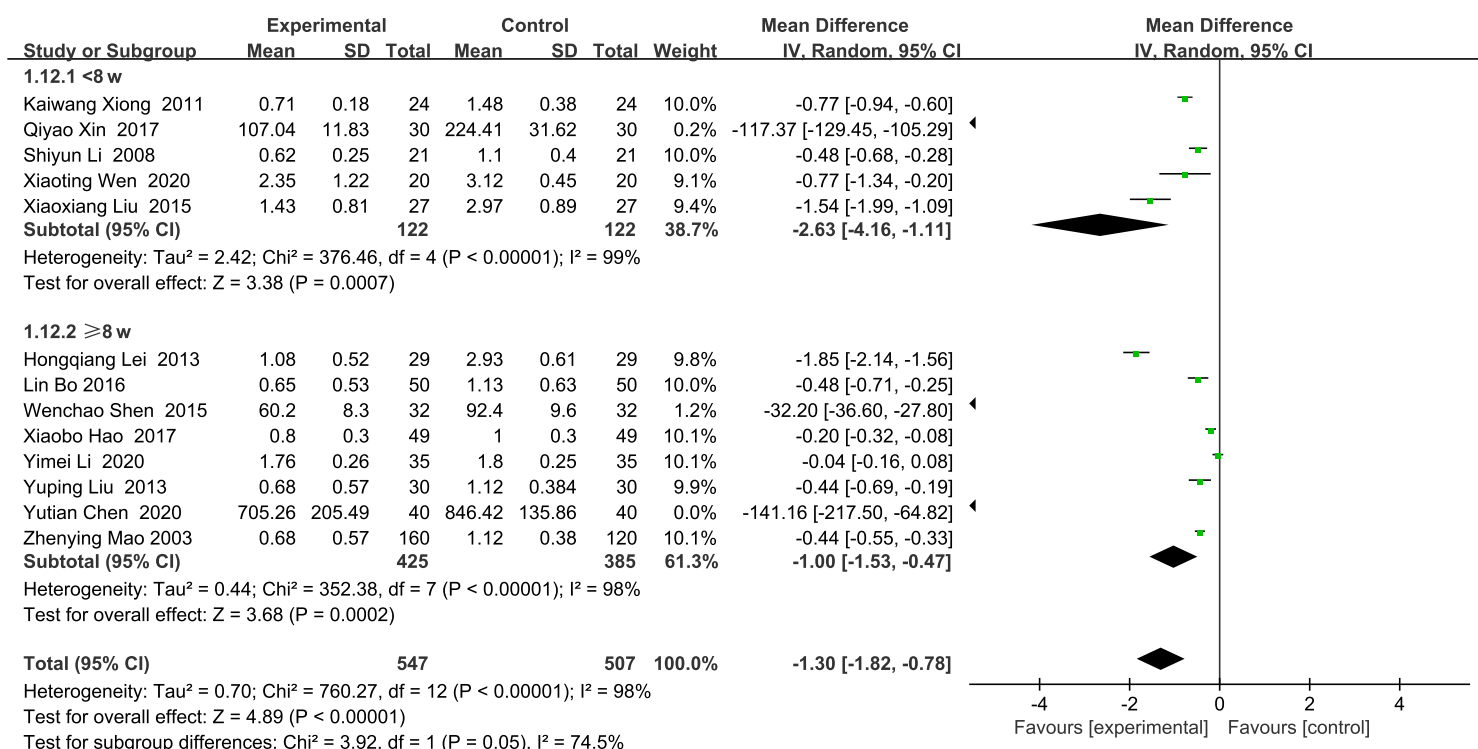

Supplement: Supplementary Materials — Supplementary Table 1: PubMed search strategy. Supplementary Figure 1: subgroup analysis of 24h urine volume (age). Supplementary Figure 2: subgroup analysis of 24h urine volume (region). Supplementary Figure 3: subgroup analysis of 24h urine protein quantification (age). Supplementary Figure 4: subgroup analysis of 24h urine protein quantification (control treatment). Supplementary Figure 5: subgroup analysis of 24h urine protein quantification (course of treatment). Supplementary Figure 6: subgroup analysis of 24h urine protein quantification (region). Supplementary Figure 7: subgroup analysis of serum creatinine (course of treatment). Supplementary Figure 8: subgroup analysis of blood creatinine (region). Supplementary Figure 9: subgroup analysis of blood urea nitrogen (course of treatment). Supplementary Figure 10: subgroup analysis of blood urea nitrogen (region). Supplementary Figure 11: subgroup analysis of urinary albumin excretion rates (age). Supplementary Figure 12: subgroup analysis of urinary albumin excretion rates (course of treatment). Supplementary Figure 13: subgroup analysis of urinary albumin excretion rates (region). Supplementary Figure 14: subgroup analysis of fasting blood glucose (age). Supplementary Figure 15: subgroup analysis of fasting blood glucose (control treatment). Supplementary Figure 16: subgroup analysis of fasting blood glucose (course of treatment). Supplementary Figure 17: subgroup analysis of fasting blood glucose (region). Supplementary Figure 18: subgroup analysis of fasting blood glucose (adverse effects). Supplementary Figure 19: subgroup analysis of glycated hemoglobin (age). Supplementary Figure 20: subgroup analysis of glycated hemoglobin (region). Supplementary Figure 21: subgroup analysis of TC (age). Supplementary Figure 22: subgroup analysis of TC (control treatment). Supplementary Figure 23: subgroup analysis of TC (course of treatment). Supplementary Figure 24: subgroup analysis of TC (region). Supplementary Figu [file 1720749.f1.zip › Fig5 Subgroup analysis of 24h urine protein quantification (course of treatment).pdf]

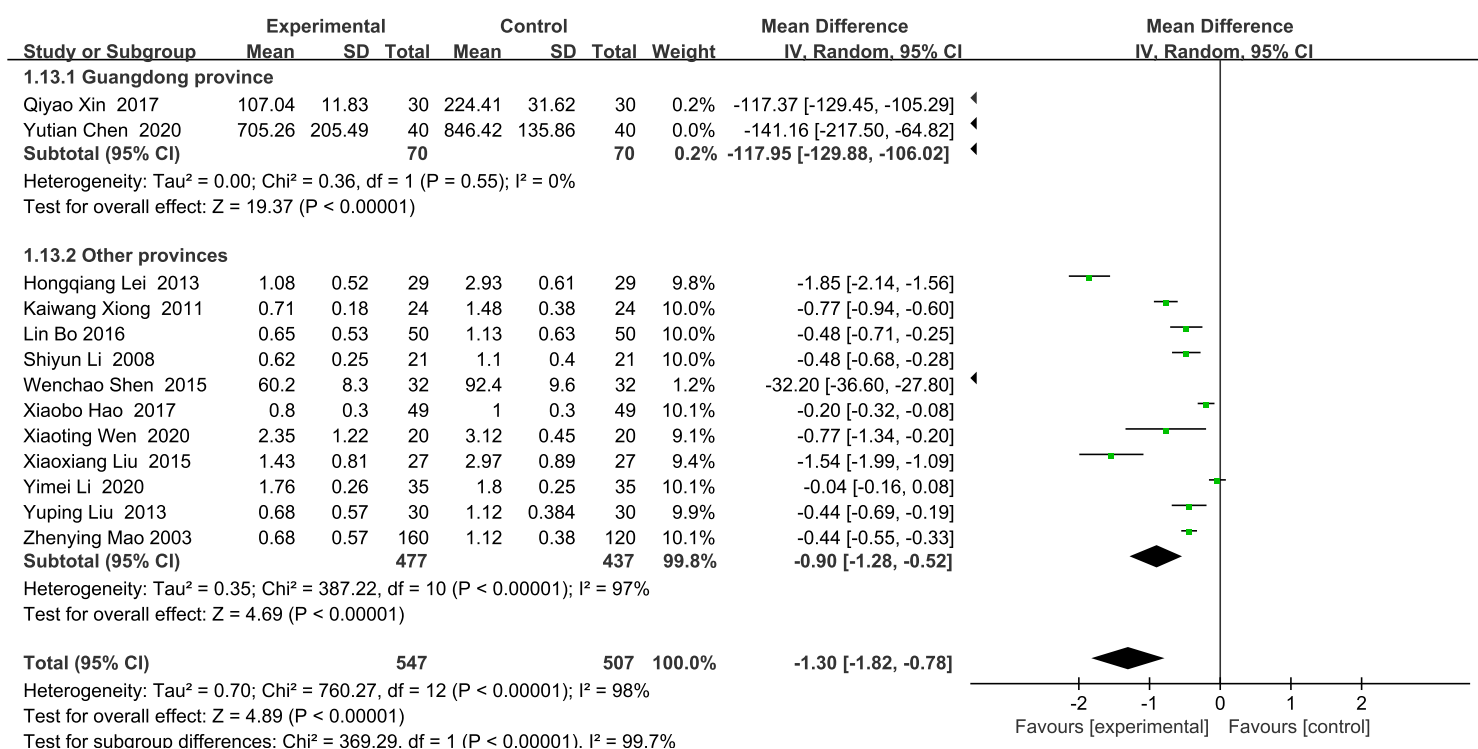

Supplement: Supplementary Materials — Supplementary Table 1: PubMed search strategy. Supplementary Figure 1: subgroup analysis of 24h urine volume (age). Supplementary Figure 2: subgroup analysis of 24h urine volume (region). Supplementary Figure 3: subgroup analysis of 24h urine protein quantification (age). Supplementary Figure 4: subgroup analysis of 24h urine protein quantification (control treatment). Supplementary Figure 5: subgroup analysis of 24h urine protein quantification (course of treatment). Supplementary Figure 6: subgroup analysis of 24h urine protein quantification (region). Supplementary Figure 7: subgroup analysis of serum creatinine (course of treatment). Supplementary Figure 8: subgroup analysis of blood creatinine (region). Supplementary Figure 9: subgroup analysis of blood urea nitrogen (course of treatment). Supplementary Figure 10: subgroup analysis of blood urea nitrogen (region). Supplementary Figure 11: subgroup analysis of urinary albumin excretion rates (age). Supplementary Figure 12: subgroup analysis of urinary albumin excretion rates (course of treatment). Supplementary Figure 13: subgroup analysis of urinary albumin excretion rates (region). Supplementary Figure 14: subgroup analysis of fasting blood glucose (age). Supplementary Figure 15: subgroup analysis of fasting blood glucose (control treatment). Supplementary Figure 16: subgroup analysis of fasting blood glucose (course of treatment). Supplementary Figure 17: subgroup analysis of fasting blood glucose (region). Supplementary Figure 18: subgroup analysis of fasting blood glucose (adverse effects). Supplementary Figure 19: subgroup analysis of glycated hemoglobin (age). Supplementary Figure 20: subgroup analysis of glycated hemoglobin (region). Supplementary Figure 21: subgroup analysis of TC (age). Supplementary Figure 22: subgroup analysis of TC (control treatment). Supplementary Figure 23: subgroup analysis of TC (course of treatment). Supplementary Figure 24: subgroup analysis of TC (region). Supplementary Figu [file 1720749.f1.zip › Fig6 Subgroup analysis of 24h urine protein quantification (region).pdf]

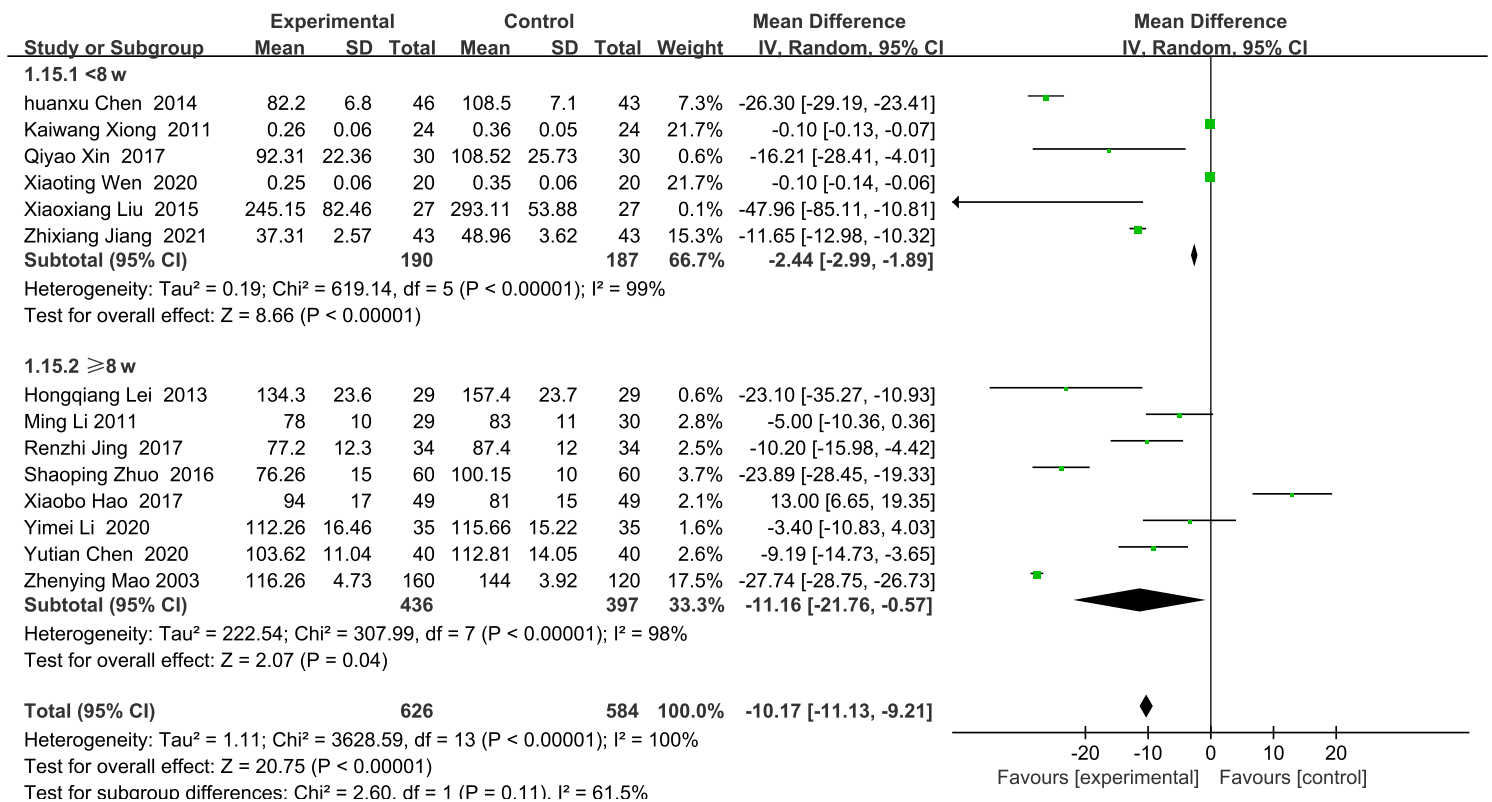

Supplement: Supplementary Materials — Supplementary Table 1: PubMed search strategy. Supplementary Figure 1: subgroup analysis of 24h urine volume (age). Supplementary Figure 2: subgroup analysis of 24h urine volume (region). Supplementary Figure 3: subgroup analysis of 24h urine protein quantification (age). Supplementary Figure 4: subgroup analysis of 24h urine protein quantification (control treatment). Supplementary Figure 5: subgroup analysis of 24h urine protein quantification (course of treatment). Supplementary Figure 6: subgroup analysis of 24h urine protein quantification (region). Supplementary Figure 7: subgroup analysis of serum creatinine (course of treatment). Supplementary Figure 8: subgroup analysis of blood creatinine (region). Supplementary Figure 9: subgroup analysis of blood urea nitrogen (course of treatment). Supplementary Figure 10: subgroup analysis of blood urea nitrogen (region). Supplementary Figure 11: subgroup analysis of urinary albumin excretion rates (age). Supplementary Figure 12: subgroup analysis of urinary albumin excretion rates (course of treatment). Supplementary Figure 13: subgroup analysis of urinary albumin excretion rates (region). Supplementary Figure 14: subgroup analysis of fasting blood glucose (age). Supplementary Figure 15: subgroup analysis of fasting blood glucose (control treatment). Supplementary Figure 16: subgroup analysis of fasting blood glucose (course of treatment). Supplementary Figure 17: subgroup analysis of fasting blood glucose (region). Supplementary Figure 18: subgroup analysis of fasting blood glucose (adverse effects). Supplementary Figure 19: subgroup analysis of glycated hemoglobin (age). Supplementary Figure 20: subgroup analysis of glycated hemoglobin (region). Supplementary Figure 21: subgroup analysis of TC (age). Supplementary Figure 22: subgroup analysis of TC (control treatment). Supplementary Figure 23: subgroup analysis of TC (course of treatment). Supplementary Figure 24: subgroup analysis of TC (region). Supplementary Figu [file 1720749.f1.zip › Fig7 Subgroup analysis of serum creatinine (course of treatment).pdf]

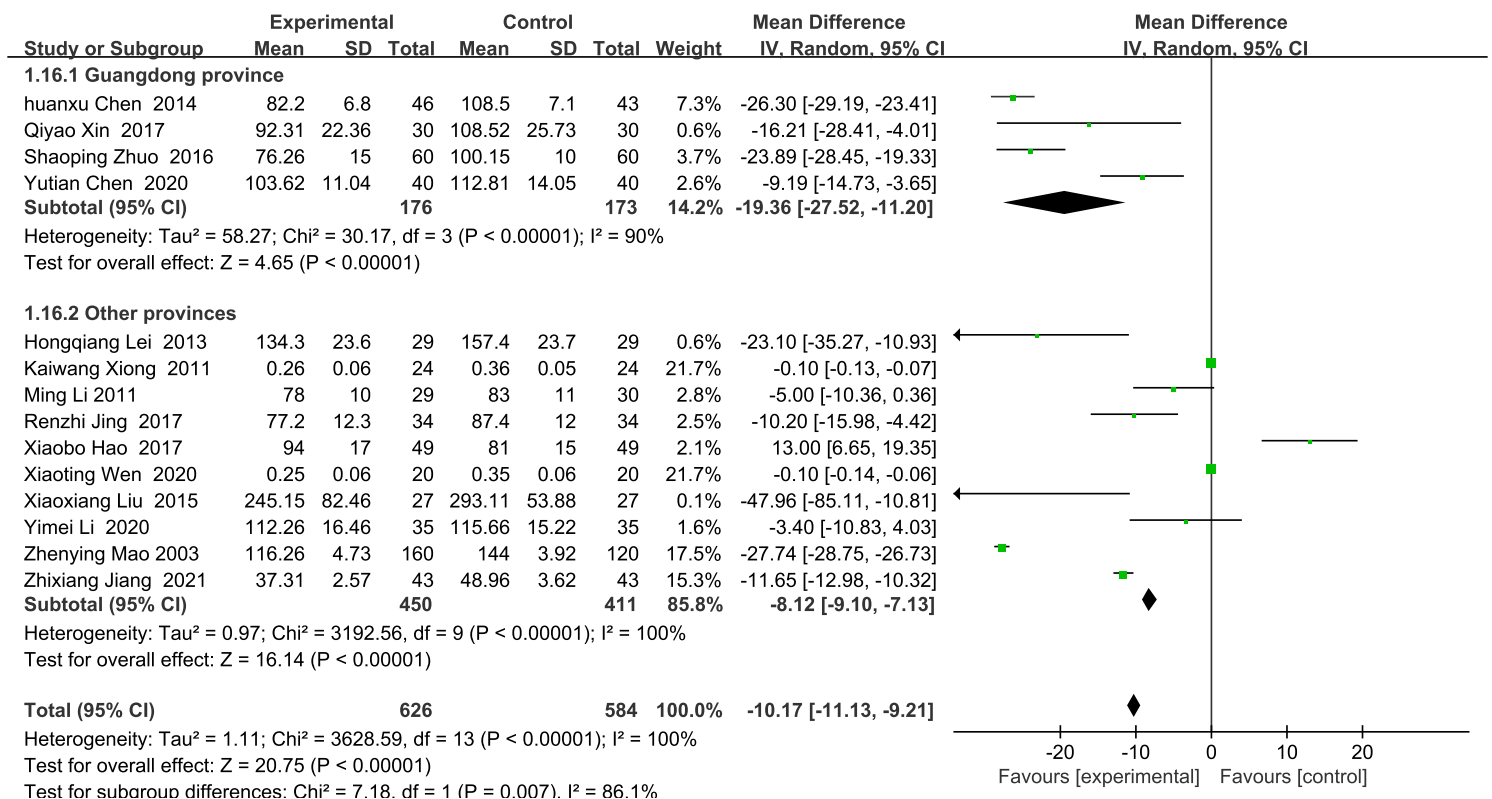

Supplement: Supplementary Materials — Supplementary Table 1: PubMed search strategy. Supplementary Figure 1: subgroup analysis of 24h urine volume (age). Supplementary Figure 2: subgroup analysis of 24h urine volume (region). Supplementary Figure 3: subgroup analysis of 24h urine protein quantification (age). Supplementary Figure 4: subgroup analysis of 24h urine protein quantification (control treatment). Supplementary Figure 5: subgroup analysis of 24h urine protein quantification (course of treatment). Supplementary Figure 6: subgroup analysis of 24h urine protein quantification (region). Supplementary Figure 7: subgroup analysis of serum creatinine (course of treatment). Supplementary Figure 8: subgroup analysis of blood creatinine (region). Supplementary Figure 9: subgroup analysis of blood urea nitrogen (course of treatment). Supplementary Figure 10: subgroup analysis of blood urea nitrogen (region). Supplementary Figure 11: subgroup analysis of urinary albumin excretion rates (age). Supplementary Figure 12: subgroup analysis of urinary albumin excretion rates (course of treatment). Supplementary Figure 13: subgroup analysis of urinary albumin excretion rates (region). Supplementary Figure 14: subgroup analysis of fasting blood glucose (age). Supplementary Figure 15: subgroup analysis of fasting blood glucose (control treatment). Supplementary Figure 16: subgroup analysis of fasting blood glucose (course of treatment). Supplementary Figure 17: subgroup analysis of fasting blood glucose (region). Supplementary Figure 18: subgroup analysis of fasting blood glucose (adverse effects). Supplementary Figure 19: subgroup analysis of glycated hemoglobin (age). Supplementary Figure 20: subgroup analysis of glycated hemoglobin (region). Supplementary Figure 21: subgroup analysis of TC (age). Supplementary Figure 22: subgroup analysis of TC (control treatment). Supplementary Figure 23: subgroup analysis of TC (course of treatment). Supplementary Figure 24: subgroup analysis of TC (region). Supplementary Figu [file 1720749.f1.zip › Fig8 Subgroup analysis of blood creatinine (region).pdf]

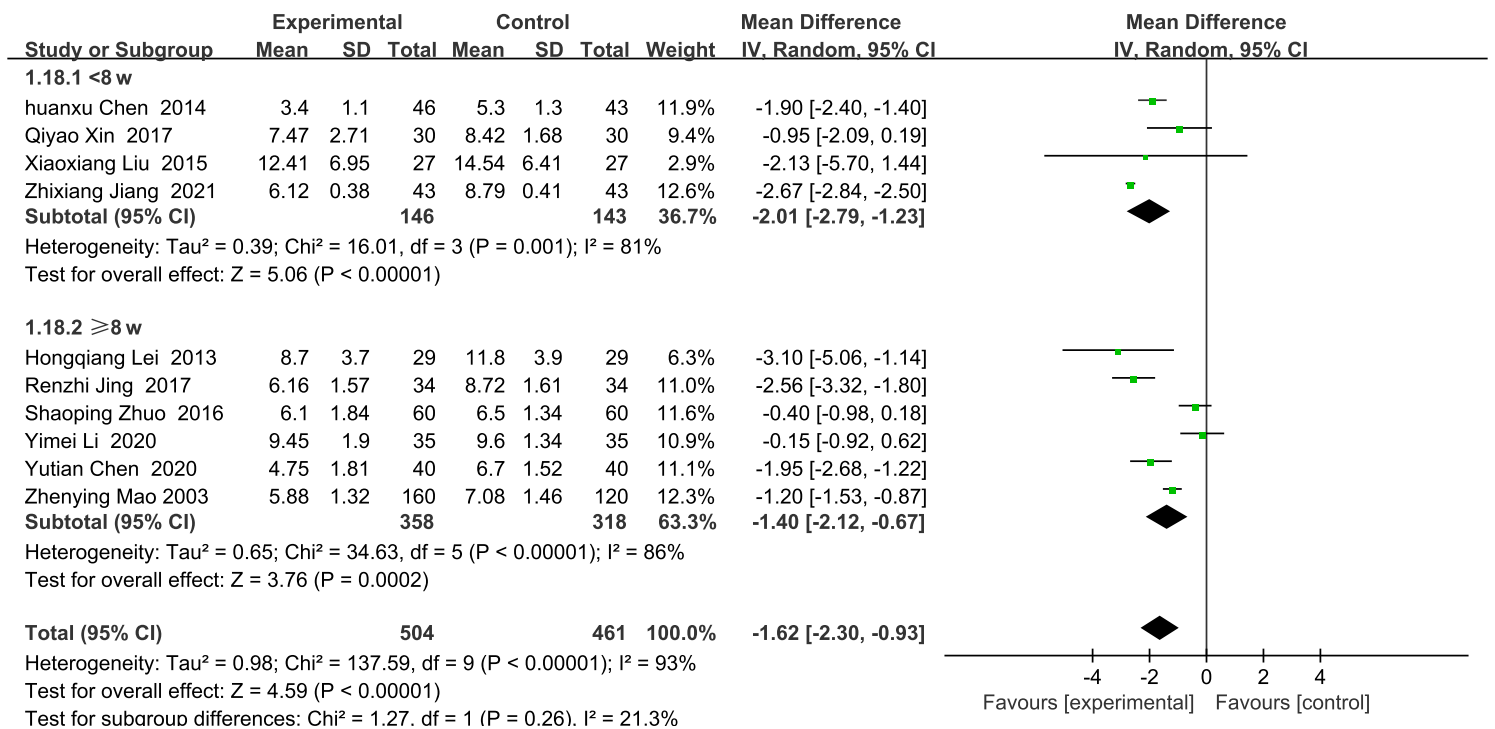

Supplement: Supplementary Materials — Supplementary Table 1: PubMed search strategy. Supplementary Figure 1: subgroup analysis of 24h urine volume (age). Supplementary Figure 2: subgroup analysis of 24h urine volume (region). Supplementary Figure 3: subgroup analysis of 24h urine protein quantification (age). Supplementary Figure 4: subgroup analysis of 24h urine protein quantification (control treatment). Supplementary Figure 5: subgroup analysis of 24h urine protein quantification (course of treatment). Supplementary Figure 6: subgroup analysis of 24h urine protein quantification (region). Supplementary Figure 7: subgroup analysis of serum creatinine (course of treatment). Supplementary Figure 8: subgroup analysis of blood creatinine (region). Supplementary Figure 9: subgroup analysis of blood urea nitrogen (course of treatment). Supplementary Figure 10: subgroup analysis of blood urea nitrogen (region). Supplementary Figure 11: subgroup analysis of urinary albumin excretion rates (age). Supplementary Figure 12: subgroup analysis of urinary albumin excretion rates (course of treatment). Supplementary Figure 13: subgroup analysis of urinary albumin excretion rates (region). Supplementary Figure 14: subgroup analysis of fasting blood glucose (age). Supplementary Figure 15: subgroup analysis of fasting blood glucose (control treatment). Supplementary Figure 16: subgroup analysis of fasting blood glucose (course of treatment). Supplementary Figure 17: subgroup analysis of fasting blood glucose (region). Supplementary Figure 18: subgroup analysis of fasting blood glucose (adverse effects). Supplementary Figure 19: subgroup analysis of glycated hemoglobin (age). Supplementary Figure 20: subgroup analysis of glycated hemoglobin (region). Supplementary Figure 21: subgroup analysis of TC (age). Supplementary Figure 22: subgroup analysis of TC (control treatment). Supplementary Figure 23: subgroup analysis of TC (course of treatment). Supplementary Figure 24: subgroup analysis of TC (region). Supplementary Figu [file 1720749.f1.zip › Fig9 Subgroup analysis of blood urea nitrogen (course of treatment).pdf]
